# One‐Pot Cooperation of Single‐Atom Rh and Ru Solid Catalysts for a Selective Tandem Olefin Isomerization‐Hydrosilylation Process

**DOI:** 10.1002/anie.201915255

**Published:** 2020-02-04

**Authors:** Bidyut B. Sarma, Jonglack Kim, Jonas Amsler, Giovanni Agostini, Claudia Weidenthaler, Norbert Pfänder, Raul Arenal, Patricia Concepción, Philipp Plessow, Felix Studt, Gonzalo Prieto

**Affiliations:** ^1^ Max-Planck-Institut für Kohlenforschung Kaiser-Wilhelm-Platz 1 45470 Mülheim an der Ruhr Germany; ^2^ Institute of Catalysis Research and Technology (IKFT) Karlsruhe Institute of Technology (KIT) Hermann-von-Helmholtz Platz 1 76344 Eggenstein-Leopoldshafen Germany; ^3^ Institute for Chemical Technology and Polymer Chemistry (ITCP) Karlsruhe Institute of Technology (KIT) Kaiserstrasse 12 76131 Karlsruhe Germany; ^4^ ALBA Synchrotron Light Source Carrer de la Llum 2–26, Cerdanyola del Vallès Barcelona Spain; ^5^ Max-Planck-Institut für Chemische Energiekonversion Stiftstrasse 34–36 45470 Mülheim an der Ruhr Germany; ^6^ Laboratorio de Microscopias Avanzadas (LMA) Instituto de Nanociencia de Aragon (INA) Universidad de Zaragoza Mariano Esquillor s/n 50018 Zaragoza Spain; ^7^ Instituto de Ciencias de Materiales de Aragon CSIC-Universidad de Zaragoza Pedro Cerbuna 12 50009 Zaragoza Spain; ^8^ ARAID Foundation 50018 Zaragoza Spain; ^9^ ITQ Instituto de Tecnología Química Universitat Politècnica de València-Consejo Superior de Investigaciones Científicas (UPV-CSIC) Av. Los Naranjos s/n 46022 Valencia Spain

**Keywords:** DFT calculations, olefin valorization, single-atom-catalysis, structure-performance relationships, tandem catalysis

## Abstract

Realizing the full potential of oxide‐supported single‐atom metal catalysts (SACs) is key to successfully bridge the gap between the fields of homogeneous and heterogeneous catalysis. Here we show that the one‐pot combination of Ru_1_/CeO_2_ and Rh_1_/CeO_2_ SACs enables a highly selective olefin isomerization‐hydrosilylation tandem process, hitherto restricted to molecular catalysts in solution. Individually, monoatomic Ru and Rh sites show a remarkable reaction specificity for olefin double‐bond migration and *anti*‐Markovnikov α‐olefin hydrosilylation, respectively. First‐principles DFT calculations ascribe such selectivity to differences in the binding strength of the olefin substrate to the monoatomic metal centers. The single‐pot cooperation of the two SACs allows the production of terminal organosilane compounds with high regio‐selectivity (>95 %) even from industrially‐relevant complex mixtures of terminal and internal olefins, alongside a straightforward catalyst recycling and reuse. These results demonstrate the significance of oxide‐supported single‐atom metal catalysts in tandem catalytic reactions, which are central for the intensification of chemical processes.

## Graphical abstract

**Singles in tandem**: The one‐pot integration of Ru_1_/CeO_2_ and Rh_1_/CeO_2_ single‐atom solid catalysts enables a highly selective olefin isomerization‐hydrosilylation tandem process for the conversion of terminal and internal olefins, as well as mixtures thereof, into industrially relevant terminal organosilane compounds.

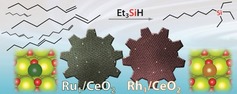

## Introduction

Isolated metal atoms stabilized on the surface of oxide carriers attract great attention as active sites in heterogeneous catalysis.^1^ Often referred to as single‐atom catalysts (SACs), these materials hold the potential to achieve a quantitative surface exposure of the supported metal for catalysis while displaying a higher site structural homogeneity ‐which is expected to translate into superior catalytic selectivity‐ compared to supported catalysts based on metal (oxide) clusters or nanoparticles. Atomically dispersed supported metals often exist in a cationic state, as their full reduction to a zero‐valent state implies that bonds to the oxide support are cleaved, which is typically followed by high adatom surface mobility and agglomeration even at relatively mild temperatures.^2^ Their cationic nature, monoatomicity and defined coordination environment make oxide‐supported SACs excellent candidates to bridge the gap between the disciplines of heterogeneous and homogeneous catalysis,^3^ particularly in a number of areas which have been traditionally dominated by molecular complex catalysts applied in solution. Hence, SACs have been recently explored for reactions classically catalyzed by cationic metal salts or complexes, including olefin hydroformylation,^5^ olefin hydrosilylation,^6^ alkyne hydrochlorination,^7^ or C−C coupling.^8^

A relevant area where SACs can have a profound impact is tandem catalysis, that is, the integration of two catalysts in a single pot to achieve sequential transformations in a direct manner.^9^ Tandem catalytic processes hold the promise for a multifold contribution to chemical process intensification by (i) circumventing the need for energy and cost‐intensive isolations of intermediate products; (ii) improving safety and selectivity by minimizing the residence time of highly reactive or unstable intermediate products in the reaction medium; and (iii) overcoming thermodynamic bounds to reaction yields, for example, driving reversible reactions to completion via the in situ processing of a reaction product in a subsequent irreversible catalytic step. The concept of tandem catalysis originated in the field of homogeneous catalysis with soluble molecular complexes.^11^ Initial approaches to combine two different catalysts in the same reaction medium, while preventing undesired (often self‐deactivating) mutual interactions, relied on the compartmentalization of the molecular catalysts in soft dendrimer or micelle nanocapsules.^12^ Oxide‐supported SACs hold the potential to inaugurate an advanced generation of tandem catalysts as they reconcile key features of most organometallic catalysts, that is, well‐defined monoatomic sites, with an intrinsic catalyst compartmentalization in non‐contacting solid matrices and a fully inorganic composition, which endows them with superior mechanical and thermal stability and facilitates catalyst reuse.

Herein we show that two solid catalysts based on Rh and Ru isolated metal atoms, respectively, stabilized on the surface of CeO_2_ create a synergetic effect when combined in a single pot, enabling a highly selective tandem olefin isomerization‐hydrosilylation process. Terminal organosilane compounds, which are of utmost technological significance in areas such as functional coatings, polymer cross‐linking and the manufacture of a wide variety of composite materials,^13^ can be produced with similarly high regio‐selectivities from both terminal and internal olefin substrates, as well as mixtures thereof.

## Results and Discussion

### Catalyst Synthesis and Characterization

Cerium oxide was applied as a support for the synthesis of SACs owing to its reported ability to stabilize transition metal cations at high temperatures.^14^ Initially, platinum was selected as active metal, given the established dominance of Pt molecular complexes as (pre)catalysts in conventional hydrosilylation processes.^15^ In addition, two series of materials were synthesized incorporating Rh and Ru, respectively. High metal dispersions on the CeO_2_ surface were induced via oxidative re‐dispersion at 1073 K in stagnant air.^16^ The nominal metal content, expressed hereafter as a surface‐specific loading (δ) that is, metal atoms per unit CeO_2_ surface area, was systematically adjusted within 0.2–10.0 M_at_ nm^−2^ (M=Pt, Rh or Ru).

**Figure 1:**
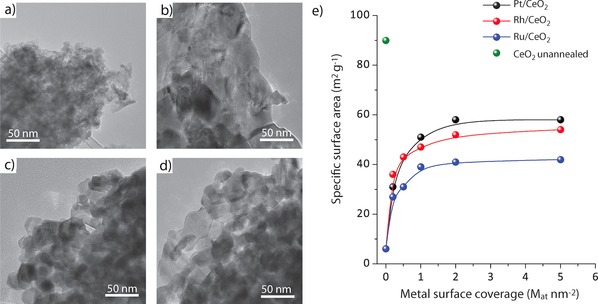
Synthesis of M/CeO_2_ catalysts by oxidative metal redispersion. Representative TEM micrographs for a) unannealed CeO_2_, b) CeO_2_ after annealing in air at 1073 K, c) 0.2 Rh/CeO_2_ catalyst, and d) 0.5 Rh/CeO_2_ catalyst. e) Evolution of the BET specific surface area with the surface‐specific metal content for the series of M/CeO_2_ catalysts. As a reference, the specific surface area for the unannealed CeO_2_ support is also included in the plot.

As illustrated in Figure 1, annealing of the metal‐free CeO_2_ support at 1073 K resulted in significant crystal sintering. Ceria nanocrystallites (5–20 nm) grew into larger (50–300 nm) and highly faceted crystals, resulting in a 15‐fold decrease in specific surface area (90 to 6 m^2^ g^−1^). However, the deposition of transition metals prior to annealing, already from very low metal contents, inhibits sintering and stabilizes smaller CeO_2_ crystals after annealing. Increasing the metal content from 0.2 M_at_ nm^−2^ to about 1.0–2.0 M_at_ nm^−2^ led to a progressive increment in S_BET_ up to 40–60 m^2^ g^−1^ (Figure 1 e). This trend, which was observed regardless of the identity of the metal deposited on CeO_2_, leveled off on further increasing metal content, attaining a plateau surface area. Analysis of the metal‐loaded materials by Raman spectroscopy provided evidences for the formation of M−O−Ce linkages upon annealing (Supporting Information, Figures S1–S3). Powder X‐ray diffraction showed no diffraction peaks other than those for CeO_2_ $\left(Fm\bar{3}m\right)$ at metal contents <2.0 M_at_ nm^−2^ for Pt/CeO_2_ and Ru/CeO_2_ catalysts, and <5.0 M_at_ nm^−2^ for Rh/CeO_2_ catalysts, respectively, indicating that at lower loadings metal species are highly dispersed as structures lacking long‐range order (Figures S4–S6). At higher metal contents, weak diffraction signals for Rh_2_O_3_ and RuO_2_ emerged for the series of Rh/CeO_2_ and Ru/CeO_2_, respectively, and increased in relative intensity with δ. In the case of Pt/CeO_2_ catalysts, sharp diffractions for metallic Pt^0^ were detected for δ>2.0 Pt nm^−2^, indicating also metal agglomeration and crystallization. It is hence inferred from these results that, regardless of the metal identity, metal species interact strongly with the CeO_2_ support upon high‐temperature annealing. The oxide surface energy is decreased, likely via the binding of metal species to high‐energy surface sites, and thus CeO_2_ sintering is hampered. Such stabilization is effective up to *δ*=1.0–2.0 M_at_ nm^−2^, beyond which further metal loading does not add to surface stabilization and metal agglomeration sets in, presumably due to the saturation of those binding sites on CeO_2_ which stabilize dispersed metal species.

X‐ray photoelectron spectroscopy (XPS) and X‐ray absorption near‐edge spectroscopy (XANES) proved that all metals exist in a cationic state in catalysts synthesized with metal contents at which no crystalline metal (oxide) species develop, that is, ≤2.0 M_at_ nm^−2^ (Figures S7–S10). Core electron binding energies could be ascribed to Pt^II^, Rh^III^, and Ru^IV^ formal oxidation states, respectively. Only in the case of Pt/CeO_2_ materials were additional contributions from Pt^IV^ oxide and metallic Pt^0^ species detected at metal contents >2.0 Pt nm^−2^, evidencing that Pt species aggregate into PtO_2_ at these metal loadings. This oxide is known to be unstable at the applied annealing temperature,^17^ and it thus decomposed partially into Pt^0^ crystals via the emission of lattice oxygen.

**Figure 2:**
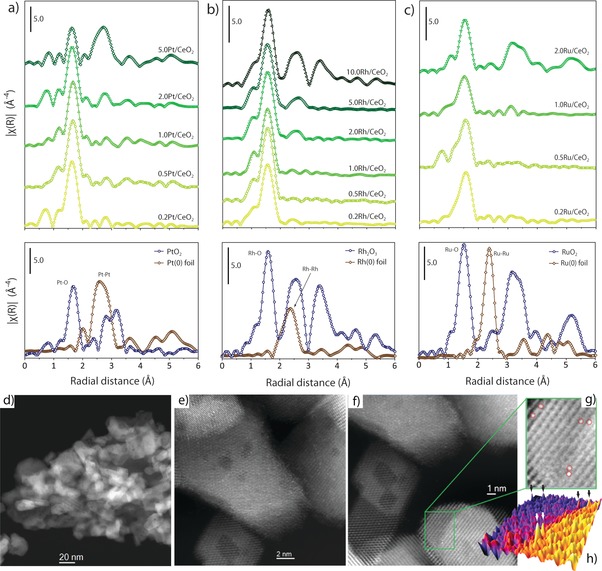
Atomicity of metal species in M/CeO_2_ catalysts. |FT| of the k^3^‐weighted *χ*(*k*) EXAFS function in R‐space for a) Pt/CeO_2_, b) Rh/CeO_2_ and c) Ru/CeO_2_ catalysts, as a function of the surface metal content (M_at_ nm^−2^). Radial distances are not phase‐corrected. See Figures S11–S13 for the corresponding spectra in *k*‐space. The spectra for bulk‐type metal oxides and metallic foils have also been included for reference. Amplitude scale bars are identical for catalysts and reference materials in each series. d–g) Representative C_s_‐HAADF‐STEM micrographs for 1.0 Pt/CeO_2_. Panel (g) shows a close‐up view of a nanoscale region in panel f, where isolated Pt atoms have been red‐circled as a guide to the eye. The corresponding 3D map of Z‐contrast for the same region is given in panel (h). Arrows point to the atomic‐size high‐Z‐contrast objects ascribed to isolated Pt atoms.

Extended X‐ray absorption fine structure (EXAFS) spectroscopy was applied to gain insight into the atomicity and coordination environment of metal species. Figure 2 shows the EXAFS spectra for M/CeO_2_ catalysts synthesized with various surface metal contents. As a reference, data for the corresponding bulk oxide and metal are also depicted. The corresponding spectra in *k*‐space are given in Figures S11–13. Regardless of the nature of the supported metal, no discernible second‐shell M−O−M scattering contributions (*r*>2 Å) could be inferred for catalysts with δ≤1.0 M_at_ nm^−2^, as an evidence for the existence of isolated metal atoms as the only metal species. Contributions from second‐shell M−O−M coordination became apparent at δ≥2.0 M_at_ nm^−2^, and increased in relative amplitude upon further increasing metal loading. In line with XRD results, this shows that a fraction of metal species aggregate into oxide (and metallic in the case of Pt) clusters beyond a metal content, which is somewhat metal‐dependent but in all cases >1.0 M_at_ nm^−2^. As EXAFS is sensitive also to species lacking long‐range atomic order, it reveals metal clustering already at contents at which no metal (oxide) crystallites were detectable by XRD, for example, *δ*=2.0 Rh_at_ nm^−2^ for Rh/CeO_2_ catalysts. The first‐shell *M*‐*O* average coordination number (CN) was minimum for *δ*=1.0 M_at_ nm^−2^ regardless of the metal nature, suggesting that isolated metal centers are maximum at this content (Tables S1–S3 and accompanying discussion in Supporting Information).

On the basis of our EXAFS results, a metal content of 1.0 M_at_ nm^−2^ was deemed to maximize the abundance of atomically dispersed species, while avoiding the coexistence of metal (oxide) aggregates. Therefore, selected catalysts with *δ*=1.0 M_at_ nm^−2^ were further investigated using Scanning‐Transmission Electron Microscopy (STEM) to get insights into the atomicity and spatial distribution of the metal species. As shown in Figures 2 f–h, isolated Pt atoms could be identified on CeO_2_ owing to their comparatively high Z‐contrast. On the contrary, Rh (like Ru) contribute a notably lower Z‐contrast, which precluded a direct visualization of metal atoms (Figure S14). Nevertheless, local EDX analysis proved the presence of Rh species in nanoscale areas where no other crystalline lattices aside that of the cubic CeO_2_ structure could be discerned. In agreement with EXAFS analysis, these results indicate that metal species did not form aggregates. Conversely, analysis of catalysts with δ>2.0 M_at_ nm^−2^, showed plainly the presence of metal (oxide) nanoparticles on the CeO_2_ surface (Figure S15). Collectively, our characterization results indicate that the synthesis of M/CeO_2_ SACs relies on an interplay between bulk (sub‐surface) and surface saturation phenomena. Catalysts exposing exclusively isolated metal atoms on their surface can be synthesized at an intermediate metal content of ca. 1.0 M_at_ nm^−2^, that is, high enough to achieve solid‐solution saturation, albeit low enough to prevent “saturation” of metal‐binding centers on the CeO_2_ surface, beyond which metal clustering occurs.

### Olefin Hydrosilylation Catalysis

In order to evaluate the catalytic performance of atomically dispersed metals, catalysts with 1.0 M_at_ nm^−2^ were tested in the hydrosilylation of 1‐octene in the presence of triethylsilane (Et_3_SiH) as silylating reagent. The results are compiled in Table 1 (entries 1–6). 1.0 Pt/CeO_2_ showed to be active, and reached near quantitative olefin conversion after 2 hours at 393 K. Nevertheless, the selectivity to internal olefins (58 %) exceeded that to the terminal 1,1,1‐triethyl‐1‐octylsilane (40 %), indicating that olefin isomerization and hydrosilylation pathways occur at comparable rates on this catalyst. In marked contrast, with 1.0 Rh/CeO_2_ the reaction proceeded to almost full olefin conversion (98 %), remarkably with 96 % selectivity to the anti‐Markovnikov terminal octylsilane product. In the absence of Et_3_SiH, under otherwise identical reaction conditions, 1.0 Rh/CeO_2_ afforded only 16 % olefin conversion to isomer products, whereas 1.0 Pt/CeO_2_ led to negligible 1‐octene conversion. 1.0 Ru/CeO_2_ displayed barely any reactivity in the absence of Et_3_SiH. However, under hydrosilylation conditions, it proved remarkably selective towards olefin isomerization, affording 99 % selectivity to internal olefins. On the one hand, these results show that active sites for olefin isomerization develop in the presence of the hydrosilane reactant. On the other hand, they reveal vast differences in performance for the different metals atomically dispersed on CeO_2_, as a result of differences in the relative reaction rates for hydrosilylation and isomerization pathways.

**Table 1:** Catalytic results for the hydrosilylation of 1**‐**octene with different catalysts. 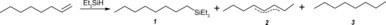

Entry	Catalyst	Silane	*T* [K]	*t* [h]	X^[a]^ [%]	Product selectivity [%]		
						***1***	***2***	***3***
1^[b]^	1.0 Rh/CeO_2_	Et_3_SiH	393	2	98	96	4	–
2	1.0 Rh/CeO_2_	–	393	2	n.d.^[f]^	–	–	–
3	1.0 Pt/CeO_2_	Et_3_SiH	393	2	99	40	58	2
4	1.0 Pt/CeO_2_	–	393	2	n.d.	–	–	–
5	1.0 Ru/CeO_2_	Et_3_SiH	393	2	99	1	99	–
6	1.0 Ru/CeO_2_	–	393	2	<1	–	–	–
7	5.0 Rh/CeO_2_	Et_3_SiH	393	5	99	94	6	–
8	10Rh/CeO_2_	Et_3_SiH	393	5	53	93	6	1
9	1.0 Rh/CeO_2_^[c]^	Et_3_SiH	393	5	99	81	19	–
10	Rh_2_O_3_^[d]^	Et_3_SiH	393	5	79	97	2	1
11	Rh/C^[e]^	Et_3_SiH	393	5	40	70	27	3

Reaction Conditions: 1‐octene (5 mmol ), triethylsilane (5 mmol), catalyst (2 μmol, metal basis), *P*=10 bar (N_2_, 99.999 % purity). [a] Olefin conversion. [b] The ^1^H NMR spectra of the crude product is given in Figure S16 (Supporting Information). [c] Catalyst activated by reduction at 623 K in flow of 20 % H_2_/N_2_. [d] As received from Sigma–Aldrich (99.8 % purity). [e] As received from Sigma–Aldrich, 5 wt %Rh. [f] n.d.: not detected.

**Figure 3:**
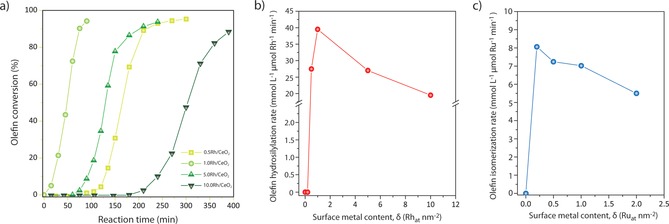
Metal speciation‐dependent catalytic performance. a) Time‐resolved evolution of the olefin conversion in the hydrosilylation of 1‐octene with Et_3_SiH employing Rh/CeO_2_ catalysts synthesized with different surface metal content. b) Dependence of the initial metal‐specific olefin hydrosilylation rate (extrapolated to conversion onset) of 1‐octene with Et_3_SiH with the surface‐specific Rh content for Rh/CeO_2_ catalysts. c) Dependence of the initial metal‐specific olefin isomerization rate for 1‐octene under olefin hydrosilylation reaction conditions with the surface‐specific Ru content for Ru/CeO_2_ catalysts. Reaction conditions: 1‐octene (5 mmol), triethylsilane (5 mmol), catalyst (2 μmol, Rh or Ru metal basis), *P*=10 bar (N_2_, 99.999 % purity), *T*=393 K.

Encouraged by the excellent olefin hydrosilylation performance exhibited by 1.0 Rh/CeO_2_, additional studies followed to ascertain the nature of the optimal rhodium active sites. First, the performance of Rh/CeO_2_ catalysts was studied as a function of δ. No activity was detected with 0.2 Rh/CeO_2_, presumably due to the fact that a majority of the Rh atoms are coordinatively saturated, for example, in sub‐surface positions, and thus not accessible to reactants as suggested by CO‐FTIR spectroscopy (Figure S17). Catalysts with higher Rh contents (0.5–10 Rh_at_ nm^−2^) proved to be active. In all cases, a reaction induction period was observed, whose duration was a function of δ (Figure 3 a). Activating the material in H_2_ prior to catalysis eliminated this induction period (Figure S18), whereas experiments with deuterated Et_3_Si‐D led to longer induction times under otherwise identical reaction settings. Slurry‐phase EXAFS spectroscopy applied on 1.0 Rh/CeO_2_ prior to and after catalysis induction discarded Rh dimerization or oligomerization as processes which precede activity (Figure S19). Similarly to what has been proposed for molecular catalysts,^18^ the reaction induction period can thus be ascribed to a slow (partial) reduction of the metal centers, that is, the cleavage of Rh−O bonds, necessary for the oxidative addition of the hydrosilane reagent. The duration of this induction period correlated with the average Rh−O coordination number derived from EXAFS analysis (Figure S20), which suggests that Rh centers in higher coordination positions, for example, most stable (confined) surface sites on ceria at the lowest metal contents or RhO_*x*_ clusters at the highest metal contents, are more difficult to activate for catalysis via Rh−O bond cleavage. As depicted in Figure 3 b, following the induction period, the Rh‐specific initial hydrosilylation reaction rate showed a volcano dependence with the metal content. A maximum activity was registered for *δ*=1.0 Rh_at_ nm^−2^, a coverage which is hence inferred to maximize the density of the most active sites. Further increasing the Rh content led to a progressive increase in the induction period alongside a decline in reaction rate. This finding suggests that polynuclear Rh oxide clusters, which exist on the catalyst surface at δ>2.0 Rh_at_ nm^−2^, are notably less effective sources of active sites. In all cases, similarly high selectivities (>90 %) to the terminal octylsilane were obtained, indicating that differences in catalytic performance arise from differences in the number of effective active sites rather than in their intrinsic behavior.

To elucidate the optimal Rh speciation for catalysis, various catalysts bearing Rh species with different oxidation state and nuclearity were tested (Table 1, entries 7–11). Tests with 10 Rh/CeO_2_ and Rh_2_O_3_, both bearing polynuclear Rh^III^ oxide species, resulted in not only lower activity and/or selectivity to the terminal silane compared to 1.0 Rh/CeO_2_, but also noticeable leaching of rhodium into the liquid reaction medium. Moreover tests with catalysts bearing metallic Rh^0^ nanoparticles, either supported on CeO_2_ (1.0 Rh/CeO_2_ pre‐reduced in H_2_ at 623 K) or on a rather inert carbon carrier (commercial 5 wt % Rh/C), led to lower reaction rates and, most notably, greater selectivities to undesired internal olefin isomerization products by a factor of >4. These findings suggest that, even though reduction treatments might eliminate catalysis induction, the presence of metallic Rh^0^ species in the activated catalyst undesirably enhances the rate of olefin isomerization over that of hydrosilylation. This is in keeping with previous studies which have associated an unbalanced olefin isomerization activity to active centers on metallic nanoparticles^19^ or nascent polynuclear metallic clusters in solution originating from the decomposition of molecular catalysts.^20^ Taken together, these results furnish evidence that atomically dispersed Rh^δ+^ species, whose contribution on the surface of CeO_2_ is maximized at a metal content of ≈1.0 Rh_at_ nm^−2^, are optimal sites for hydrosilylation. Moreover, atomically dispersed Rh/CeO_2_ displays also a remarkable substrate scope and tolerance to various functional groups in the α‐olefin substrates (Table S4).

### Tandem Olefin Isomerization‐Hydrosilylation Catalysis

In view of the different activities exhibited by SACs based on different metals for olefin isomerization and hydrosilylation pathways, it stood to reason to study the potential of these catalysts for a tandem olefin isomerization‐hydrosilylation process. Whereas the production of technologically relevant terminal silanes via hydrosilylation of α‐olefins is typically uncomplicated and highly selective, particularly with optimized molecular catalysts, a tandem catalytic conversion is highly desired to achieve the selective conversion of more challenging, unconventional olefin feedstocks, for example, those derived from paraffin dehydrogenation,^21^ the Fischer–Tropsch synthesis^22^ or cross‐metathesis upgrading processes from low‐value ethylene oligomerization products,^23^ which typically consist of complex mixtures of various olefin regio‐isomers. Essential, in this case, is to integrate olefin double‐bond migration and hydrosilylation activities in a single reaction medium. Moreover, whilst these reactions often compete on the same active sites under hydrosilylation conditions,15c, 18, 24 in this case they should ideally reside on independent and non‐interacting active sites, so that their relative reaction rates can be independently adjusted in order to optimize the selectivity and time‐yield of the tandem process to the desired terminal silane products. The combination of different organometallic complexes in a single pot has been reported to be an effective approach to accomplish a tandem (dehydrogenative) isomerization‐hydrosilylation process.^25^ However, next to those issues intrinsically associated to homogeneous catalysis, that is, catalyst recovery and recycling, the long‐term stability of the molecular catalysts as well as the minimization of hydrogenation and isomerization side‐products remain genuine challenges.

Studies with the herein developed solid SACs on 2‐propen‐1‐ol as substrate proved that olefin hydrosilylation proceeds >30 times faster than isomerization on isolated Rh sites (Figure S21 and Table S5). On the contrary, Ru centers in 1.0Ru/CeO_2_ are exceptionally selective for olefin isomerization under hydrosilylation conditions. For Ru/CeO_2_ catalysts the metal‐specific reaction rate showed an evolution with the metal surface content which is qualitatively similar to that observed for olefin hydrosilylation on Rh/CeO_2_ catalysts (Figure 3 c). In this case, however, no induction period was observed, pointing to a kinetically more facile development of the active Ru species after contact with the Et_3_SiH reactant (Figure S22). Moreover, full reactivity was observed already from *δ*=0.2 Ru_at_ nm^−2^, and retained up to *δ*=1.0 Ru_at_ nm^−2^, that is, the compositional range for which Ru is atomically dispersed, suggesting that the fraction of metal atoms in sub‐surface positions, and thus inaccessible for catalysis, is lower than for the Rh/CeO_2_ system. The reaction rate decreased for Ru contents >1.0 Ru_at_ nm^−2^, in parallel to the development of aggregated Ru^IV^ oxide species on the catalyst surface. It is hence inferred that, also in this case, atomically dispersed Ru on the CeO_2_ surface is the most efficient active site.

**Figure 4:**
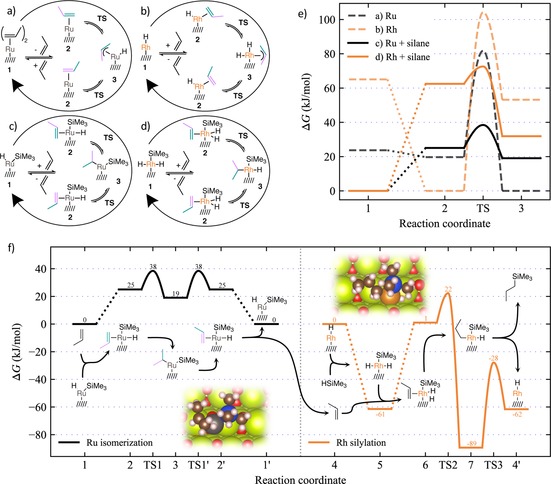
DFT calculations of the reaction mechanisms. a–d) Computed reaction pathways and e) the corresponding free energy diagrams with the most stable state set to zero in each case. Comparison of olefin isomerization on Ru_1_/CeO_2_ and Rh_1_/CeO_2_ single‐atom sites stabilized at the step‐edge of the corrugated CeO_2_ (211) surface, prior to (a,b) and after (c,d) activation by oxidative addition of HSiMe_3_. f) Computed free energy diagram of the tandem olefin isomerization/hydrosilylation process. Olefin isomerization is catalyzed by Ru_1_/CeO_2_ while subsequent hydrosilylation with HSiMe_3_ is catalyzed by Rh_1_/CeO_2_ single‐atom sites. Reactants considered: propene as model olefin, HSiMe_3_ as silylating agent. *T*=393 K, *P*=10 bar (see computational details in the SI).

Density functional theory (DFT) calculations at the PBE‐D3 level of theory (with *U*=5 eV to describe the Ce 4f electrons) were performed to get molecular level insight into the reaction specificity displayed by single‐atom Rh/CeO_2_ and Ru/CeO_2_ catalysts for olefin hydrosilylation and isomerization pathways, respectively. We use a CeO_2_(211) surface, that connects two (111) facets of ceria, to model the stoichiometric type II edge (Figure S23). This step edge has been previously described as an adsorption site for atomically dispersed Pt on CeO_2_ surfaces.^26^ Isomerization pathways of propene as the model olefin were compared for Ru_1_/CeO_2_(211) and Rh_1_/CeO_2_(211) sites (see Figure S24 for the optimized active site structures). The results are given in Figures 4 a–e. In line with experimental results, which show the presence of Et_3_SiH in the reaction medium to be essential for reactivity, the effective energy barriers for isomerization, at a temperature of 393 K, decreased by 44 kJ mol^−1^ for Ru_1_ and by 31 kJ mol^−1^ for Rh_1_ when oxidative insertion of the hydrosilane on the monatomic sites preceded olefin binding. After activation of the metal centers by silane addition, the lowest effective reaction energy barrier of 38 kJ mol^−1^ was found for Ru_1_/CeO_2_(211), that is, about 34 kJ mol^−1^ lower than that computed for the Rh analog. Since the intrinsic energy barriers for olefin insertion into the metal hydride bond (2→TS) are very similar for Ru and Rh centers (13 vs. 10 kJ mol^−1^), the effective barrier height is mainly determined by the binding strength of the olefin substrate to Ru and Rh. This is in agreement with scaling relations previously proposed by Wodrich et al.^27^ who found that Ru complexes tend to bind to olefin substrates stronger than Rh counterparts. These computational findings provide an explanation for the notably higher olefin isomerization activity observed experimentally for 1.0 Ru/CeO_2_.

Having identified the foundations for the role of Ru in olefin isomerization, the second half of the tandem reaction was investigated next. Figure 4 f shows the free energies computed for elementary steps across the entire tandem process, that is, olefin isomerization on Ru_1_/CeO_2_, and subsequent hydrosilylation catalyzed by Rh_1_/CeO_2_. The calculated hydrosilylation pathway was consistent with the so‐called Chalk‐Harrod mechanism involving RhH species as the resting state of the catalytic center.^24^ This active species was found to be energetically feasible, and in line with various experimental observations, for example, an induction period for the reaction, which is eliminated upon pre‐treatment with H_2_ (vide supra). Experiments with 1‐octene as olefin reactant and isotopically labelled Et_3_Si‐D as silylating agent showed the incorporation of deuterium in various carbon positions in the terminal silane product, as well as its scrambling within four carbon atoms in the octene isomer byproducts (Figure S25), suggesting the hydrosilane as the hydride source and the reversibility of olefin addition to the Rh sites.

Alternative reaction mechanisms previously proposed in literature,^28^ and which assume olefin coordination to precede oxidative addition of the hydrosilane reagent, were also explored, but were found to be subjected to comparatively higher overall free energy barriers (Figure S26). On the basis of the computational results, the olefin hydrosilylation rate is proposed to be limited by olefin insertion, with an effective barrier of 84 kJ mol^−1^, while the reductive elimination of the organosilane product is predicted with a lower barrier of 62 kJ mol^−1^.

The performance of the two combined SACs in the tandem process was assessed via the conversion of internal olefin substrates and the results are summarized in Table 2. Under the standard reaction conditions applied herein (393 K), the application of 1.0 Rh/CeO_2_ on 2‐octene resulted in a 33 % yield to silanes after 18 hours, with a 96 % selectivity to the terminal octylsilane product. This result reveals that the minor olefin double‐bond migration activity of the Rh‐based single‐atom catalyst contributes to the conversion of 2‐octene. The Ru‐based counterpart, on the contrary, showed barely any activity towards hydrosilylation, in line with the performance observed with α‐olefins. Remarkably, the one‐pot combination of 1.0 Ru/CeO_2_ and 1.0 Rh/CeO_2_ catalysts increased the reaction yield to 70 % (for an equimolar Ru/Rh ratio) and 80 % (for Ru/Rh=2), notably while preserving a high selectivity in excess of 92 % to the terminal organosilane. Additional experiments showed that this slight drop in selectivity was not related to the tandem approach but simply due to the much higher conversion degree achieved (Figure S27). These results highlight the synergistic effect achieved by the integration of the complementary olefin isomerization and hydrosilylation reactivities of each catalyst, respectively, in a single pot. The performance level achieved via the tandem combination of Ru‐ and Rh‐based SACs was beyond reach for the Pt‐based counterpart, which showed comparable rates for olefin isomerization and hydrosilylation routes (Table 1 and Table S5), either alone or in combination with 1.0 Ru/CeO_2_, or a conventional Pt‐based molecular Karstedt's catalyst (Table 2, entries 6–8). These results are in line with the lower performance expected when olefin double‐bond chain‐walking and terminal hydrosilylation routes compete (at similar rates) on the same active sites ‐as previously observed for molecular Pt catalysts‐15c, 18 which makes olefin on‐site residence time less effective and it thus lowers hydrosilylation turnover frequencies.

**Table 2:** Catalytic results for the tandem olefin isomerization/hydrosilylation of internal olefins and olefin isomer mixtures. 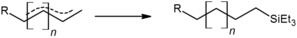

Entry	Catalyst	Olefin	Silane	*T* [K]	*t* [h]	Y^[a]^ [%]	T:B Silane^[b]^ (−)
1	1.0 Rh/CeO_2_	2‐octene	Et_3_SiH	393	18	33^[k]^	96:4^[k]^
2	1.0 Ru/CeO_2_	2‐octene	Et_3_SiH	393	18	<1	–
3	1.0 Rh/CeO_2_ + 1.0 Ru/CeO_2_	2‐octene	Et_3_SiH	393	18	70	93:7
4	^[c]^1.0 Rh/CeO_2_ + 1.0 Ru/CeO_2_	2‐octene	Et_3_SiH	393	18	80	92:8
5	1.0 Rh/CeO_2_ + 1.0 Ru/CeO_2_	2‐hexene	Et_3_SiH	393	18	73	97:3
6	1.0 Pt/CeO_2_	2‐octene	Et_3_SiH	393	18	39	96:4
7	1.0 Pt/CeO_2_ + 1.0 Ru/CeO_2_	2‐octene	Et_3_SiH	393	18	49	93:7
8	Pt Karstedt catalyst^[e]^	2‐octene	Et_3_SiH	393	18	10	98:2
9	1.0 Rh/CeO_2_	3‐octene	Et_3_SiH	393	18	5	96:4
10	1.0 Ru/CeO_2_	3‐octene	Et_3_SiH	393	18	<1	–
11	1.0 Rh/CeO_2_ + 1.0 Ru/CeO_2_	3‐octene	Et_3_SiH	393	18	20	91:9
12	^[c]^1.0 Rh/CeO_2_ + 1.0 Ru/CeO_2_	3‐octene	Et_3_SiH	393	18	29	90:10
13	^[d]^1.0 Rh/CeO_2_ + 1.0 Ru/CeO_2_	3‐octene	Et_3_SiH	393	18	32	89:11
14	1.0 Rh/CeO_2_ + 1.0 Ru/CeO_2_	3‐octene	Et_3_SiH	413	18	50	88:12
15	1.0 Rh/CeO_2_ + 1.0 Ru/CeO_2_	4‐octene	Et_3_SiH	413	18	37	82:18
16	1.0 Rh/CeO_2_ + 1.0 Ru/CeO_2_	*trans*‐Propenylbenzene	Et_3_SiH	393	18	50	98:2
17	1.0 Rh/CeO_2_ + 1.0 Ru/CeO_2_	8‐bromooctene isomers mix^[f]^	Et_3_SiH	393	18	70	91:9
18	1.0 Rh/CeO_2_ + 1.0 Ru/CeO_2_^[g]^	Octene isomers mix^[h]^	Et_3_SiH	393	18	83	95:5
19	1.0 Rh/CeO_2_ + 1.0 Ru/CeO_2_^[i]^	Neodene® 8/9/10 isomers mix^[j]^	Et_3_SiH	393	18	43	96:4

Reaction Conditions: olefin (5 mmol), triethylsilane (5 mmol), catalyst (4 μmol (total metal basis) unless otherwise stated), P=10 bar (N_2_, 99.999 % purity). For tests combining two catalysts, equimolar amounts of the two metals were applied, unless otherwise indicated with footnotes. For additional catalytic results, that is, full screening of Ru/Rh molar ratio for the tandem isomerization/hydrosilylation of 3‐octene, see Figure S28 in the Supporting Information. [a] Yield to silanes (remaining products are olefin isomers). [b] Terminal‐to‐branched molar ratio within organosilane products. [c] Ru/Rh molar ratio of 2.0. [d] Ru/Rh molar ratio of 4.0. [e] Commercially available platinum(0)‐1,3‐divinyl‐1,1,3,3‐tetramethyldisiloxane (Sigma–Aldrich). [f] Isomers mixture generated from the corresponding α‐olefin (8‐bromooct‐1‐ene) by reaction with 1.0Ru/CeO_2_ as catalyst. [g] Olefin (5 mmol, excluding n‐octane), triethylsilane (5 mmol), catalyst (Ru/Rh molar ratio of 2). [h] Octene isomers/n‐octane mixture (20 % Octane, 42 % 1‐Octene, 38 % 2‐Octene (*cis+trans*)) representative of the crude olefin product obtained by transfer dehydrogenation of *n*‐octane employing a state‐of‐the‐art Ir‐based pincer catalyst (see main text). [i] Reaction conditions: industrial olefin mixture (≈25 mmol), triethylsilane (25 mmol), catalyst (30 μmol of metal, Ru/Rh molar ratio of 2.0), *P*=10 bar (N_2_, 99.999 % purity). [j] Industrial internal/terminal olefin mixture, containing mainly C_8_‐C_10_ linear olefins, produced as part of the shell higher olefins process (SHOP) and associated olefin redistributon unitary operations (see main text). [k] The standard error for yield and L:B selectivity was ±2 % and ±1 %, respectively, as determined from 3 independent tests for selected reaction conditions.

The performance of the tandem reaction on 2‐octene prompted us to assess the conversion of a further internal *n*‐olefin such as 3‐octene, which is both more challenging as a substrate when terminal regioselectivity is sought after, and more representative of target industrial olefin feedstocks derived for example, from metathetic olefin redistribution processes on oligomerization educts, which contain significant shares of 3‐*ene* and further internal olefins. In this case, the use of either of the Rh‐ or Ru‐based SACs alone resulted in barely any reactivity (yield to organosilanes ≤5 % after 18 h, Table 2 entries 9 and 10), as a result of their poor individual activity for olefin double‐bond migration and hydrosilylation pathways, respectively. Remarkably, the tandem combination of the two catalysts achieved a significant activity on this substrate. As shown in Figure S28, a full screening of the tandem metal composition enlightened a clear volcano dependence of the organosilane yield with the Ru/Rh molar relative abundance. A maximum yield of 32 % after 18 h was achieved with an optimal Ru/Rh ratio of 4.0, while ≥90 % selectivity to the terminal silane product was retained within the entire compositional range studied. On the one hand, the optimal catalyst blending, notably enriched in Ru, is a consequence of the significantly higher intrinsic (metal‐specific) activity of 1.0 Rh/CeO_2_ for hydrosylilation of α‐olefins compared to that of 1.0 Ru/CeO_2_ for double‐bond isomerization (Figure 3 b,c). On the other hand, the >6‐fold increase in product yield compared to single‐catalyst tests (Figure S28), clearly illustrates how the tandem system opens the door to a process which is beyond reach for either of the two catalysts individually. Excellent results were also obtained via the cooperation of the two single‐atom catalysts on sterically more hindered substrates such as *trans*‐propenylbenzene (50 % yield with 98 % selectivity to terminal silane, entry 16). Reaction yields could be increased by a factor of 3, and the conversion extended to further internal olefins such as 4‐octene, by increasing the reaction temperature to 413 K, in this case with comparatively lower product regioselectivities, yet in excess of 80 % (entries 14 and 15). This catalytic system proved also efficient to selectively convert complex mixtures of terminal and internal olefins into terminal organosilanes. A mixture of 8‐bromooctene isomers, generated by isomerization of the corresponding α‐olefin (8‐bromo‐1‐octene) with 1.0 Ru/CeO_2_, was converted with 91 % selectivity to the terminal 1,1,1‐triethyl‐8‐bromooctylsilane. Similarly high regioselectivities were also achieved from industrially relevant olefin mixtures, representative for output streams from mild‐temperature paraffin dehydrogenation processes,^4^ and olefin oligomerization/metathesis operations, as in the commercial Shell Higher Olefin Process®.^10^, ^23^ In all cases, the cooperation of Ru/CeO_2_ and Rh/CeO_2_ single‐atom catalysts in tandem led to a highly selective (>95 %) production of terminal organosilanes (Table 2, entries 18 and 19). The tandem process relies on the in situ processing of terminal olefins ‐generated by the isomerization catalyst‐ further on the hydrosilylation active catalyst, and its efficiency cannot be paired by a two‐step process where a first, independent isomerization step favors olefin mixtures enriched in the thermodynamically most stable internal isomers.

Hot filtration tests evidenced the absence of further catalytic activity (within experimental error) after the solid catalysts had been removed from the reaction medium under operation conditions, underpinning the heterogeneous character of the tandem reaction. Moreover, no noticeable decrease in either activity or selectivity was observed for at least 5 consecutive tandem reaction cycles without any intermediate catalyst rejuvenation/regeneration treatment (Figure S29). EXAFS analysis of the solid catalysts after the sequence of reaction batches showed no evidences of second‐shell M−O−M coordination scattering (Figure S29), whereas no metal clusters or nanoparticles could be visualized by C_s_‐HAADF‐STEM (Figure S30). These observations provide strong evidence for the perseverance of the atomically isolated metal centers and their stability against clustering.

## Conclusion

In summary, our results demonstrate that the single‐pot cooperation of CeO_2_‐supported Ru and Rh‐based single‐atom catalysts realizes a tandem catalytic process which is capable of reconciling the reaction specificity ‐and thus chemical orthogonality‐ typical of molecular catalysts with the stability and technically uncomplicated recycling inherent to solid catalysts. The technological significance of this approach is herein demonstrated with the direct and selective conversion of complex mixtures of olefin regio‐isomers to terminal organosilanes. Beyond this showcase process, our results provide a blueprint to exploit a new dimension of oxide‐supported single‐atom metal catalysts, in the area of tandem catalysis, which can decisively contribute to realize their potential as a bridge between the realms of homogeneous and heterogeneous catalysis.

## Conflict of interest

The authors declare no conflict of interest.

## Supporting information

As a service to our authors and readers, this journal provides supporting information supplied by the authors. Such materials are peer reviewed and may be re‐organized for online delivery, but are not copy‐edited or typeset. Technical support issues arising from supporting information (other than missing files) should be addressed to the authors.

Supplementary
