# Supplementary material for: One‐Pot Cooperation of Single‐Atom Rh and Ru Solid Catalysts for a Selective Tandem Olefin Isomerization‐Hydrosilylation Process
Source: Angew Chem Int Ed Engl. 2020 Feb 4;59(14):5806–15. doi: 10.1002/anie.201915255 (PMC7154713; doi:10.1002/anie.201915255)
Supplement: Supplementary file 1 — Supplementary [file ANIE-59-5806-s001.pdf]

Angewandte Chemie
Eine Zeitschrift der Gesellschaft Deutscher Chemiker

Supporting Information

One-Pot Cooperation of Single-Atom Rh and Ru Solid Catalysts for a Selective Tandem Olefin Isomerization-Hydrosilylation Process

*Bidyut B. Sarma, Jonglack Kim, Jonas Amsler, Giovanni Agostini, Claudia Weidenthaler, Norbert Pfänder, Raul Arenal, Patricia Concepción, Philipp Plessow, Felix Studt, and Gonzalo Prieto**

anie_201915255_sm_miscellaneous_information.pdf

Table of contents

	Page
**1. Experimental methods**	S3
Materials	S3
Synthesis of CeO_2	S3
Synthesis of CeO_2-supported metal catalysts	S3
Chemical analysis	S4
N_2-physisorption	S4
Powder X-ray diffraction (XRD)	S4
Raman Spectroscopy	S5
X-ray photoelectron spectroscopy (XPS)	S5
Transmission electron microscopy (TEM)	S5
Scanning-transmission electron microscopy (STEM) and energy-dispersive X-ray (EDX) spectroscopy	S5
X-Ray Absorption Spectroscopy (XAS)	S6
Infrared spectroscopy coupled to CO as surface probe	S7
Catalysis	S8
**2. Computational details**	S10
**3. Supplementary Figures**	S11
**4. Supplementary Tables**	S42
**5. References**	S47
**6. Optimized DFT structures**	S48

1. Experimental methods

Materials

For catalyst synthesis, $Ce(NO_3)_3.6H_2O$ (99.99%), rhodium(III) acetylacetonate (97%), platinum(II) acetylacetonate (97%), ruthenium(III) acetylacetonate (97%) and acetone (>99.9%) were purchased from Sigma Aldrich and used as received. Reference materials Rh_2O_3 (99.8%) and 5 wt% Rh/C were also purchased from Sigma-Aldrich. For catalytic tests, triethylsilane (99%), 1-octene (98%), 2-octene (97%), styrene (>99%), 1-hexene (>99%), allylbenzene (98%), 8-Bromo-1-Octene (97%), 6-Chloro-1-hexene (96%), 5-Hexene-2-on (99%), and *trans*-propenylbenzene (99%) from Sigma-Aldrich, and 2-propen-1-ol (99%), 6-Heptenenitrile (96%), 3-octene (98%), 4-octene (97%), 4-Hexen-1-ol (97%) and allyl triethoxysilane (97%) from Alfa-Aesar were also used as received, without further purifications. An sample of the industrial olefin mixture Neodene® 8910 was kindly provided by Shell (Amsterdam, The Netherlands) and applied as reaction substrate as received.

Synthesis of CeO_2

Nanosized CeO_2 with nanopolyhedra primary crystal morphology was synthesized according to a published procedure.[1] $Ce(NO_3)_3$ $6H_2O$ was calcined in air at 350^oC for 2 h, producing a yellow powder. The powder was then ground in a mortar.

Synthesis of CeO_2-supported metal catalysts

For the synthesis of M/CeO_2 catalysts, the corresponding metal acetylacetonate was dissolved in acetone (>99.9%, Sigma Aldrich). Next, CeO_2 was dispersed into this solution under stirring and the solvent was removed slowly by evaporation under dynamic vacuum (430 mbar) until dryness at 313 K. The solid was further dried at 353 K in an oven overnight and ground into a powder. The resulting powder was calcined in an uncapped porcelain dish (50 mm diameter) at 1073 K under a stagnant air atmosphere for 10 hours using a temperature ramp from RT of 2 K min^{-1}. In all cases, the metal content was adjusted to

achieve pre-selected surface-specific metal loadings (after the high-temperature annealing treatment) in the range of 0.2-10 M_{at} nm^{-2}.

Chemical analysis

The overall (bulk) metal loading on the M/CeO_2 catalysts (M=Pt, Rh, Ru) was determined by means of Energy Dispersive X-ray (EDX) spectroscopy in a Hitachi TM3030 scanning electron microscope. The samples were finely ground in a mortar and the resulting powder applied on a pin-stub SEM mount coated with a double-adhesive-face conductive carbon-tab. Areas of 1 mm^2 were analyzed and the resulting EDX spectra collected with an Oxford Pentafet 10 mm^2 detector. Experimentally determined surface specific metal contents showed deviations <(±18%) for Pt/CeO_2 and Rh/CeO_2 catalysts. In the case of Ru/CeO_2 catalysts, experimental contents <33% lower than the nominal ones were determined, suggesting the partial emission of volatile Ru sub-oxides during annealing. The experimental error of the EDX-based analysis was estimated to be ca. ±10%, based on independent analyses of selected standard samples. Nominal metal surface contents have been used for sample notation throughout the manuscript.

N_2-physisorption

Nitrogen physisorption isotherms were recorded at 77 K using a Micromeritics 3Flex V4.04 instrument. Prior to the measurement, samples were dried at 423 K under vacuum for 5 h. Surface areas were derived using the B.E.T method in the relative pressure (P/P_0) regime of 0.05-0.30.

Powder X-ray diffraction (XRD)

Powder X-ray powder patterns were collected on a Stoe STADI P transmission diffractometer equipped with a primary Ge (111) monochromator ($MoK\alpha_1$) and a position-sensitive detector. Data were collected in the 2θ range between 5 and 50° with a step width of 0.015° and a measuring time per step of 20 s. For each sample, 8 scans were collected and summed up

after data collection. For the measurements, the samples were filled into glass capillaries (∅ 0.5 mm). Acquired patterns were evaluated qualitatively by comparison with entries from the ICCD PDF-2 powder pattern database.

Raman Spectroscopy

Raman spectra were acquired with a Renishaw *in via* REFLEX spectrometer equipped with a CCD detector and coupled to a Leica microscope. The laser beam was focused on the sample by means of a 20x (f=0.40) Leica objectives. The experiments were performed under ambient conditions in a back-scattering geometry. The samples were irradiated with a (Nd:YVO_4) laser (λ=532 nm) and the spectra were acquired in a single scan with 10 s accumulation time.

X-ray photoelectron spectroscopy (XPS)

XPS experiments were performed in a Kratos HSi spectrometer with a hemispherical analyzer. The monochromatic AlK_{α} X-ray (E=1486.6 eV) was operated at 15 kV and 15 mA. An analyzer pass energy of 40 eV was applied for the narrow scans. Hybrid mode was used as lens mode. The base pressure during the operation in the analysis chamber was 4×10^{-7} Pa. Binding energy values were referred to C1s at 284.5 eV.

Transmission electron microscopy (TEM)

Bright-field transmission electron micrographs were registered using a Hitachi HF-2000 microscope operated at 200 kV. Prior to observation, powder samples were dry-cast on Cu grids coated with a lacey carbon film.

Scanning-transmission electron microscopy (STEM) and energy-dispersive X-ray (EDX) spectroscopy

High-angle annular dark-field Scanning-transmission electron (HAADF-STEM) micrographs and Energy-dispersive X-ray spectroscopy (EDX) elemental maps were acquired in a probe-

corrected FEI Titan Low-Base microscope fitted with a X-FEG® gun and C_s-probe corrector CESCOR from CEOS GmbH and operated at 300 keV (convergent semi-angle of 25 mrad), and a C_s probe-corrected Hitachi HD-2700 microscope equipped with a cold field-emission gun and two EDAX Octane T Ultra W EDX detectors and operated at 200 kV. Powder samples or ethanol suspensions thereof were cast on Cu grids coated with a lacey carbon film prior to observation.

X-Ray Absorption Spectroscopy (XAS)

X-Ray absorption spectra were recorded at the Rh K-edge (23.220 keV), Ru K-edge (22.117 keV) and Pt LIII-edge (11.564 keV), respectively, at the CLÆSS beamline station (BL22) of the ALBA synchrotron light source, Barcelona (Spain).[2] The beam was monochromatized using a (311) double crystal monochromator and harmonic rejection was performed using Pt-coated silicon mirrors for Rh and Ru edges and Rh-coated silicon mirrors for Pt edge. Samples in the form of self-supported pellets were hosted inside a multipurpose gas-solid cell previously described by Guilera et al.[3] Measurements were performed at room temperature in fluorescence mode using a fluorescence solid-state Silicon Drift Detector. Reference high-purity metal oxide materials were pressed into self-supported pellets (Ø=31mm) with optimized thickness after dilution in powder boron nitride and measured in transmission mode employing ion chambers filled with appropriate gases in order to adsorb at the different energies 15% and 80% in the I_0 and I_1, respectively. In order to investigate structural modifications in the metal species following the catalysis induction period, slurry-phase XAS experiments were performed on a selected Rh/CeO_2 catalyst (1.0 Rh_{at} nm^{-2}) at the CLAESS beam-station of the ALBA Synchrotron Light Source. Experimentally, catalysis conditions were simulated *ex situ* in a high-pressure Schott Duran® glass reactor equipped with a pressure gauge and magnetic stirring. The reactor was flushed with high-purity N_2 and then either only *n*-dodecane (blank experiment), 3 mmol of Et_3SiH diluted in *n*-dodecane, or equimolar amounts (3 mmol) of Et_3SiH and 1-dodecene (olefin substrate) and ca. 20 mg of catalyst were added. The C_{12} substrate/solvent were selected for these tests as their lower

vapor pressure at the reaction temperature (393 K) was preferred to lower the autogenous pressure and thus meet the safety measures prescribed at the synchrotron beamline station. The reactor was sealed and heated under vigorous magnetic stirring to 393 K and kept for 1 h at this temperature under autogenous pressure. At the end of the test performed with both Et_3SiH and 1-dodecene hydrosilylation reactants, the formation of the corresponding 1,1,1-triethyl-1-dodecylsilane product, in a yield >30%, and thus the surpassing of the catalysis induction period, was confirmed by gas chromatography coupled to mass spectrometry (after diluting an aliquot of the reactor´s liquid phase in toluene). Next, the reactor was cooled down to room temperature and transferred to a N_2-filled glove-bag. An aliquot of the slurry sample was transferred under exclusion of air into a ca. 100 μL polymer cell equipped with Kapton® foil windows and the EXAFS spectra of the slurry recorded at the Rh-K absorption edge at room temperature in fluorescence mode. At least 3 scans were acquired for each sample to ensure spectral reproducibility and good signal-to-noise ratio. The data reduction and extraction of the χ(k) function has been performed using Athena code.[4] EXAFS data analysis has been performed using Artemis software.[4]

Infrared spectroscopy coupled to CO as surface probe

Fourier-Transform Infrared (FTIR) spectra of adsorbed CO were recorded at low temperature (110 K) with a Nexus 8700 FTIR spectrometer using a DTGS detector and acquiring at 4 cm^{-1} resolution. An IR cell allowing in situ treatments in controlled atmospheres and temperatures from 97 K to 773 K has been connected to a vacuum system with a high-precision gas dosing facility. The samples were pressed into self-supported wafers (16-21 mg) and treated at 973 K in stagnant air in a muffle oven for 10 h, then transferred to the IR cell and further treated at 573 K under flow of pure O_2 for 3 h in order to remove (hydrogen)carbonate species –which form on exposure and handling of the sample in presence of atmospheric CO_2– from the catalyst surface. The decarbonation treatment was followed by evacuation at 10^{-4} mbar at 473 K temperature for 2 h. After activation, the

samples were cooled down to 110 K under dynamic vacuum conditions followed by CO dosing at stepwise increasing pressures. The IR spectra were recorded in transmission after each CO dosage.

Catalysis

Catalytic tests were carried out in a stainless steel autoclave reactor hosting a PTFE liner vessel of 20 mL volume, equipped with a K-type thermocouple and a Jumo dTRANS P30 pressure transducer for continuous digital temperature and pressure monitoring, and mounted on an aluminum heating block on a Heidolph MR 3002 stirring plate. All reactions were carried out solvent-free except from tests where styrene was employed as substrate and dry toluene was used as solvent. The solid catalyst and the reactants were added into the reactor. In all cases, equimolar amounts of the alkene and silane reactants were applied. Next, the system was first purged with N_2 (99.999% purity), the pressure adjusted to 10 bar at room temperature and the reaction initiated by increasing the temperature to the preset reaction temperature at a heating rate of 2 K min^{-1}. The liquid reaction medium was sampled at preset time intervals through a capillary equipped with a porous frit with 2 µm pore opening. Reaction products were identified by gas chromatography in a ISQ chromatograph coupled to a Quadrupole Mass Spectrometer and a Q-Exactive GC Orbi trap (Thermo Scientific). The progress of the reaction was monitored using Nuclear Magnetic Resonance (NMR) Spectroscopy and gas chromatography (GC). Liquid-phase NMR spectra were recorded using Bruker AV-300 and 500 MHz spectrometers. Spectra were referenced to residual proton signals of deuterated solvents. Chemical shifts are indicated in parts per million downfield from tetramethylsilane (TMS). GC analyses were carried out in a Shimadzu QP2010 Plus gas chromatograph, equipped with a ZB-1HT Inferno column (30 m, 0.25 mm ID, df 0.25 µm) and an Flame Ionization Detector (FID) using N_2 as carrier gas. The sample injector temperature was kept at 623 K. Mesitylene was employed as internal standard for quantification. Under selected reaction settings, *hot filtration* tests were performed in order to assess the potential contribution from homogeneous catalysis by metal species leached in

solution. To this end, the liquid reaction phase was collected under reaction conditions using a 100 μL stainless steel capillary immersed in the PTFE liner and equipped with a porous filter frit with a 2 μm opening, once an olefin conversion of ca. 30-40% had been achieved. The hot-filtered liquor was then used as substrate for another reaction batch without further addition of catalyst. To test the recyclability of selected catalysts, up to five consecutive catalytic runs were performed. After each run, the reaction mixture was let cooled down to RT, the solid catalyst recovered by ultracentrifugation, dried in the oven at 423 K for 12 hours and used in the next catalytic test without any further rejuvenation/regeneration treatment. The following equations give the definitions applied herein for conversion, selectivity and yield.

$$Conversion, X = \frac{A\ [mmol\ of\ olefin\ (initial)] - B\ [mmol\ of\ olefin\ (at\ time\ t)]}{A\ [mmol\ of\ olefin\ (initial)]} \cdot 100\ (\%)$$

$$Selectivity\ to\ product\ p, S_p = \frac{mmol\ of\ \ product\ P}{\sum_j\ mmol\ of\ product\ j} \cdot 100\ (\%)$$

$$Yield\ of\ product\ p, Y_p = \frac{mmol\ of\ product\ p\ (at\ time\ t)}{max.stoichiom.mmol\ of\ product\ p} \cdot 100\ (\%)$$

2. Computational details

First-principles Density Functional Theory (DFT) computations were carried out in order to gain molecular insight into the reaction specificity observed with Ru_1/CeO_2 and Rh_1/CeO_2 in the tandem olefin isomerization/hydrosilylation process. The DFT calculations were performed with the plane-wave code VASP version 5.4.1 and the standard VASP-PAW potentials describing the core electrons of the ions.[5] The 1x3 unit cell of the CeO_2(211) slab model with four layers was derived from the CeO_2 bulk structure optimized with the PBE-D3/800eV approach (Perdew-Burke-Ernzerhoff)[6] using a Γ-centered k-point grid (4,4,4) and Gaussian smearing with a width of 0.1 eV.[7] Also, the DFT+U method was employed using a U parameter of 5 eV for cerium 4f-electrons. The size of the unit cell was chosen such that periodic images of adsorbed complexes do not significantly interact. The slab model is depicted in Figure S19. Rh and Ru single atoms were placed at the step-edge corresponding to the stoichiometric type II in Kozlov et al.[8] A linear oxygen coordination is observed for the bare single metal atoms, similar to Pt reported by Dvorak et al.[9] Computed electronic energies are provided in Table S6. Structural data is appended in section 6, at the end of this *Supplementary Information* file. Transition states were located using the ARPESS algorithm.[10] Thermal contributions were evaluated at 393 K and a reference pressure of 10 bar using the rigid-rotator harmonic-oscillator approximation. Calculation were performed with propene and Me_3SiH as model reactants and did not consider solvation effects in the liquid phase. Given the non-polarity of the reaction media employed experimentally (solvent-free, or dry toluene as solvent, see *Catalysis* subsection above), electrostatic interactions with the solvent are expected to be weak.

3. Supplementary Figures

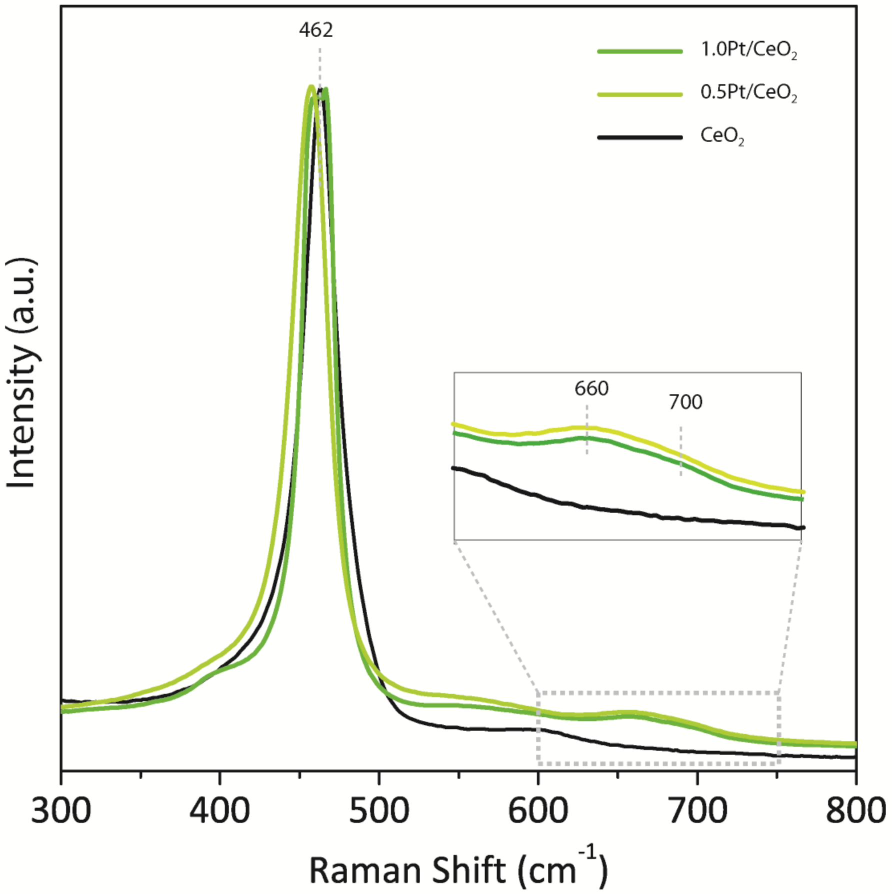

Figure S1: Raman spectra for CeO_2 and selected Pt/CeO_2 catalysts with various metal contents obtained by oxidative redispersion at 1073 K. Spectra have been normalized to the Raman signal for the triply degenerate F2g mode of the CeO_2 lattice (band at ca. 462 cm^{-1}).

The Raman spectrum for pure CeO_2 shows a prominent band at a Raman shift of 462 cm^{-1}, which corresponds to the triply degenerate *F2g* mode of the fluorite-type CeO_2 lattice. A notably weaker and broader band at ca. 590-600 cm^{-1} can be ascribed to Frenkel-type oxygen vacancies in CeO_2.[11] On incorporation of Pt (at metal contents ≤1.0 Pt_{at} nm^{-2}), a broad signal developed, which can be deconvoluted into two bands peaking at 660 and 700 cm^{-1}, respectively. In this region, active Raman modes for Pt-O-Ce are to be expected.[12] Hence the Raman results provide evidence for the creation of Pt-O-Ce linkages upon annealing.

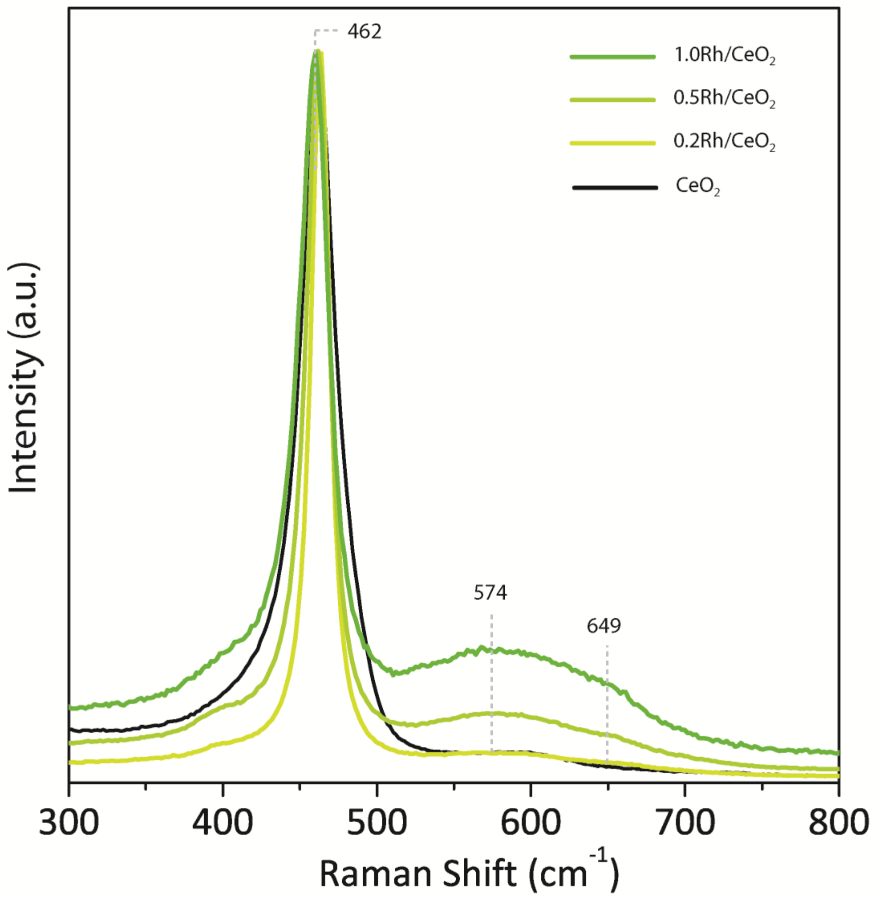

Figure S2: Raman spectra for CeO_2 and selected Rh/CeO_2 catalysts with various Rh contents obtained by oxidative redispersion at 1073 K. Spectra have been normalized to the Raman signal for the triply degenerate F2g mode of the CeO_2 lattice (band at ca. 462 cm^{-1}).

The Raman spectrum for pure CeO_2 shows a prominent band at a Raman shift of 462 cm^{-1}, which corresponds to the triply degenerate *F2g* mode of the fluorite-type CeO_2 lattice. A notably weaker and broader band at ca. 590-600 cm^{-1} can be ascribed to Frenkel-type oxygen vacancies in CeO_2.[11] On incorporation of Rh (at metal contents ≤1.0 Rh_{at} nm^{-2}), a broad signal developed, which can be deconvoluted into two bands peaking at 574 and 649 cm^{-1}, respectively, i.e. the spectral region for Raman active modes associated to Rh-O-Ce species.[13] Hence the Raman results provide evidence for the creation of Rh-O-Ce linkages upon annealing.

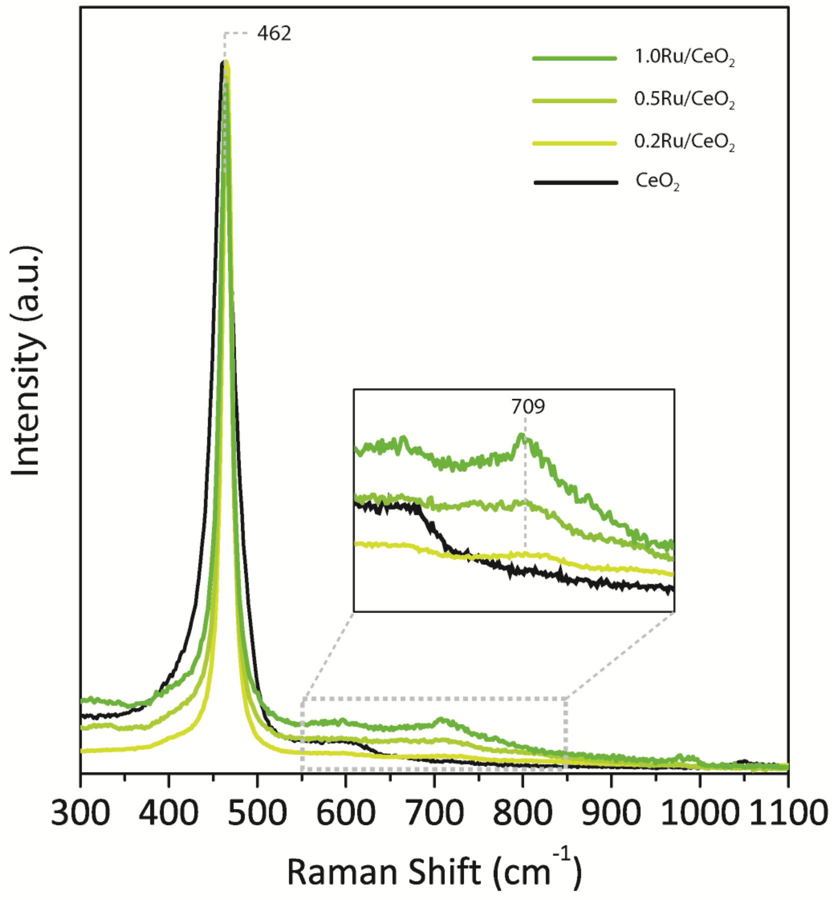

Figure S3: Raman spectra for CeO_2 and selected Ru/CeO_2 catalysts with various Ru contents obtained by oxidative redispersion at 1073 K. Spectra have been normalized to the Raman signal for the triply degenerate F2g mode of the CeO_2 lattice (band at ca. 462 cm^{-1}).

The Raman spectrum for pure CeO_2 shows a prominent band at a Raman shift of 462 cm^{-1}, which corresponds to the triply degenerate *F2g* mode of the fluorite-type CeO_2 lattice. A notably weaker and broader band at ca. 590-600 cm^{-1} can be ascribed to Frenkel-type oxygen vacancies.[11] On incorporation of Ru (at metal contents ≤1.0 Ru_{at} nm^{-2}), a weak band developed at ca. 709 cm^{-1} which can be ascribed to Ru-O-Ce species.[14] Hence, the Raman results provide evidence for the creation of Ru-O-Ce linkages upon annealing.

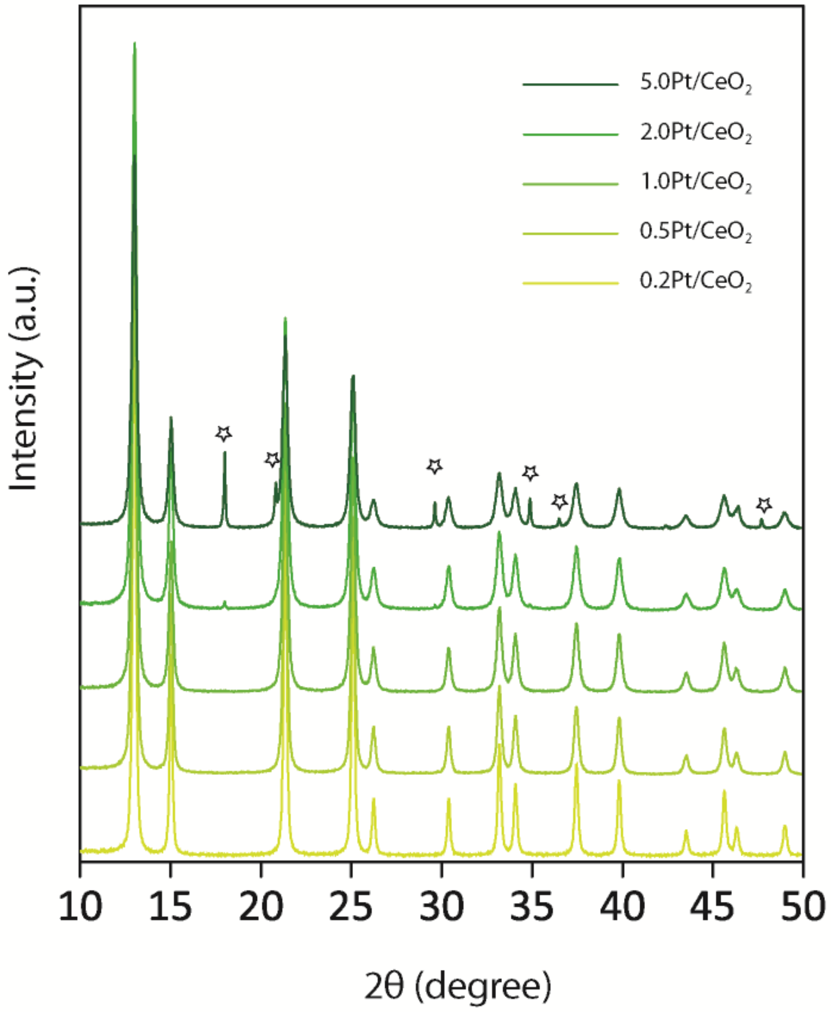

Figure S4: X-ray diffractograms (MoK$_{\alpha1}$ radiation) for a series of Pt/CeO_2 catalysts, with various Pt surface coverages, obtained by oxidative redispersion at 1073 K. Star labels denote diffractions corresponding to metallic Pt (ICDD: 00-004-0802).

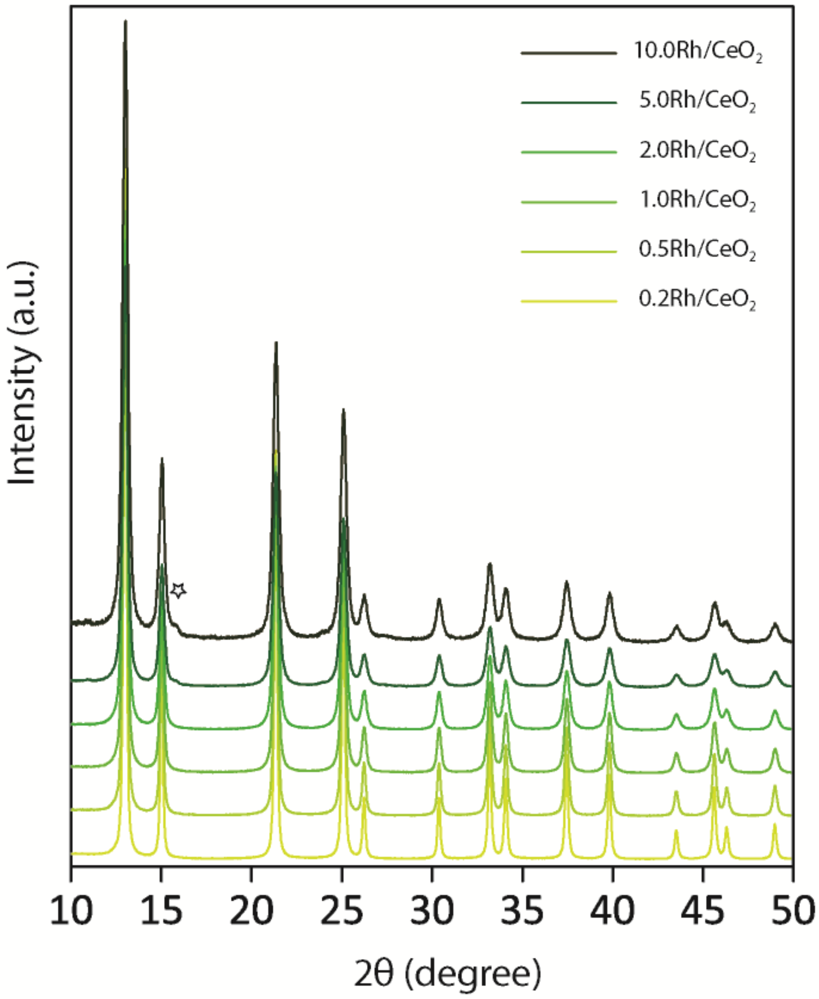

Figure S5: X-ray diffractograms (Mo$K_{\alpha 1}$ radiation) for a series of Rh/CeO_2 catalysts, with various Rh surface coverages, obtained by oxidative redispersion at 1073 K. Star labels denote diffractions corresponding to Rh_2O_3 (ICDD: 00-041-0541).

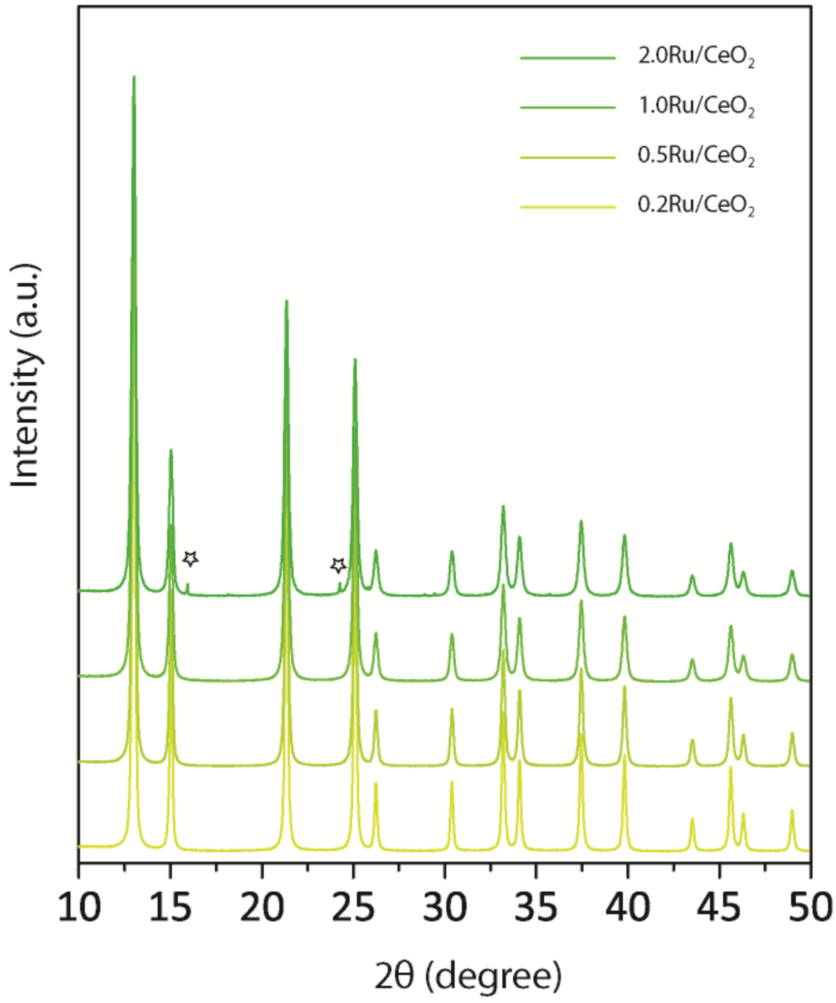

Figure S6: X-ray diffractograms ($MoK_{\alpha 1}$ radiation) for a series of Ru/CeO_2 catalysts, with various Ru surface coverages, obtained by oxidative redispersion at 1073 K. Star labels denote diffractions corresponding to RuO_2 (ICDD: 01-088-0286).

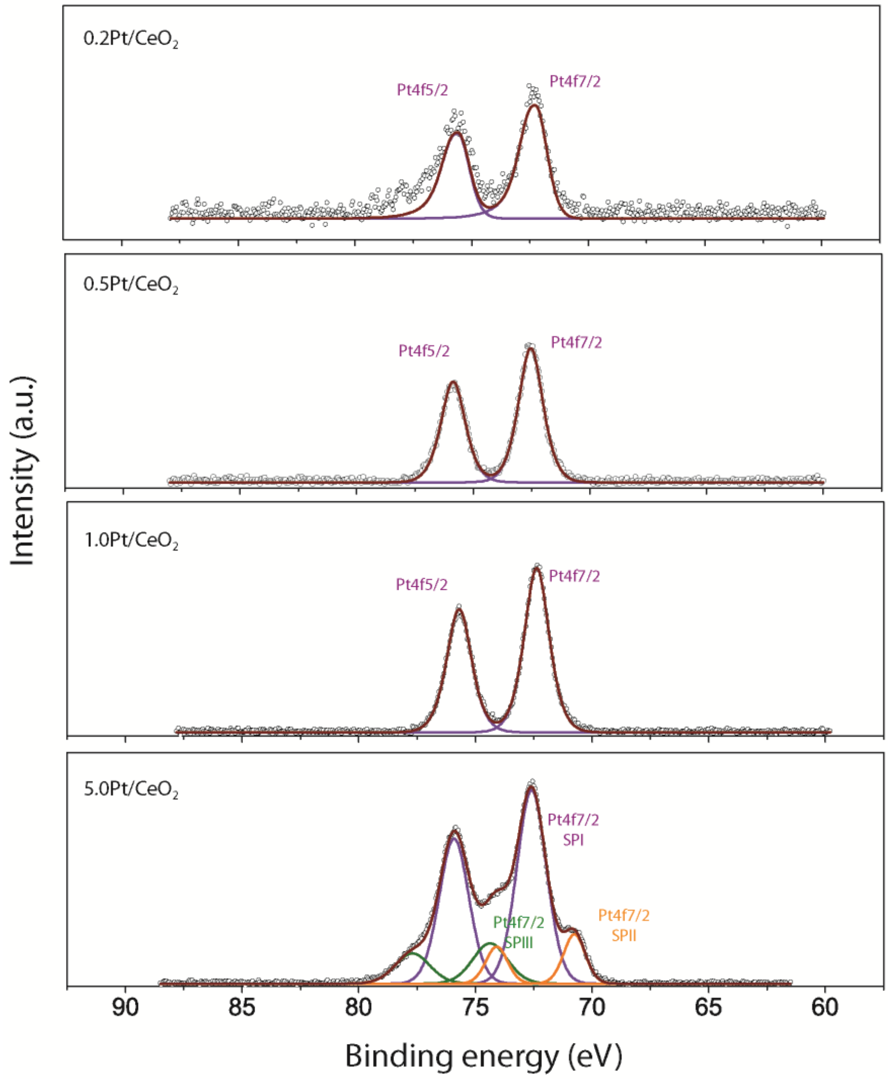

Figure S7: X-ray photoemission spectra in the Pt4f spectra region for a series of Pt/CeO_2 catalysts, with various Pt surface coverages, obtained by oxidative redispersion at 1073 K. The table below summarizes the assignment of different Pt4f binding energies to different platinum species.

Species	Pt4f$_{7/2}$ BE (eV)	Assignment
SPI	72.6	Pt(II)
SPII	70.8	Pt(0)
SPIII	74.4	Pt(IV)

For Pt/CeO_2 catalysts, a Pt4f$_{7/2}$ binding energy (BE) of 72.6 eV was determined for metal coverages ≤2 Pt nm^{-2}, indicating the presence of Pt(II) as the single species. Increasing the Pt content to 5.0 Pt nm^{-2} led to the detection of additional contributions at BEs of 74.4 eV and 70.8 eV, corresponding to Pt(IV) oxide species and metallic Pt(0), respectively. The fact that no diffraction peaks were detected for PtO_2 by XRD at any metal content suggests that these PtO_2 species are either amorphous or they exhibit very small crystalline domains (<2 nm).

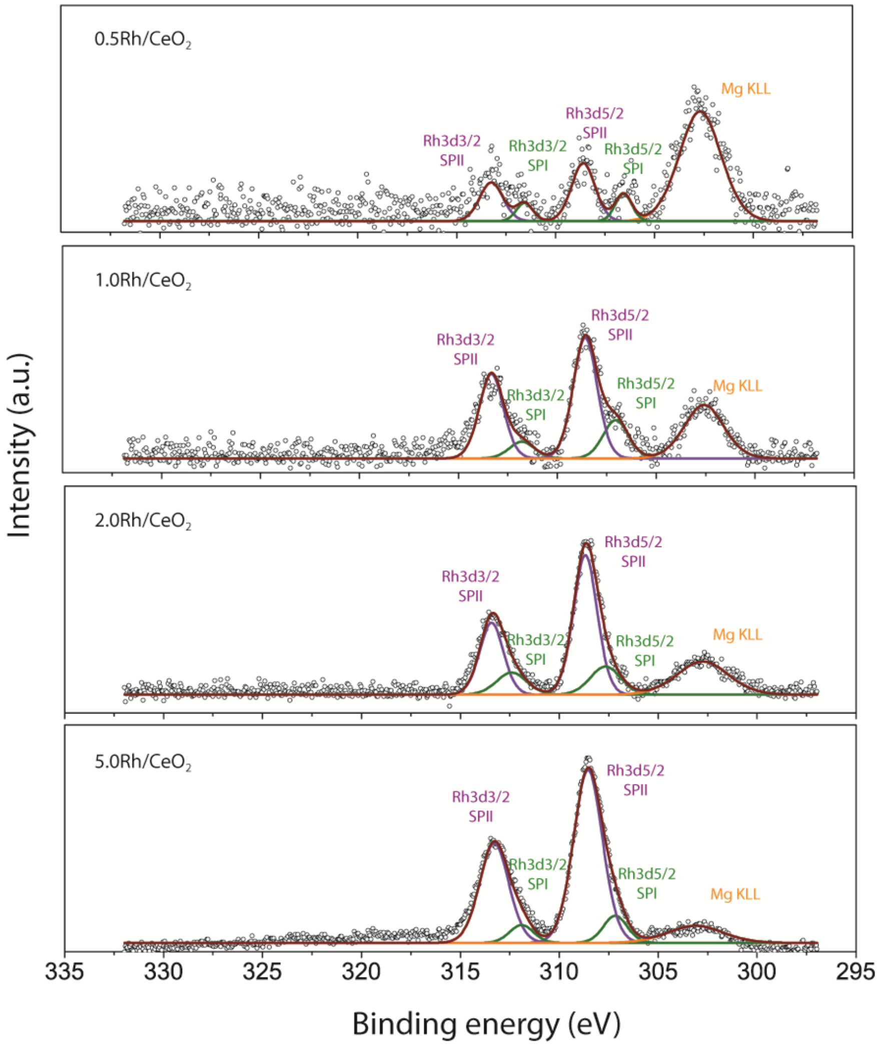

Figure S8: X-ray photoemission spectra in the Rh3d spectra region for a series of Rh/CeO_2 catalysts, with various Rh surface coverages, obtained by oxidative redispersion at 1073 K. The table below summarizes the assignment of different Rh3d binding energies to different rhodium species.

Species	$Rh3d_{5/2}$ BE (eV)	Assignment
SPI	307.2	Rh(0)[a]
SPII	308.5	Rh(III)

[a]Additonal experiments, showed this contrbution to be an artifact, as Rh was reduced *in situ* un der the UHV of the spectrometer chamber (see text below).

For Rh/CeO_2 catalysts, $Rh3d_{5/2}$ contributions with BEs of 308.6 eV and 307.7 eV, respectively, were determined at all studied metal loadings (0.5-5.0 Rh_{at} nm^{-2}). The former species can be assigned to Rh(III), whereas the latter corresponds to metallic Rh(0). However, additional experiments showed the contribution of the latter species to vary with the experimental settings. Hence, the development of metallic Rh was inferred to occur by metal reduction under X-ray irradiation in the UHV conditions of the spectrometer chamber. Independent XANES experiments (see Figure S10) confirmed cationic Rh as the solely metal species in all Rh/CeO_2 catalysts.

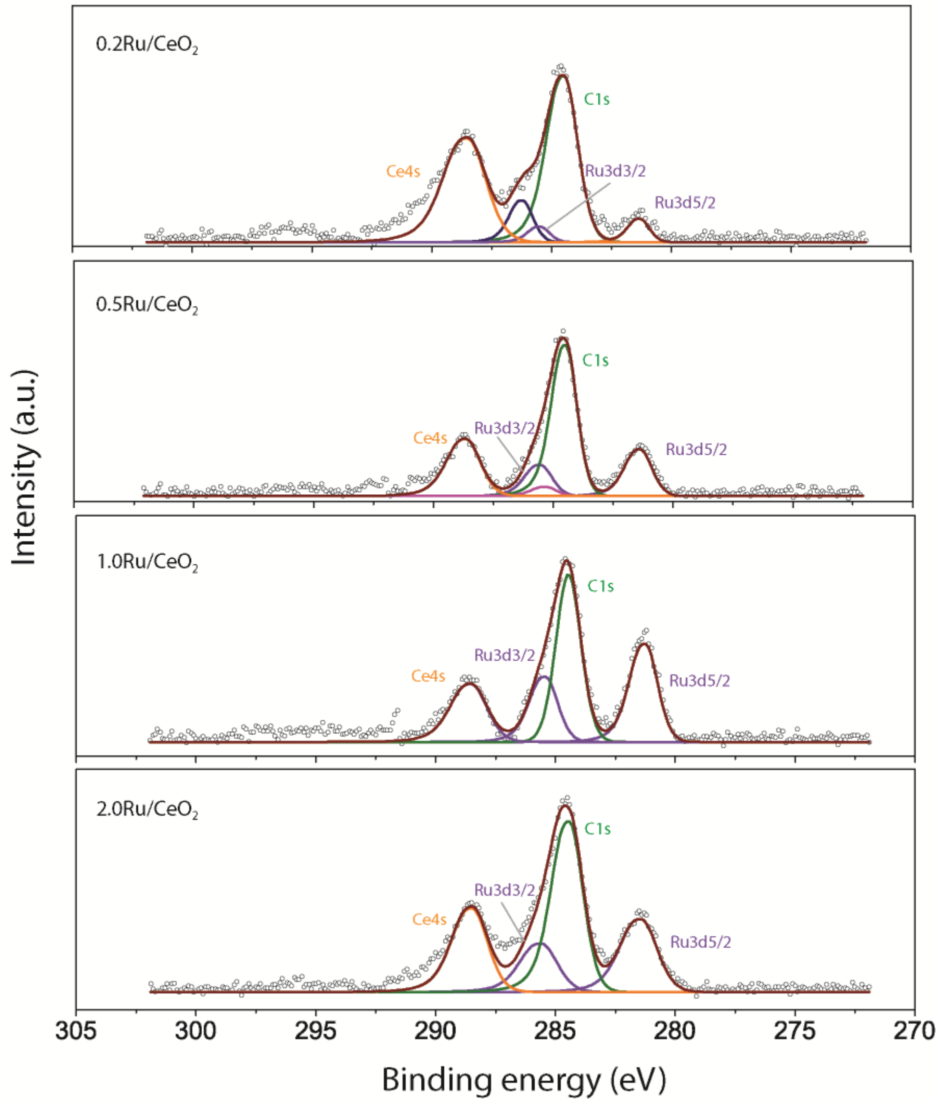

Figure S9: X-ray photoemission spectra in the Ru3d spectra region for a series of Ru/CeO_2 catalysts, with various Ru surface coverages, obtained by oxidative redispersion at 1073 K. The table below summarizes the assignment of the single Ru3d BE contribution to Ru species. Signals ascribed to C1s and Ce4s components are visible in the same region.

Species	$Ru3d_{5/2}$ BE (eV)	Assignment
SPI	281.4	Ru(IV)

For Ru/CeO_2 catalysts, only Ru(IV) species, identified by a $Ru3d_{5/2}$ BE of 281.4 eV, were detected at all metal contents investigated (up to 2.0 Ru_{at} nm^{-2}).

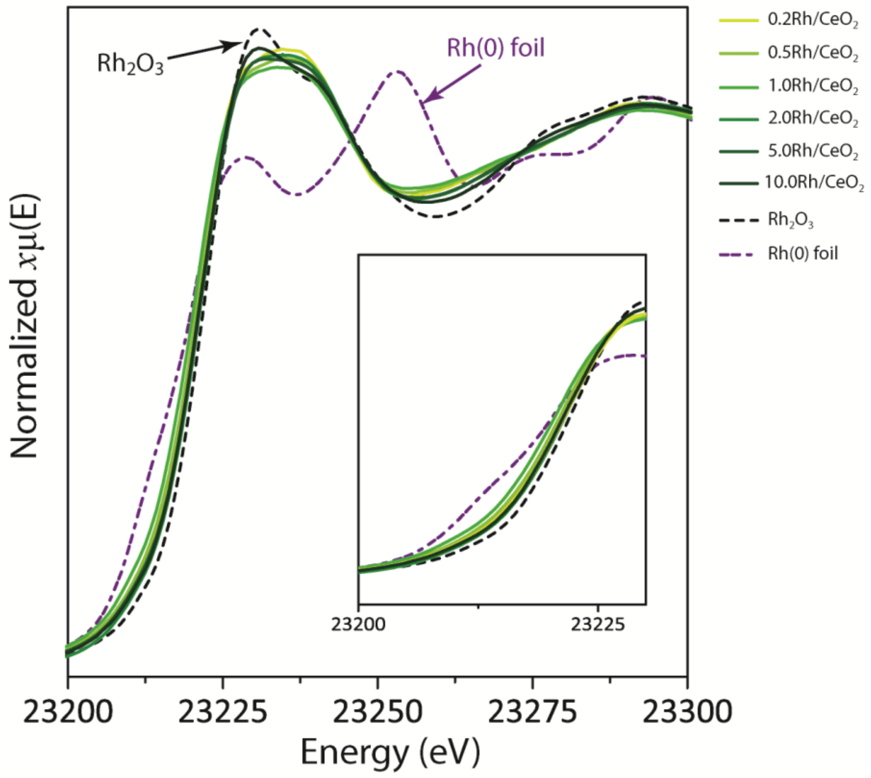

Figure S10: Normalized XANES spectra for as-synthesized Rh/CeO_2 catalysts with various surface-specific Rh contents (0.2-10 Rh_{at} nm^{-2}), along with the corresponding spectra for bulk Rh_2O_3 and Rh(0) foil references. The inset shows an enlargement of the absorption edge region.

The XANES spectra for the entire series of Rh/CeO_2 catalysts was consistent with the presence of exclusively oxidic Rh species. No signs for metallic Rh(0) could be detected. These results verified that the contribution from metallic Rh(0) detected in the XPS spectra for the same samples was an artifact due to the in situ reduction of rhodium species under the UHV conditions of the XPS chamber.

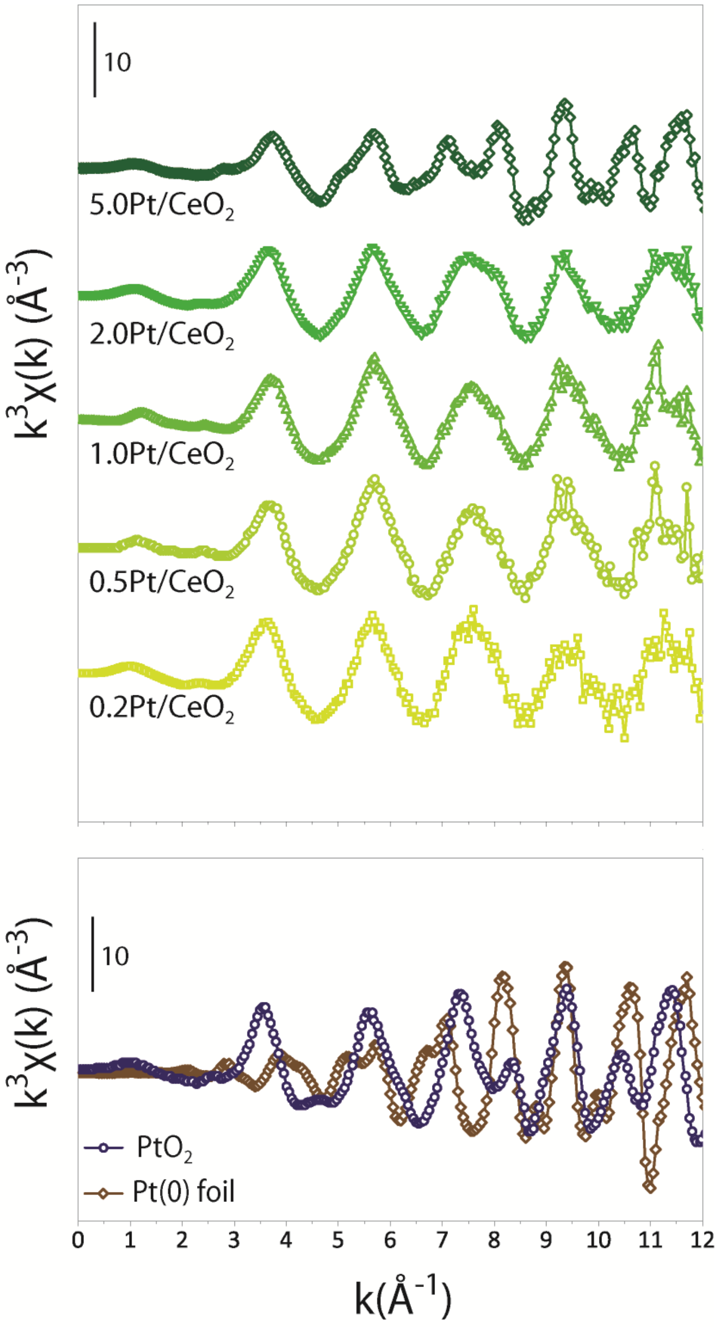

Figure S11: k^3-weighted phase-uncorrected χ(k) EXAFS function in k-space for) Pt/CeO_2 catalysts with different surface metal contents (M_{at} nm^{-2}). The corresponding spectra for bulk metal oxide and metallic foil have also been included for reference in the bottom panel. The scale-marker along the y-axis is identical for both panels.

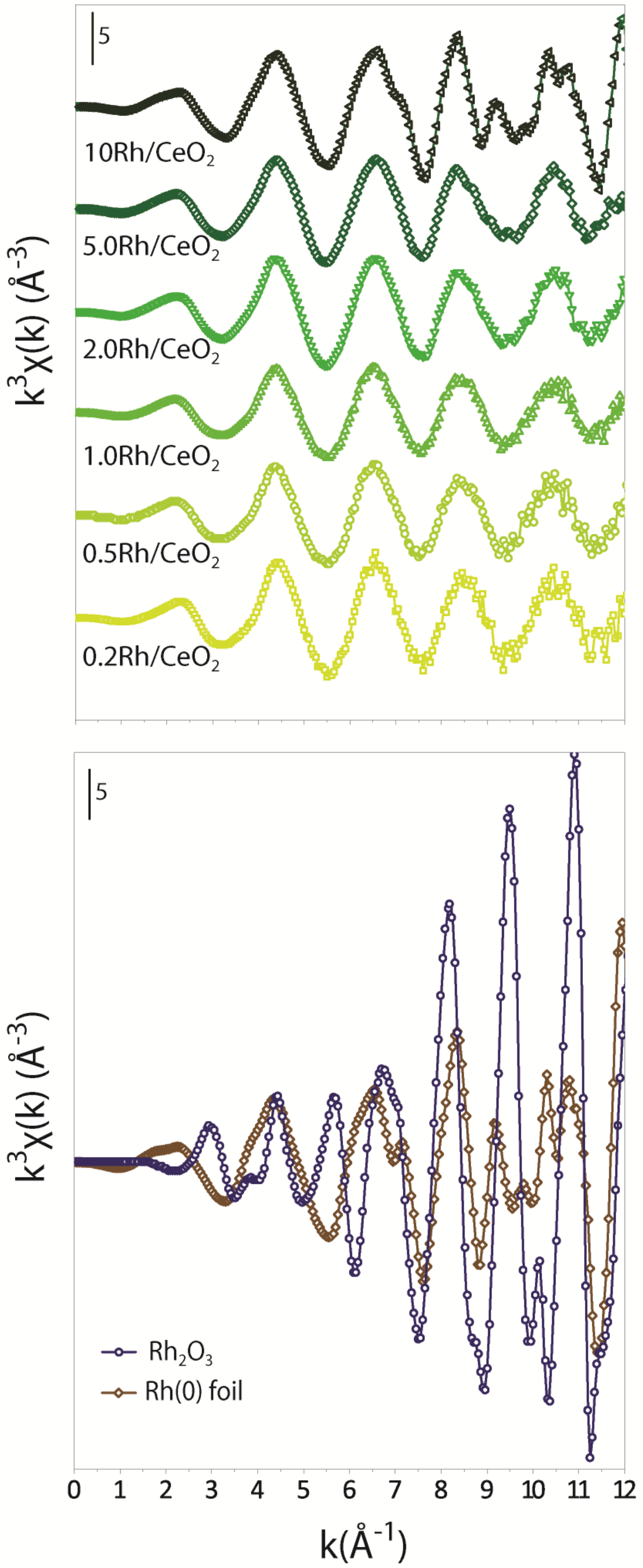

Figure S12: k^3-weighted phase-uncorrected χ(k) EXAFS function in k-space for) Rh/CeO_2 catalysts with different surface metal contents (M_{at} nm^{-2}). The corresponding spectra for bulk metal oxide and metallic foil have also been included for reference. The scale-marker along the y-axis is identical for both panels.

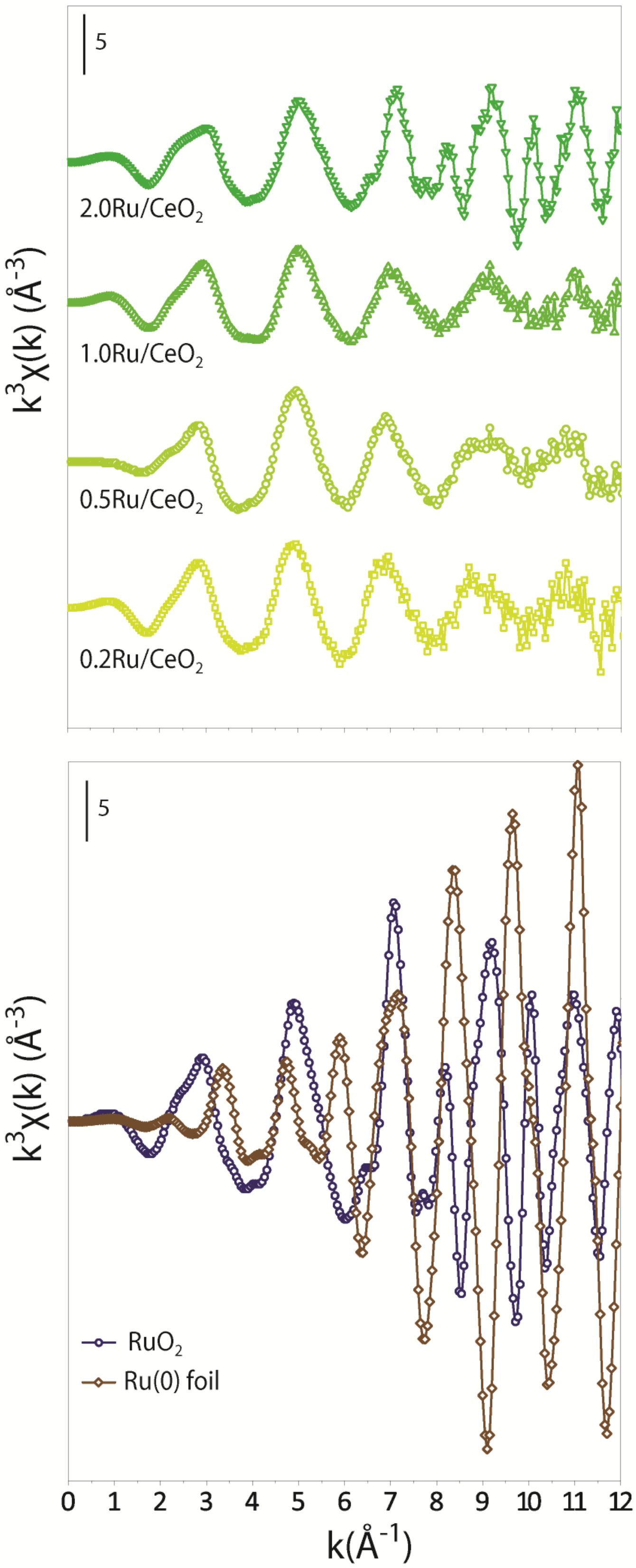

Figure S13: k^3-weighted phase-uncorrected χ(k) EXAFS function in k-space for) Ru/CeO_2 catalysts with different surface metal contents (M_{at} nm^{-2}). The corresponding spectra for bulk metal oxide and metallic foil have also been included for reference. The scale-marker along the y-axis is identical for both panels.

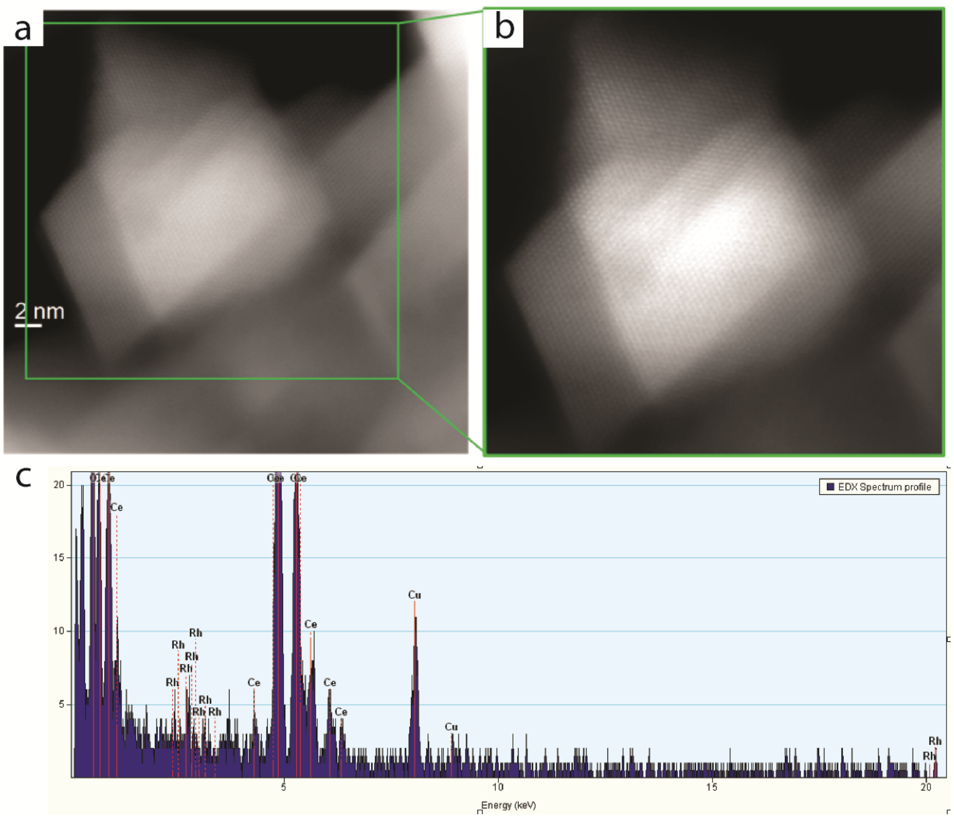

Figure S14: a) Representative high-magnification C_s-HAADF-STEM micrograph for 1.0Rh/CeO_2. b) Atomic-resolution close-up micrograph of the area framed in panel (a). c) EDX spectra collected from the area imaged in (b). Oxygen Kα (0.52 keV), rhodium L (2.5-2.7 keV) and cerium M (ca. 0.9 keV) and L (ca. 4.8-6 keV) lines are identified. Copper signals arise from the TEM grid.

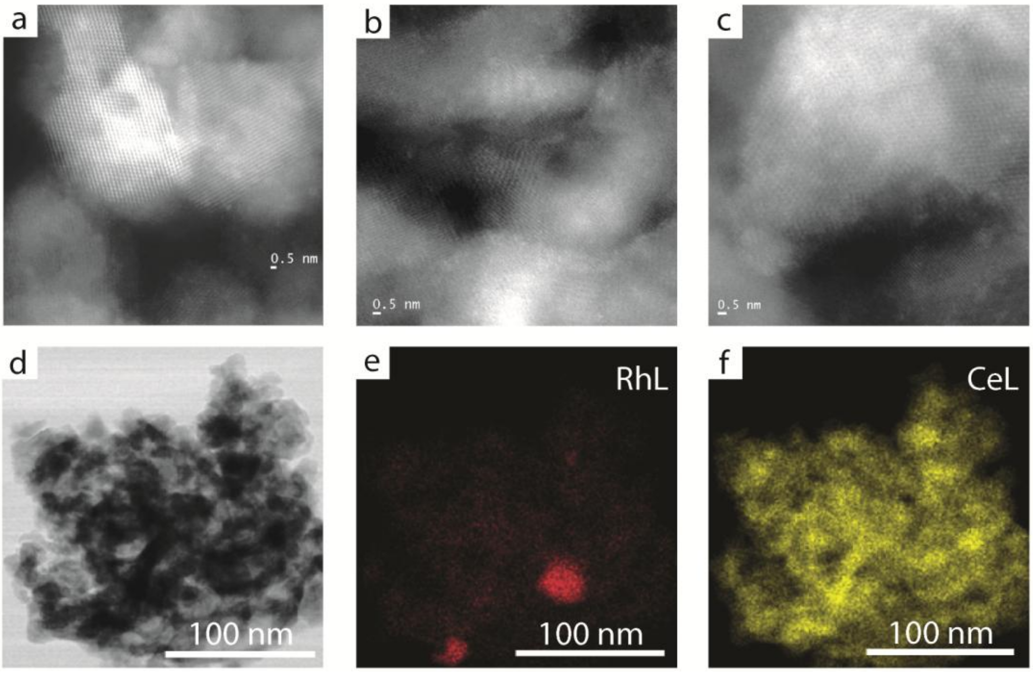

Figure S15: a-c) Representative C_s-HAADF-STEM micrographs for 5.0Pt/CeO_2. d) C_s-HAADF-STEM micrograph and e,f) corresponding EDX compositional maps for Rh and Ce, respectively for 10Rh/CeO_2. In both cases, metal clustering and agglomeration is evident, in line with EXAFS results (see main text). In the case of Pt/CeO_2, the higher Z-contrast of Pt (oxide) species enables the direct visualization of the metal clusters on the CeO_2 surface. In the case of Rh/CeO_2, the limited Z-contrast of Rh_2O_3 requires EDX analysis to identify metal oxide nanoparticles as nanoscale regions of high local Rh concentration (see panel e).

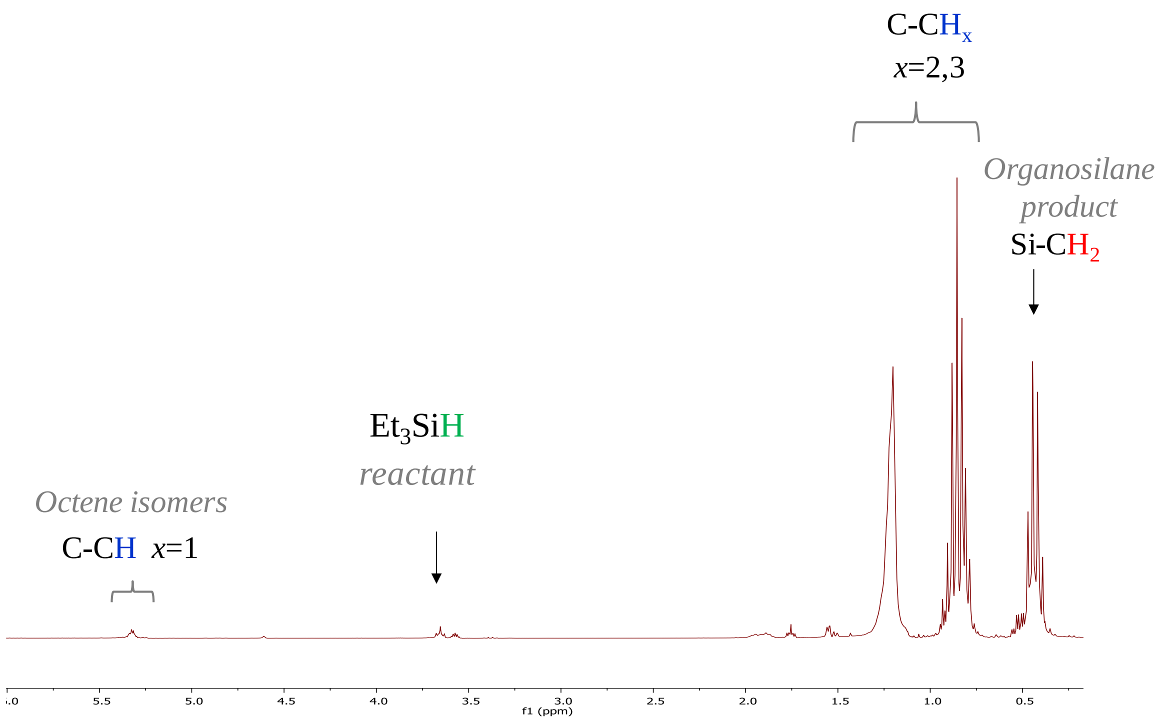

Figure S16: ^{1}H NMR spectrum for the crude product after the hydrosilylation of 1-octene with Et_3SiH using . 1.0Rh/CeO_2 as catalyst. Reaction conditions: 1-octene (5 mmol), triethylsilane (5 mmol), catalyst (2 µmol, metal basis), P=10 bar (N_2, 99.999% purity), reaction time 2 h.

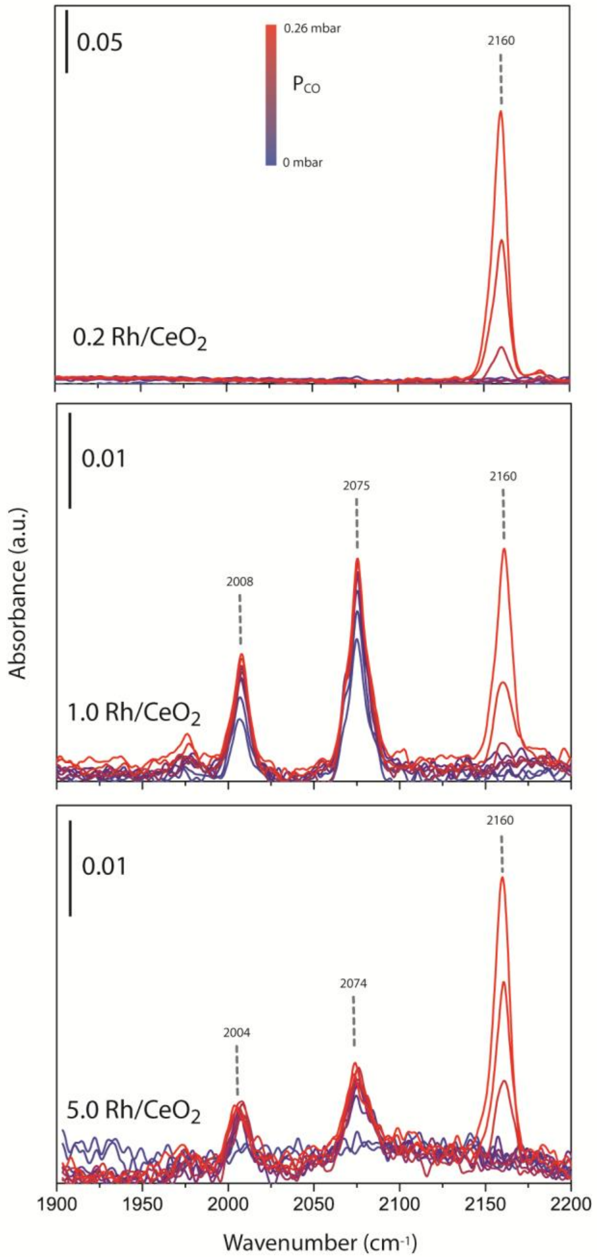

Figure S17: Fourier-Transform Infrared (FTIR) spectra (collected at T=110 K) after adsorption of CO at increasing dosages on the surface of 0.2Rh/CeO_2, 1.0Rh/CeO_2 and 5.0Rh/CeO_2 catalysts. The legend color code for CO partial pressures applies to all panels.

FTIR spectroscopy coupled to CO as surface probe was applied to study the nature of surface Rh species on selected Rh/CeO_2 catalysts. For the catalyst with the lowest metal content of 0.2 Rh_{at} nm$^{-2,}$ only a band peaking at 2160 cm^{-1} emerged upon increasing the CO partial pressure in the cell above 0.11 mbar. This band is ascribed to CO linearly bond to *cus* Ce^{4+} Lewis centers on the CeO_2 surface.[15] No bands in the spectral region 2000-2100 cm^{-1} could be observed, where Rh^{x+}-carbonyl vibrations are expected. This result indicates the absence of accessible Rh atoms on the catalyst surface. On the contrary, very prominent bands peaking at 2008 and 2075 cm^{-1}, respectively, could be observed already from P_{CO}<0.05 mbar in the case of 1.0Rh/CeO_2. These bands are known to correspond to the asymmetric and

symmetric stretching modes, respectively, of $Rh^+(CO)_2$ *gem*-dicarbonyl species and are a fingerprint for atomically dispersed Rh complexes on oxide carriers.[16] The detection of these signals from very low CO partial pressures and their prominent intensity suggest that the single Rh atoms are abundantly and readily accessible on the catalyst surface. At higher CO dosages, the band at 2160 cm^{-1}, corresponding to Ce^{4+} centers on the CeO_2 support emerges. For a catalyst with a higher metal content (5.0Rh/CeO_2), the doublet of bands assigned to $Rh^+(CO)_2$ *gem*-dicarbonyl species become noticeable only after the CO dosage had reached 8-fod that required in the case of 1.0Rh/CeO_2. Moreover, these bands clearly showed a lower relative intensity with respect to the Ce^{4+}-CO band at the same CO dosage level (0.26±0.02 mbar). Although a direct quantification of individual species is not possible, a qualitative comparison of the bands arising from surface Rh atoms and the ceria support suggests a lower density of surface-exposed Rh^{x+} centers in spite of the higher overall Rh content. It is inferred from these results that the metal oxide (RhO_x) agglomerates which develop on the catalyst surface at surface metal contents in excess to 1-2 Rh_{at}/nm^2 (observed by XRD (Figure S5) and EXAFS (Figure 2b)) might also deplete the catalyst surface from atomically dispersed species, possibly via an Ostwald ripening mechanism during the high-temperature catalyst synthesis, compared to catalysts with lower metal contents and no metal agglomerates, e.g. 1.0Rh/CeO_2.

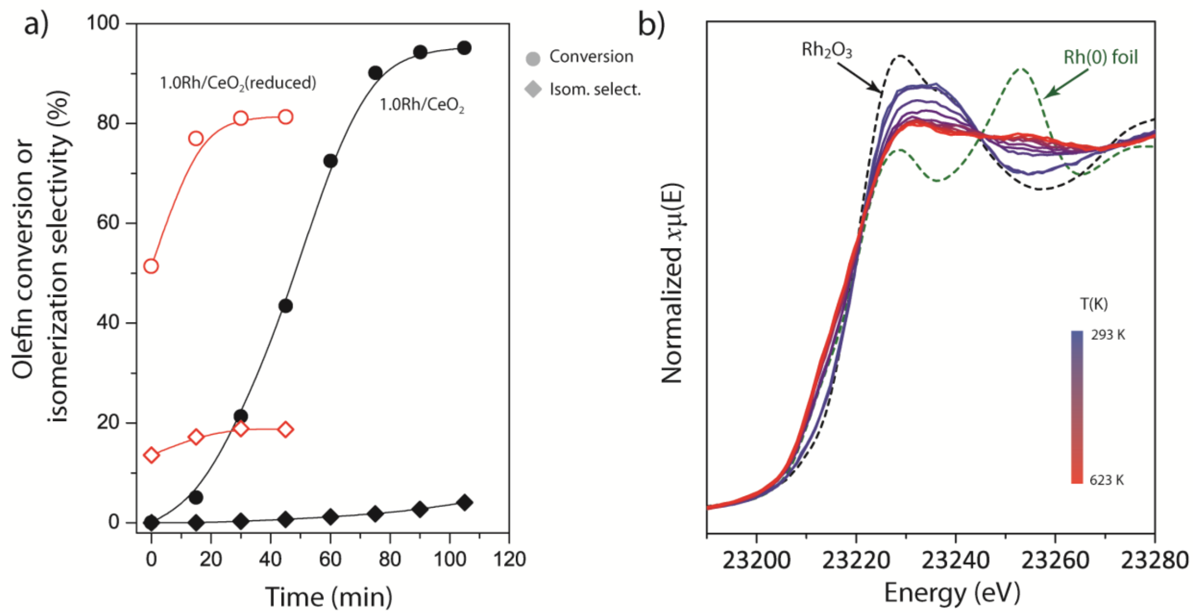

Figure S18: a) Evolution of the olefin conversion (circles) and the selectivity to olefin isomers (diamonds) in the hydrosilylation of 1-octene with Et_3SiH employing as catalyst 1.0Rh/CeO_2 as-prepared (full black symbols) and after a reduction treatment at 623 K in 20%H_2/N_2 (open red symbols). Reaction conditions: 1-octene (5 mmol), triethylsilane (5 mmol), catalyst (2 µmol, metal basis), P=10 bar (N_2, 99.999% purity). b) Evolution of the normalized XANES spectra for the 1.0Rh/CeO_2 catalyst with the temperature during the *in situ* XAS-monitored reduction under flow of 20%H_2/N_2. Reference spectra for bulk-type Rh_2O_3 and metallic Rh(0) foil are also included in the plot (dashed lines). Linear deconvolution of the XANES spectrum after reduction at 623 K, as a linear combination of the spectra for the as-prepared (unreduced catalyst) and the metallic Rh foil, respectively, determined the Rh(0) content in the as-reduced catalyst to be 61%, confirming the partial reduction of rhodium species.

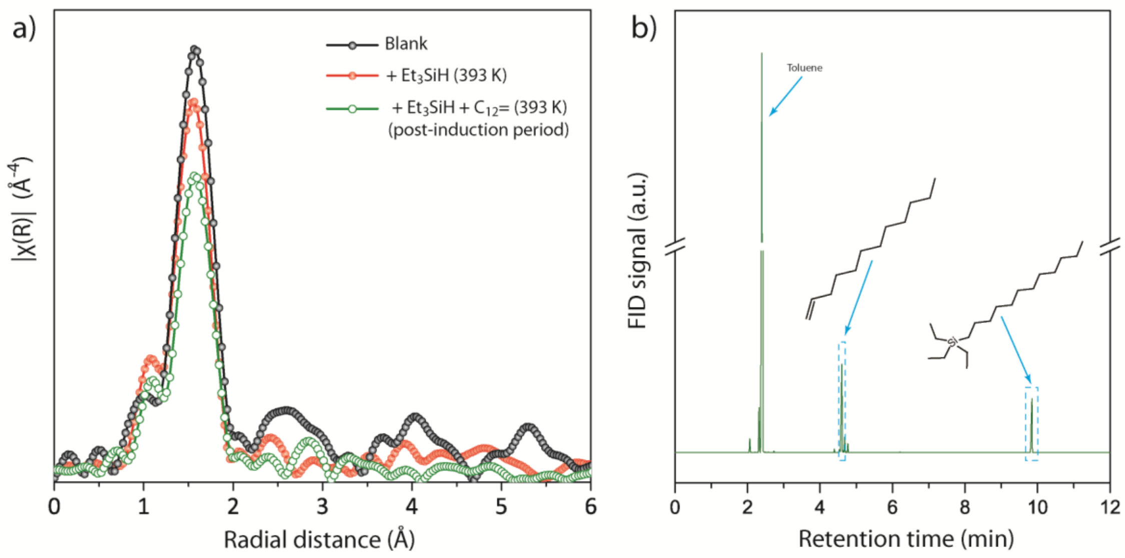

Figure S19: a) |FT| of the k^3-weighted χ(k) EXAFS function (radial distances not phase-corrected) for the 1.0Rh/CeO_2 single-atom catalyst in slurry phase after exposure to the reaction temperature in *n*-dodecane solvent (blank test, full black symbols), in 3 mmol Et_3SiH + 3 mmol *n*-dodecane (full red symbols) and in 3 mmol Et_3SiH + 3 mmol 1-dodecene, hence fully mimicking reaction conditions (open green symbols) for 1 hour. b) Gas chromatrogram showing confirming the partial conversion of 1-dodecene to the corresponding 1,1,1-trimeyl-1-dodecylsilane and hence the completion of the catalysis induction period under the reaction conditions applied.

After exposure of 1.0Rh/CeO_2 to olefin hydrosilylation conditions for a reaction time of 1 hour, sufficient to complete the catalysis induction period, the spectrum showed a decrease in the amplitude for the |FT| of the EXAFS function at the radial distance of ~1.59 Å, corresponding to the first coordination shell around the Rh atoms (Fig. S19a). This is compatible with the cleavage of Rh-O bonds during the induction period and the substitution of oxygen by lighter, e.g. carbon, atoms in the Rh most direct coordination sphere during the development of the hydrosilylation-active metal sites. The spectrum for the catalyst after the catalysis induction period does not reveal any scattering contribution at radial distances >2.4 Å which could be ascribed to either first-shell *Rh*-*Rh* or second shell *Rh*-O-*Rh* scattering contributions in dimeric (or larger agglomerate) rhodium (oxide) species.

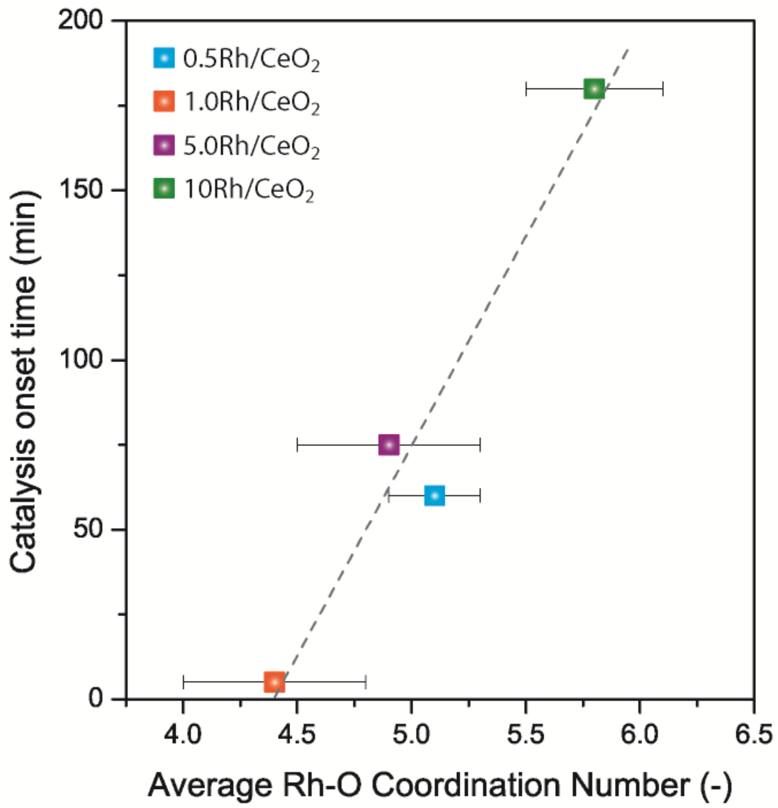

Figure S20: Relationship between the catalysis onset time (duration of the induction period) in the hydrosilylation of 1-octene with Et_3SiH and the average Rh-O coordination number (as derived by fitting of the EXAFS spectra for the as-prepared catalysts) for the series of Rh/CeO_2 catalysts. Reaction conditions: 1-octene (5 mmol), triethylsilane (5 mmol), catalyst (2 µmol, Rh metal basis), P=10 bar (N_2, 99.999% purity), T=393 K. The dotted line is added as a guide to the eye.

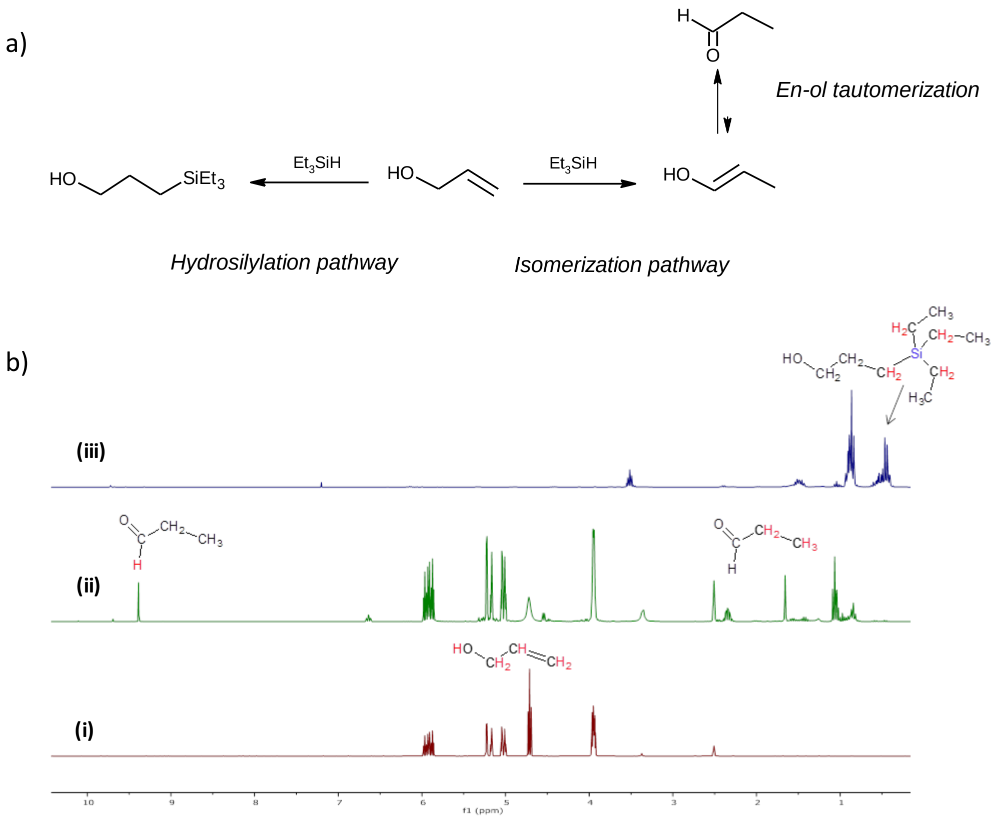

Figure S21: a) Scheme showing the major reaction pathways for allyl alcohol under hydrosilylation conditions. b) (i) ^{1}H NMR spectrum for a) pure allyl alcohol (prop-2-en-1-ol) reactant; (ii) the crude product after the isomerization reaction in the absence of Et_3SiH using 1.0Rh/CeO_2 as catalyst; and (iii) the crude product after the hydrosilylation reaction in the presence of Et_3SiH using 1.0Rh/CeO_2 as catalyst. Reaction conditions: allyl alcohol (10 mmol), triethylsilane (10 mmol), catalyst (4 µmol, metal basis), P=10 bar (N_2, 99.999% purity), reaction time 18 h.

In order to assess whether olefin hydrosilylation or isomerization proceeded faster under reaction conditions on different SACs, 2-propen-1-ol (allyl alcohol) was applied as olefin substrate. In this case, the tautomerization equilibrium established between the double-bond isomerization product, 1-propen-1-ol, and the thermodynamically most stable propanal, provides an energy "sink" which inhibits double-bond back-migration and it thus enables a direct assessment of the relative forward reaction rates for olefin isomerization and hydrosilylation, respectively (**Figure S21a**). As shown in **Table S5**, in the absence of Et_3SiH, 1.0Rh/CeO_2 led to a 7% olefin conversion after 18 h, exclusively via isomerization. In presence of the silane reagent, however, an essentially full conversion of the olefin was attained, with a selectivity >90% to the terminal 1-silyl-propan-2-ol product. These results prove that olefin hydrosilylation proceeds notably (>30 times) faster than isomerization on isolated Rh sites. Platinum sites on 1.0Pt/CeO_2 led to a lower overall olefin conversion (82%) and similar selectivities to both isomerization and hydrosilylation products, evidencing comparable forward reaction rates along both pathways.1.0Ru/CeO_2, albeit essentially inactive in the absence of the Et_3SiH reactant, showed to be very selective for olefin isomerization.

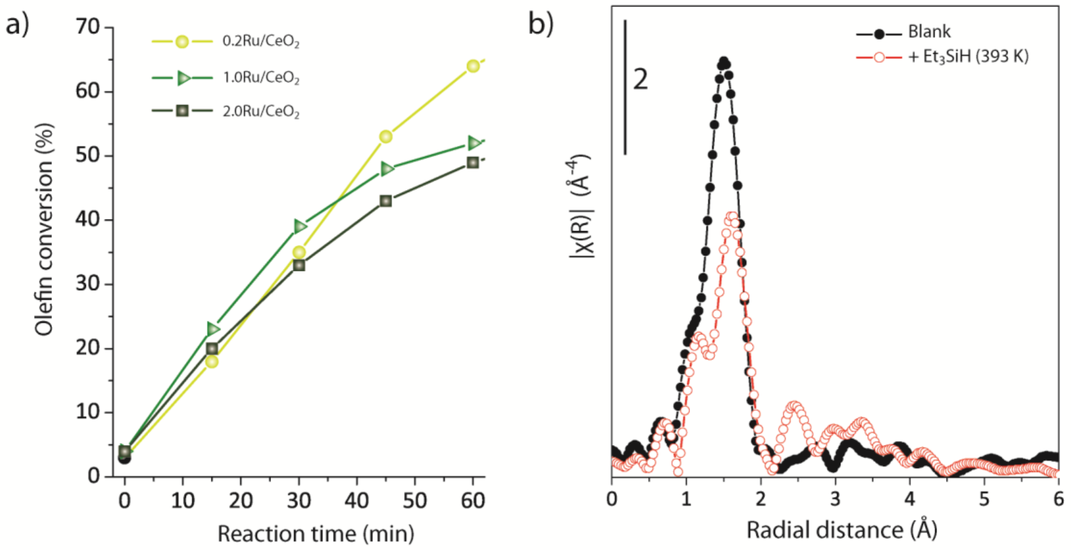

Figure S22: a) Time-resolved evolution of the olefin conversion under reaction conditions for the hydrosilylation of 1-octene with Et_3SiH employing Ru/CeO_2 catalysts synthesized with different surface metal content. For clarity, only data points for selected catalysts of those tested are displayed. Olefin conversion was essentially to isomers. Reaction conditions: 1-octene (5 mmol), triethylsilane (5 mmol), catalyst (2 µmol, Ru metal basis), P=10 bar (N_2, 99.999% purity), T=393 K. b) |FT| of the k^3-weighted χ(k) EXAFS function (radial distances not phase-corrected) for the 1.0Ru/CeO_2 single-atom catalyst after exposure to the reaction temperature in *n*-dodecane solvent (blank test, full symbols) or in an excess of Et_3SiH in *n*-dodecane (open symbols).

No induction period was observed to precede 1-octene conversion (to isomers) with Ru/CeO_2 catalysts (Fig. 22a). This indicates that the development of the isomerization-active Ru species, which takes place only in the presence of Et_3SiH, is kinetically facile. This is in line with the observation of a distortion (decrease in amplitude) for the |FT| of the EXAFS function at the radial distance of ~2.03 Å, corresponding to the first coordination shell around the Ru atoms (Fig. S22b), short after the catalyst is exposed to excess Et_3SiH and brought to reaction temperature (393 K), which is compatible with a fast cleavage of Ru-O bonds and the formation of Ru hydride species *in situ*, which are known to be active sites for olefin isomerization.[17]

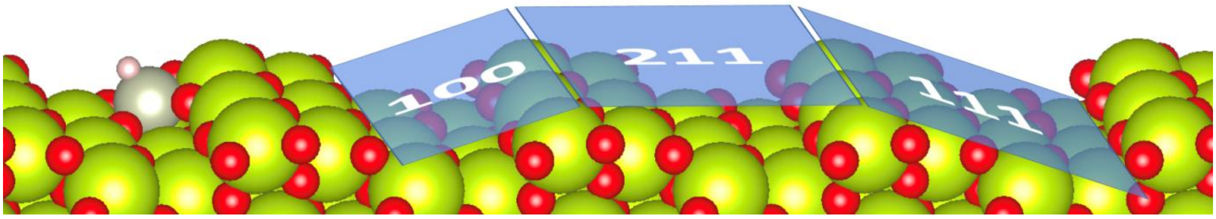

Figure S23: Schematic illustration of the stepped CeO_2 surface considered in the DFT calculations. A 211-surface, that connects two (111) facets has been used to simulate a type II CeO_2 step-edge where single-atom metal centers are stabilized. Atom color code: gray (Rh), pink (H), red (O), green (Ce).

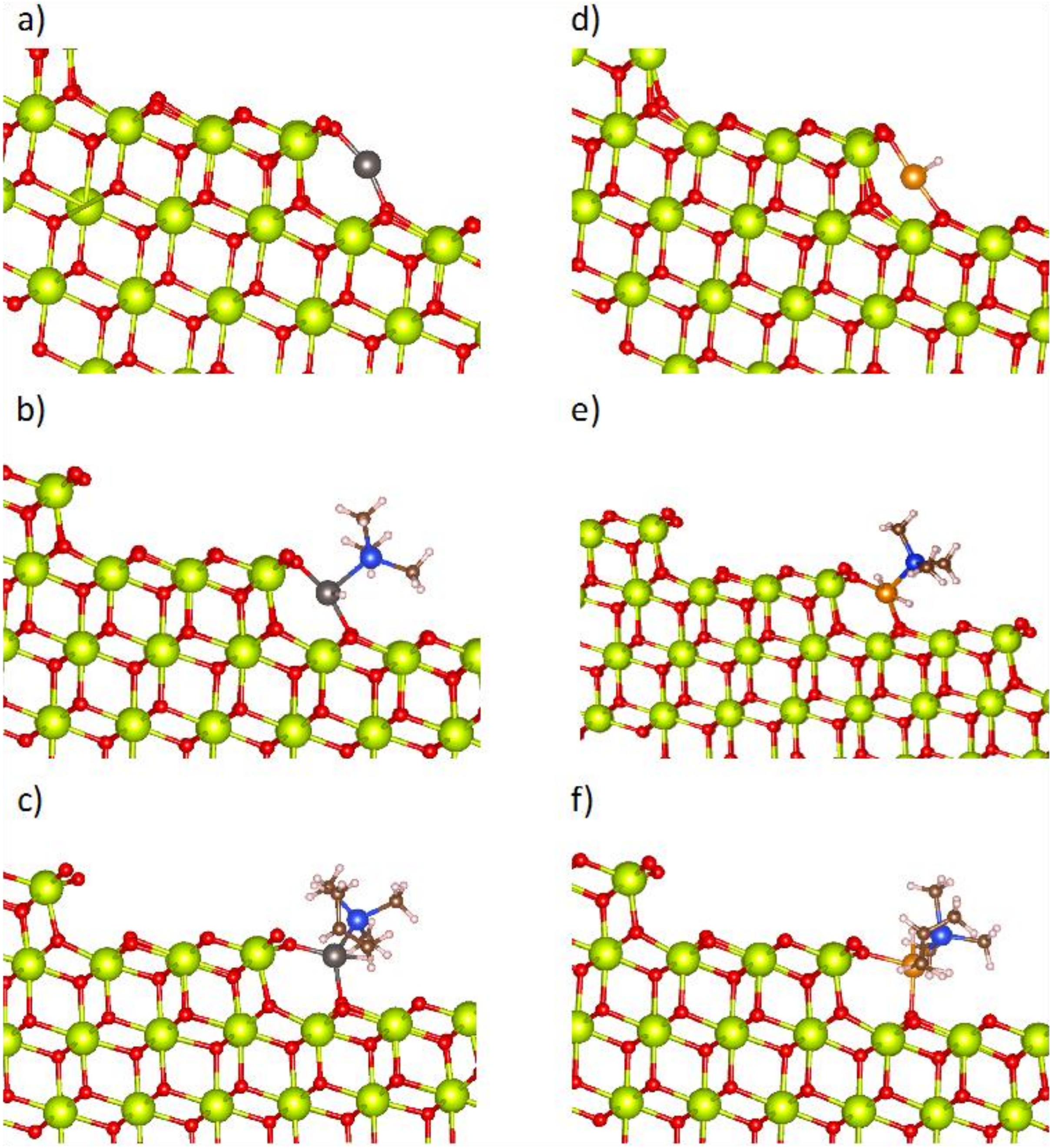

Figure S24: The CeO_2(211) slab was used to model step edges of a corrugated 111-surface. DFT optimized Ru (a-c, gray atom) and Rh (d-e, orange atom) single-atom sites are located at the step edge.

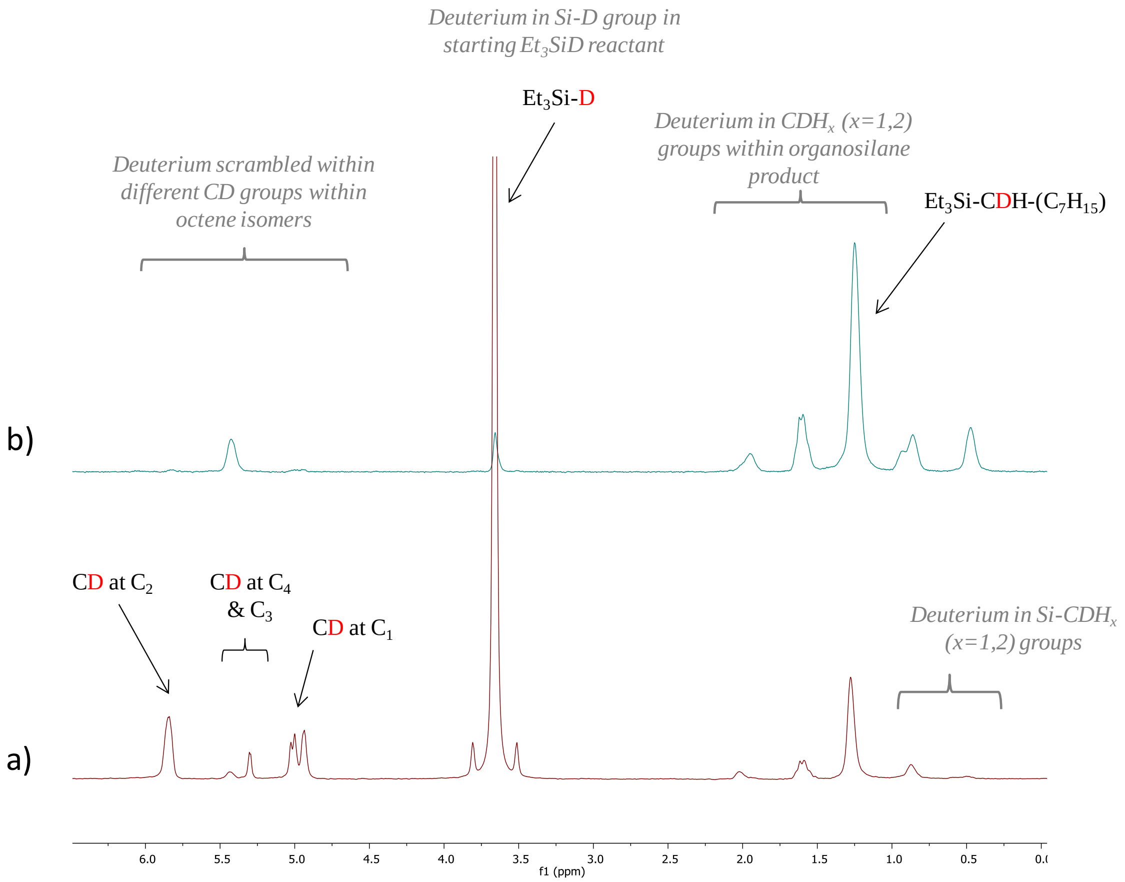

Figure S25: ^2H NMR of the crude reaction mixture after the hydrosilylation of 1-octene with Et_3SiD employing $1.0Rh/CeO_2$ as catalyst after a) 10% and b) 90% silane consumption. Reaction conditions: 1-octene (5 mmol), triethylsilane (5 mmol), catalyst (2 µmol, metal basis), P=10 bar (N_2, 99.999% purity).

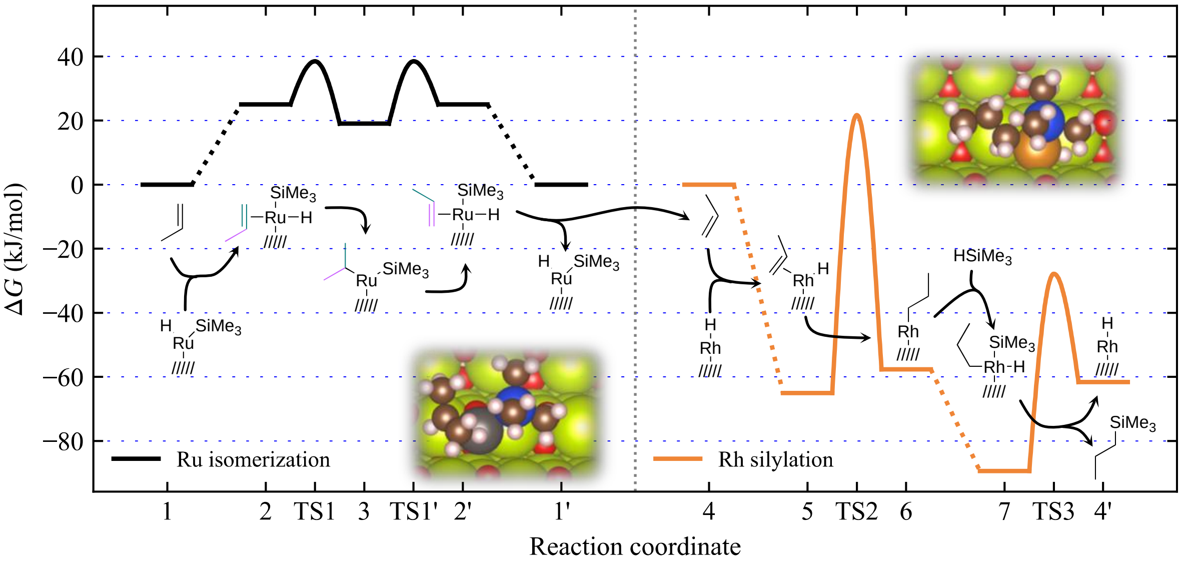

Figure S26: DFT-computed Gibbs free energy diagram for a tandem olefin isomerization/hydrosilylation process with olefin isomerization and olefin hydrosilylation with Me_3SiH catalyzed on $Ru_1/CeO_2(211)$ and $Rh_1/CeO_2(211)$ single-atom centers, respectively. In this specific case, the olefin hydrosilylation reaction pathway is inspired by previously proposed reaction mechanisms.[18] Olefin coordination preceeds oxidative addition of the hydrosilane reagent. Olefin: propene; hydrosilane: Me_3SiH; temperature=393 K, reference pressure=10 bar.

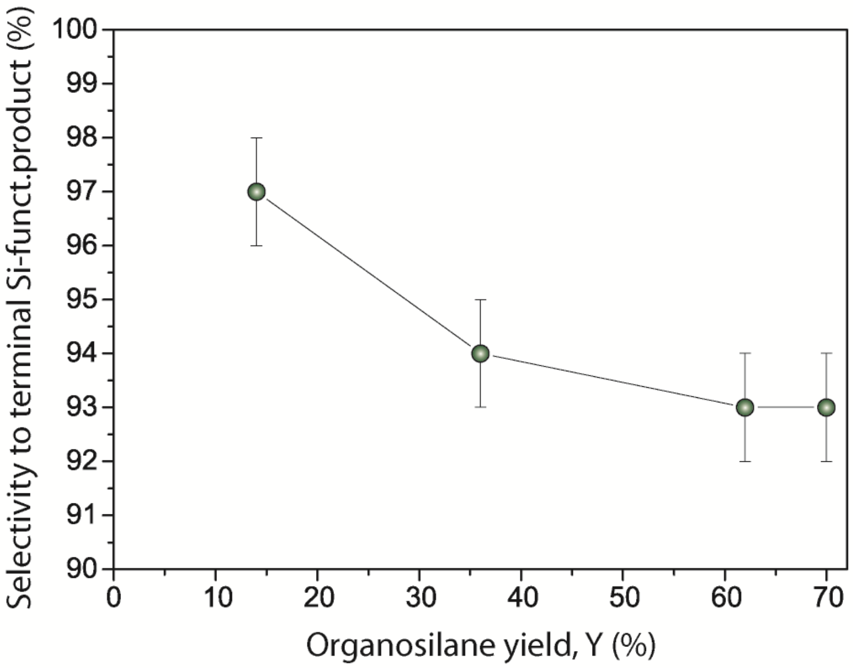

Figure S27: Evolution of the selectivity to linear organosilane product (1,1,1-triethyl-1-octylsilane) with the reaction yield for the tandem isomerization/hydrosilylation of 2-octene with Rh_1/CeO_2 and Ru_1/CeO_2 single-atom catalysts. Reaction conditions: olefin (5 mmol), triethylsilane (5 mmol), catalyst (4 µmol (total metal basis), Ru/Rh=1 (mol)), P=10 bar (N_2, 99.999% purity). Different yields correspond to reaction times of 4, 8, 13 and 18 h.

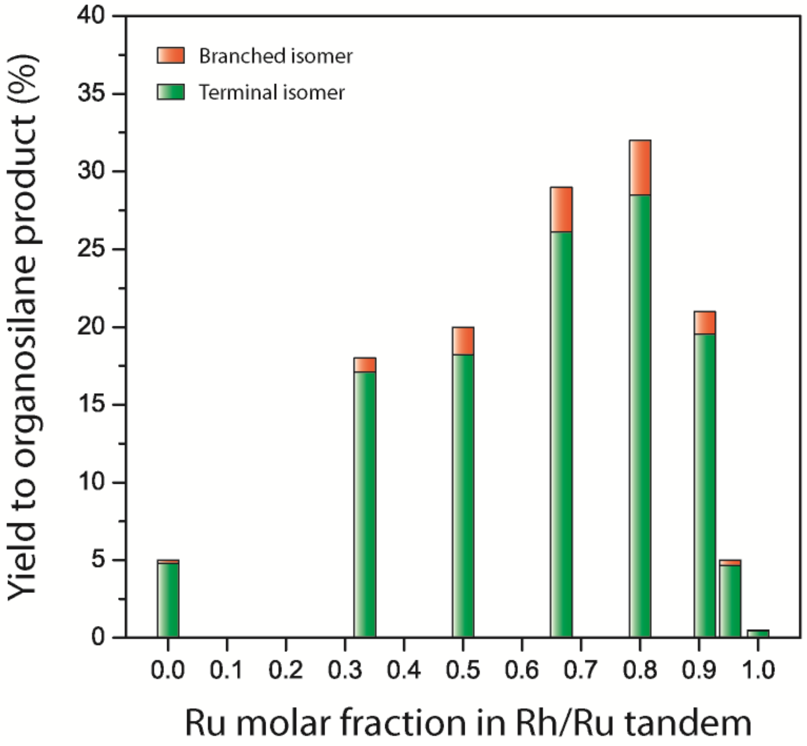

Figure S28: Evolution of the yield to organosilanes (remaining products are olefin isomers) with the Ru/Rh relative abundance in the tandem isomerization-hydrosilylation of 3-octene via single-pot combination of 1.0Rh/CeO_2 and 1.0Ru/CeO_2 single-atom catalysts. Reaction conditions: olefin (5 mmol), triethylsilane (5 mmol), catalyst (4 µmol metal (overall) in all cases), P=10 bar (N_2, 99.999% purity). The linear:branched organosilane ratio is also indicated by the stacked color code in the columns.

As illustrated in Fig. S28, the tandem cooperation of Rh_1/CeO_2 and Ru_1/CeO_2 single-atom catalysts achieves a remarkable performance on an internal olefin such as 3-octene, for which either of the two catalysts individually is barely active. The optimal reactivity is attained for a blend of catalysts with a Ru/Rh ratio of 4 (i.e. 80% Ru in an overall metal basis)..

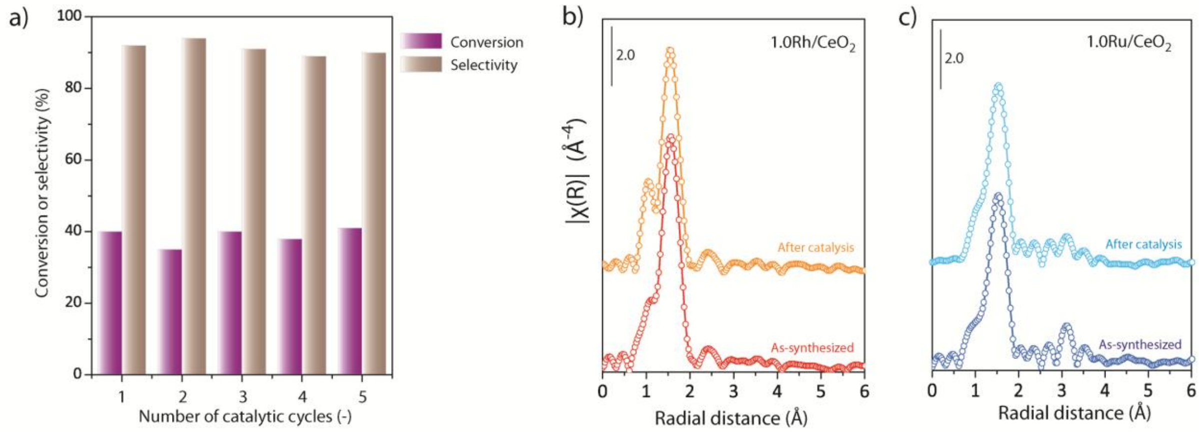

Figure S29: Catalyst stability and recyclability. a) Olefin conversion and selectivity to 1,1,1-triethyl-1-octylsilane in five consecutive catalytic runs in a tandem 2-octene isomerization-hydrosilylation process via the single-pot integration of 1.0Rh/CeO_2 and 1.0Ru/CeO_2 single-atom catalysts. Reaction conditions: 2-octene (5 mmol), triethylsilane (5 mmol), catalyst (2 µmol Rh, 2 µmol Ru), P=10 bar (N_2, 99.999% purity). |FT| of the k^3-weighted χ(k) EXAFS function in R-space at the Rh K-edge (b) and Ru K-edge (c) for the blend of solid catalysts recovered after the tandem olefin isomerization-hydrosilylation run. For comparison, the EXAFS spectra for the pristine (as-synthesized) catalysts are also included in the plot.

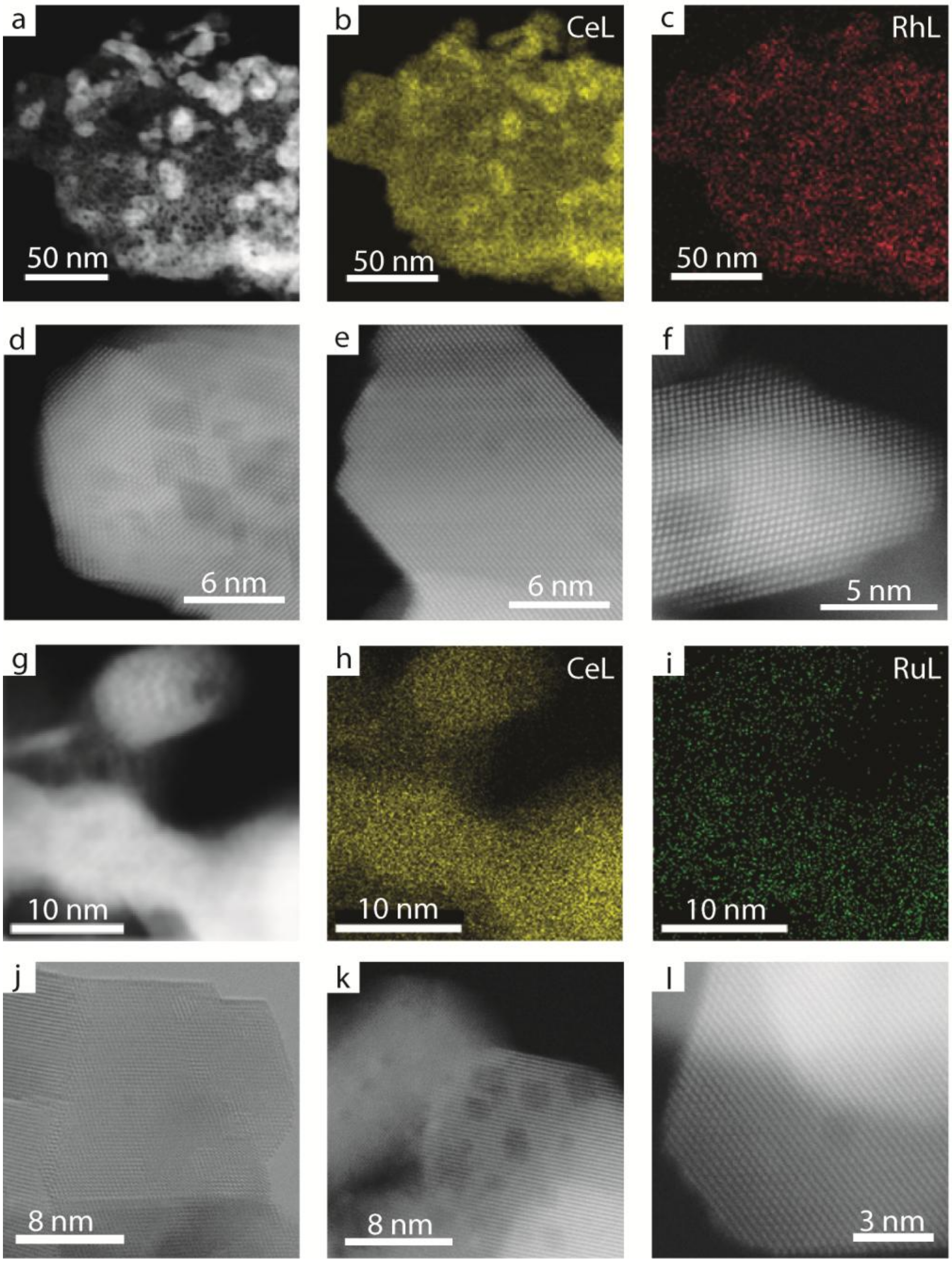

Figure S30: C_s-HAADF-STEM and corresponding EDX compositional nanoscale maps for (a-f) 1.0Rh/CeO_2 and (g-l) 1.0Ru/CeO_2 catalysts recovered after the tandem isomerization-hydrosilylation of 1-octene.

4. Supplementary Tables

Table S1: Summary of optimized parameters by fitting EXAFS data recorded for as-synthesized Pt/CeO_2 catalysts with different metal contents at room temperature.

Catalyst	CN[a]	Path	R(Å)	σ^2 ($Å^2$)[b]	ES[c] (eV)	r-factor
0.2Pt/CeO_2	5.6 ± 0.7	Pt-O	1.996 ± 0.005	0.0018 ± 0.0006	10.5±0.6	0.0035
0.5Pt/CeO_2	5.2 ± 0.6	Pt-O				
1.0Pt/CeO_2	4.8 ± 0.4	Pt-O				
2.0Pt/CeO_2	4.7 ± 0.4	Pt-O				
5.0Pt/CeO_2	3.0 ± 0.3	Pt-O				

EXAFS spectra fits were performed on the first coordination shell over the FT of the k^3-weighted χ(k) function in the Δk= 3-12 $Å^{-1}$ interval. The amplitude reduction factor S_0^2 was fixed to 0.82 relative to the pure metal. [a] Average coordination number. [b] Mean square variation in path length. [c] Energy shift. The r-factor represents the goodness of fit.

Table S2: Summary of optimized parameters by fitting EXAFS data recorded for as-synthesized Rh/CeO_2 catalysts with different metal contents at room temperature.

Catalyst	CN[a]	Path	R(Å)	σ^2 ($Å^2$)[b]	ES[c] (eV)	r-factor
0.2Rh/CeO_2	5.5 ± 0.7	Rh-O	2.031 ± 0.002	0.0028 ± 0.0004	-3.3±0.5	0.0145
0.5Rh/CeO_2	4.9 ± 0.4	Rh-O				
1.0Rh/CeO_2	4.4 ± 0.4	Rh-O				
2.0Rh/CeO_2	5.4 ± 0.4	Rh-O				
5.0Rh/CeO_2	5.1 ± 0.2	Rh-O				
10Rh/CeO_2	5.8 ± 0.3	Rh-O				

EXAFS spectra fits were performed on the first coordination shell over the FT of the k^3-weighted χ(k) function in the Δk= 3-12 $Å^{-1}$ interval. The amplitude reduction factor S_0^2 was fixed to 0.82 relative to the pure metal. [a]Average coordination number. [b] Mean square variation in path length. [c] Energy shift. The r-factor represents the goodness of fit.

Table S3: Summary of optimized parameters by fitting EXAFS data recorded for as-synthesized Ru/CeO_2 catalysts with different metal contents at room temperature.

Catalyst	CN[a]	Path	R(Å)	σ^2 ($Å^2$)[b]	ES[c] (eV)	r-factor
$0.2Ru/CeO_2$	5.6 ± 0.4	Ru-O	1.981 ± 0.005	0.0071 ± 0.0001	10.3±0.6	0.0085
$0.5Ru/CeO_2$	5.3 ± 0.4	Ru-O				
$1.0Ru/CeO_2$	4.3 ± 0.3	Ru-O				
$2.0Ru/CeO_2$	5.2 ± 0.4	Ru-O				

EXAFS spectra fits were performed on the first coordination shell over the FT of the k^3-weighted χ(k) function in the Δk= 3-12 $Å^{-1}$ interval. The amplitude reduction factor S_0^2 was fixed to 0.78 relative to the pure metal. [a]Average coordination number. [b]Mean square variation in path length. [c]Energy shift. The r-factor represents the goodness of fit.

The evolution of the first-shell *M-O* average coordination number (CN), derived from the fitting of the EXAFS spectra, was assessed as a function of the surface metal content (**Tables S1-S3**). The results reveal first a decrease in CN as δ increases up to 1.0 M_{at} nm^{-2}, followed by an augment in CN for increasingly higher metal contents beyond this value. This result suggests that, at any metal content, a fraction of the metal atoms incorporates into sub-surface positions within the CeO_2 structure, being coordinatively saturated and thus contributing to a higher average CN at very low metal contents, i.e. 0.2 M_{at} nm^{-2}, at which their abundance is highest. On increasing the overall metal content, the solid-state solubility of metal cations in the CeO_2 lattice is likely exceeded, and metal species are consequently "spelled" onto the CeO_2 surface, according to our EXAFS results, as isolated (coordinatively unsaturated) metal cations up to a surface coverage of 1.0-2.0 M_{at} nm^{-2}. Further rise in metal content leads to partial aggregation of surface metal species into oxide nanoparticles, as proved by EXAFS and XRD, and thus the average 1st-shell CN is set to increase again. This trend is observed regardless of the nature of the supported metal, and it thus reflects a generalized behavior.

Table S4: Catalytic results for the hydrosilylation of various α-olefin substrates with triethylsilane using a single-atom Rh_1/CeO_2 as catalyst ($1.0Rh/CeO_2$).

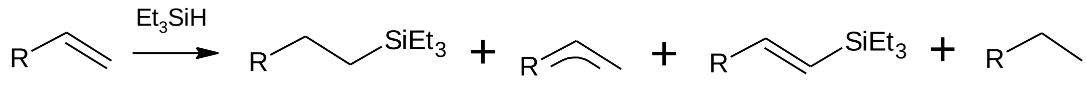

1 *2* *3* *4*

Entry	Catalyst	Olefin R-fragment	Silane	T (K)	Time (h)	X (%)	Product selectivity (%)			
							1	*2*	*3*	*4*
1	$1.0Rh/CeO_2$	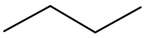	Et_3SiH	393	5	99	98	2	-	-
2	$1.0Rh/CeO_2$	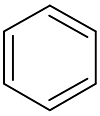	Et_3SiH	393	5	87	87	-	7	6
3	$1.0Rh/CeO_2$	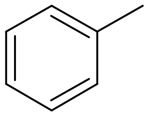	Et_3SiH	393	5	99	98	2	-	-
4	$1.0Rh/CeO_2$	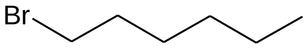	Et_3SiH	393	5	98	95	5	-	-
5	$1.0Rh/CeO_2$	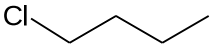	Et_3SiH	393	5	99	98	2	-	-
6	$1.0Rh/CeO_2$	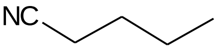	Et_3SiH	393	5	80	97	3	-	-
7	$1.0Rh/CeO_2$	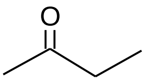	Et_3SiH	393	5	98	97	3	-	-
8	$1.0Rh/CeO_2$	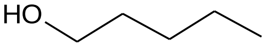	Et_3SiH	393	18	99	96	-	-	-
9	$1.0Rh/CeO_2$	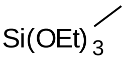	Et_3SiH	383	5	98	78	5	-	13

Reaction Conditions: 1-octene (5 mmol), triethylsilane (5 mmol), catalyst (2 µmol, metal basis), P=10 bar (N_2, 99.999% purity).

Excellent yields and selectivities to terminal alkyl silanes were obtained with linear 1-olefin reactants of different hydrocarbon chain lengths. Styrene, which is a particularly challenging substrate given that the bulkiness of the phenyl substituent is known to favor β-hydride elimination and thus dehydrogenative hydrosilylation pathways,[19] was converted under standard reaction conditions with remarkably good yields to the anti-Markovnikov terminal hydrosilylation product. A remarkable 87% selectivity was achieved at 80% conversion with $1.0Rh/CeO_2$. In sharp contrast a 25% olefin conversion with a marginal 22% selectivity was obtained applying the $1.0Pt/CeO_2$ analog under identical reaction settings, owing to the massive production of unsaturated silanes (dehydrogenative hydrosilylation) and ethylbenzene (hydrogenation) side-products. Moreover, $1.0Rh/CeO_2$ showed a remarkable tolerance to various functional groups

Table S5: Catalytic results for the hydrosilylation of allyl alcohol (2-Propen-1-ol) with triethylsilane using different M/CeO_2 single-atom catalysts.

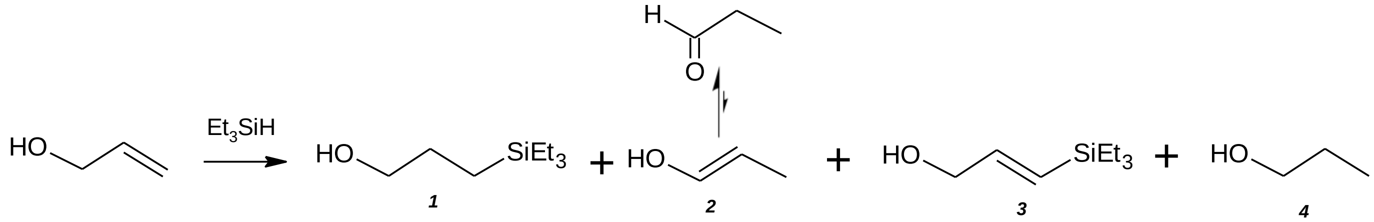

Entry	Catalyst	Olefin substrate	Silane	T (K)	Time (h)	X (%)	Product selectivity (%)[a]			
							1	***2***[b]	***3***	***4***
1	1.0Rh/CeO_2	2-Propen-1-ol	-	393	18	7	-	>99	-	-
2	1.0Rh/CeO_2	2-Propen-1-ol	Et_3SiH	393	18	99	92	3	-	-
3	1.0Pt/CeO_2	2-Propen-1-ol	Et_3SiH	393	18	82	55	40	-	1
4	1.0Ru/CeO_2	2-Propen-1-ol	-	393	18	<1	-	-	-	-
5	1.0Ru/CeO_2	2-Propen-1-ol	Et_3SiH	393	18	17	-	>99	-	1

[a]O-silylated product was also observed (3-4%). [b]Propanal was detected as the thermodynamically most stable tautomer of product ***2***.

Table S6: DFT-computed electronic energies in eV. Gibbs free energies (G:PBE-D3/400eV) were constructed from translational, rotational, vibrational and zero point energy (zpE) contributions at a temperature of 393 K and a reference pressure of 10 bar.

Species	Trans. + Rot.	Vib.	zpE	G:PBE-D3/400eV	PBE-D3/400eV
H_2	-0.3679936	-0.0154	0.28	-6.8620186	-6.759625
CO	-0.6420499	-0.0034	0.13	-15.309059	-14.798595
Propene	-0.8330709	-0.0488	2.1	-47.483842	-48.709872
TMS-H	-0.8873232	-0.1802	3.14	-67.83942	-69.915837
TMS-propane	-1.0026429	-0.4081	5.4	-115.96171	-119.95287
TMS-TMS	-1.0304104	-0.5836	5.86	-128.58628	-132.83319
1x3-CeO_2-211_slab	0	0	0	-795.18485	-795.18485
RhH_1x3-CeO_2-211	0	-0.0984	0.23	-805.18609	-805.3246
RhH_2allyl_1x3-CeO_2-211	0	-0.2759	2.32	-852.79323	-854.84224
RhH_2SiMe_3_1x3-CeO_2-211	0	-0.5479	3.39	-873.66239	-876.51244
RhH_2SiMe_3propene_1x3-CeO_2-211	0	-0.7503	5.57	-920.49874	-925.32536
RhHpropene_1x3-CeO_2-211	0	-0.3283	2.39	-853.34436	-855.406
$RhHSiMe_3$isopropyl_1x3-CeO_2-211	0	-0.7626	5.62	-920.81566	-925.68206
$RhHSiMe_3$propyl_1x3-CeO_2-211	0	-0.7134	5.65	-921.43584	-926.37442
TS_RhH_2SiMe3propene_$RhHSiMe_3$propyl_1x3-CeO2-211	0	-0.6859	5.56	-920.27765	-925.15773
TS_$RhHSiMe_3$propyl_RhH_1x3-CeO_2-211	0	-0.7346	5.63	-920.79763	-925.69294
TS_RhH_2SiMe_3propene_$RhHSiMe_3$isopropyl_1x3-CeO2-211	0	-0.7605	5.55	-920.39379	-925.18819
TS_RhHpropen_RhH2allyl_1x3-CeO_2-211	0	-0.289	2.27	-852.26466	-854.24559
Ru_1x3-CeO_2-211	0	-0.0937	0.03	-802.6475	-802.58573
Rudipropene_1x3-CeO_2-211	0	-0.4881	4.37	-898.37435	-902.25723
RuHallyl_1x3-CeO_2-211	0	-0.2528	2.1	-851.13618	-852.98638
$RuHSiMe_3$_1x3-CeO_2-211	0	-0.5649	3.16	-871.60839	-874.2084
$RuHSiMe_3$propene_1x3-CeO_2-211	0	-0.7293	5.37	-918.83254	-923.47823
Rupropene_1x3-CeO_2-211	0	-0.3162	2.18	-850.93198	-852.79971
$RuSiMe_3$isopropyl_1x3-CeO_2-211	0	-0.6565	5.47	-918.89482	-923.7083
TS_$RuHSiMe_3$propene_$RuSiMe_3$isopropyl_1x3-CeO_2-211	0	-0.6817	5.34	-918.69354	-923.35484
TS_Rupropene_RuHallyl_1x3-CeO_2-211	0	-0.2824	2.07	-850.28895	-852.08352

5. References

[1] J. Jones, H. Xiong, A. T. DeLaRiva, E. J. Peterson, H. Pham, S. R. Challa, G. Qi, S. Oh, M. H. Wiebenga, X. I. Pereira Hernández, Y. Wang, A. K. Datye, *Science* **2016**, *353*, 150-154.

[2] L. Simonelli, C. Marini, W. Olszewski, M. Ávila Pérez, N. Ramanan, G. Guilera, V. Cuartero, K. Klementiev, *Cogent Physics* **2016**, *3*, 1231987.

[3] G. Guilera, F. Rey, J. Hernández-Fenollosa, J. J. Cortés-Vergaz, *Journal of Physics: Conference Series* **2013**, *430*, 012057.

[4] B. Ravel, M. Newville, *Journal of Synchrotron Radiation* **2005**, *12*, 537-541.

[5] aG. Kresse, D. Joubert, *Physical Review B* **1999**, *59*, 1758-1775; bG. Kresse, J. Furthmüller, *Physical Review B* **1996**, *54*, 11169-11186; cP. E. Blöchl, *Physical Review B* **1994**, *50*, 17953-17979.

[6] J. P. Perdew, K. Burke, M. Ernzerhof, *Physical Review Letters* **1996**, *77*, 3865-3868.

[7] H. J. Monkhorst, J. D. Pack, *Physical Review B* **1976**, *13*, 5188-5192.

[8] S. M. Kozlov, F. Viñes, N. Nilius, S. Shaikhutdinov, K. M. Neyman, *The Journal of Physical Chemistry Letters* **2012**, *3*, 1956-1961.

[9] F. Dvořák, M. Farnesi Camellone, A. Tovt, N.-D. Tran, F. R. Negreiros, M. Vorokhta, T. Skála, I. Matolínová, J. Mysliveček, V. Matolín, S. Fabris, *Nature Communications* **2016**, *7*, 10801.

[10] P. N. Plessow, *Journal of Chemical Theory and Computation* **2018**, *14*, 981-990.

[11] E. Mamontov, T. Egami, R. Brezny, M. Koranne, S. Tyagi, *The Journal of Physical Chemistry B* **2000**, *104*, 11110-11116.

[12] M. S. Brogan, T. J. Dines, J. A. Cairns, *Journal of the Chemical Society, Faraday Transactions* **1994**, *90*, 1461-1466.

[13] L. S. Kibis, T. Y. Kardash, E. A. Derevyannikova, O. A. Stonkus, E. M. Slavinskaya, V. A. Svetlichnyi, A. I. Boronin, *The Journal of Physical Chemistry C* **2017**, *121*, 26925-26938.

[14] Z. Ma, S. Zhao, X. Pei, X. Xiong, B. Hu, *Catalysis Science & Technology* **2017**, *7*, 191-199.

[15] M. Manzoli, F. Boccuzzi, A. Chiorino, F. Vindigni, W. Deng, M. Flytzani-Stephanopoulos, *Journal of Catalysis* **2007**, *245*, 308-315.

[16] P. Serna, D. Yardimci, J. D. Kistler, B. C. Gates, *Physical Chemistry Chemical Physics* **2014**, *16*, 1262-1270.

[17] C. J. Yue, Y. Liu, R. He, *Journal of Molecular Catalysis A: Chemical* **2006**, *259*, 17-23.

[18] S. Gutiérrez-Tarriño, P. Concepción, P. Oña-Burgos, *European Journal of Inorganic Chemistry* **2018**, *2018*, 4867-4874.

[19] A. Onopchenko, E. T. Sabourin, D. L. Beach, *The Journal of Organic Chemistry* **1983**, *48*, 5101-5105.

6. Optimized DFT structures

H2

```
data_image0
_cell_length_a       16
_cell_length_b       16
_cell_length_c       17
_cell_angle_alpha    90
_cell_angle_beta     90
_cell_angle_gamma    90

_symmetry_space_group_name_H-M    "P 1"
_symmetry_int_tables_number       1

loop_
  _symmetry_equiv_pos_as_xyz
  'x, y, z'

loop_
  _atom_site_label
  _atom_site_occupancy
  _atom_site_fract_x
  _atom_site_fract_y
  _atom_site_fract_z
  _atom_site_thermal_displace_type
  _atom_site_B_iso_or_equiv
  _atom_site_type_symbol
  H1       1.0000 0.50000  0.50000  0.47791  Biso   1.000  H
  H2       1.0000 0.50000  0.50000  0.52209  Biso   1.000  H
```

CO

```
data_image0
_cell_length_a       16.7531
_cell_length_b       16.2928
_cell_length_c       16.6723
_cell_angle_alpha    90
_cell_angle_beta     90
_cell_angle_gamma    90

_symmetry_space_group_name_H-M    "P 1"
_symmetry_int_tables_number       1

loop_
  _symmetry_equiv_pos_as_xyz
  'x, y, z'

loop_
  _atom_site_label
  _atom_site_occupancy
  _atom_site_fract_x
  _atom_site_fract_y
  _atom_site_fract_z
  _atom_site_thermal_displace_type
  _atom_site_B_iso_or_equiv
  _atom_site_type_symbol
  C1       1.0000 0.47555  0.50978  0.47806  Biso   1.000  C
  O1       1.0000 0.52445  0.49022  0.52194  Biso   1.000  O
```

```
propen
data_image0
_cell_length_a       17.4224
_cell_length_b       18.7655
_cell_length_c       19.5856
_cell_angle_alpha    90
_cell_angle_beta     90
_cell_angle_gamma    90

_symmetry_space_group_name_H-M    "P 1"
_symmetry_int_tables_number       1

loop_
  _symmetry_equiv_pos_as_xyz
  'x, y, z'

loop_
  _atom_site_label
  _atom_site_occupancy
  _atom_site_fract_x
  _atom_site_fract_y
  _atom_site_fract_z
  _atom_site_thermal_displace_type
  _atom_site_B_iso_or_equiv
  _atom_site_type_symbol
  C1       1.0000 0.49618  0.44536  0.45531  Biso   1.000  C
  C2       1.0000 0.50525  0.51861  0.48444  Biso   1.000  C
  H1       1.0000 0.54898  0.42806  0.42903  Biso   1.000  H
  H2       1.0000 0.48264  0.40605  0.49517  Biso   1.000  H
  C3       1.0000 0.49946  0.53531  0.55056  Biso   1.000  C
  H3       1.0000 0.48713  0.49472  0.58911  Biso   1.000  H
  H4       1.0000 0.50675  0.58991  0.56862  Biso   1.000  H
  H5       1.0000 0.45004  0.44392  0.41678  Biso   1.000  H
  H6       1.0000 0.51770  0.56116  0.44769  Biso   1.000  H


TMS-H
data_image0
_cell_length_a       19.7349
_cell_length_b       20.3024
_cell_length_c       20.0787
_cell_angle_alpha    90
_cell_angle_beta     90
_cell_angle_gamma    90

_symmetry_space_group_name_H-M    "P 1"
_symmetry_int_tables_number       1

loop_
  _symmetry_equiv_pos_as_xyz
  'x, y, z'

loop_
  _atom_site_label
  _atom_site_occupancy
  _atom_site_fract_x
  _atom_site_fract_y
  _atom_site_fract_z
  _atom_site_thermal_displace_type
  _atom_site_B_iso_or_equiv
```

```
  _atom_site_type_symbol
  Si1      1.0000 0.47273  0.50888  0.48242  Biso   1.000  Si
  C1       1.0000 0.55408  0.55381  0.46396  Biso   1.000  C
  H1       1.0000 0.59713  0.53040  0.48963  Biso   1.000  H
  H2       1.0000 0.55153  0.60542  0.48052  Biso   1.000  H
  H3       1.0000 0.56518  0.55379  0.41025  Biso   1.000  H
  C2       1.0000 0.45395  0.51127  0.57437  Biso   1.000  C
  H4       1.0000 0.44916  0.56226  0.59237  Biso   1.000  H
  H5       1.0000 0.49475  0.48740  0.60298  Biso   1.000  H
  H6       1.0000 0.40633  0.48545  0.58587  Biso   1.000  H
  C3       1.0000 0.47827  0.42106  0.45268  Biso   1.000  C
  H7       1.0000 0.51962  0.39476  0.47818  Biso   1.000  H
  H8       1.0000 0.48807  0.41868  0.39877  Biso   1.000  H
  H9       1.0000 0.43088  0.39423  0.46262  Biso   1.000  H
  H10      1.0000 0.41598  0.54251  0.44601  Biso   1.000  H


TMS-propan
data_image0
_cell_length_a       22.0661
_cell_length_b       21.3287
_cell_length_c       22.5208
_cell_angle_alpha    90
_cell_angle_beta     90
_cell_angle_gamma    90

_symmetry_space_group_name_H-M    "P 1"
_symmetry_int_tables_number       1

loop_
  _symmetry_equiv_pos_as_xyz
  'x, y, z'

loop_
  _atom_site_label
  _atom_site_occupancy
  _atom_site_fract_x
  _atom_site_fract_y
  _atom_site_fract_z
  _atom_site_thermal_displace_type
  _atom_site_B_iso_or_equiv
  _atom_site_type_symbol
  C1       1.0000 0.41077  0.56603  0.39780  Biso   1.000  C
  C2       1.0000 0.46332  0.53155  0.42777  Biso   1.000  C
  H1       1.0000 0.36696  0.54507  0.41028  Biso   1.000  H
  H2       1.0000 0.40960  0.61564  0.41135  Biso   1.000  H
  C3       1.0000 0.45954  0.53210  0.49586  Biso   1.000  C
  H3       1.0000 0.41670  0.50984  0.51019  Biso   1.000  H
  H4       1.0000 0.45694  0.58106  0.51186  Biso   1.000  H
  H5       1.0000 0.41436  0.56454  0.34906  Biso   1.000  H
  H6       1.0000 0.46451  0.48263  0.41181  Biso   1.000  H
  H7       1.0000 0.50654  0.55276  0.41337  Biso   1.000  H
  Si1      1.0000 0.52432  0.49214  0.53608  Biso   1.000  Si
  C4       1.0000 0.59754  0.53463  0.52004  Biso   1.000  C
  H8       1.0000 0.63589  0.51216  0.54297  Biso   1.000  H
  H9       1.0000 0.59540  0.58372  0.53504  Biso   1.000  H
  H10      1.0000 0.60793  0.53492  0.47223  Biso   1.000  H
  C5       1.0000 0.50896  0.49398  0.61844  Biso   1.000  C
  H11      1.0000 0.50456  0.54246  0.63469  Biso   1.000  H
  H12      1.0000 0.54584  0.47150  0.64357  Biso   1.000  H
```

H13 1.0000 0.46663 0.46914 0.62930 Biso 1.000 H
C6 1.0000 0.53073 0.40815 0.51050 Biso 1.000 C
H14 1.0000 0.56802 0.38378 0.53336 Biso 1.000 H
H15 1.0000 0.53955 0.40531 0.46246 Biso 1.000 H
H16 1.0000 0.48871 0.38200 0.51958 Biso 1.000 H

1x3-CeO2-211_slab
data_image0
loop_
_atom_site_label
_atom_site_occupancy
_atom_site_Cartn_x
_atom_site_Cartn_y
_atom_site_Cartn_z
_atom_site_thermal_displace_type
_atom_site_B_iso_or_equiv
_atom_site_type_symbol
Ce1 1.0000 -0.77087 1.93685 10.23856 Biso 1.000 Ce
Ce2 1.0000 -0.73045 1.93732 16.90647 Biso 1.000 Ce
O1 1.0000 0.02059 1.93685 8.00000 Biso 1.000 O
O2 1.0000 1.60349 1.93685 10.23856 Biso 1.000 O
O3 1.0000 0.00997 1.93705 14.71206 Biso 1.000 O
Ce3 1.0000 5.56074 3.87550 9.11928 Biso 1.000 Ce
Ce4 1.0000 2.39494 1.93685 8.00000 Biso 1.000 Ce
O4 1.0000 4.76929 1.93685 8.00000 Biso 1.000 O
O5 1.0000 7.93510 3.87550 9.11928 Biso 1.000 O
O6 1.0000 3.18639 3.87550 9.11928 Biso 1.000 O
O7 1.0000 6.35219 1.93685 10.23856 Biso 1.000 O
Ce5 1.0000 2.39494 3.87550 11.35784 Biso 1.000 Ce
Ce6 1.0000 -0.78892 3.87560 13.56336 Biso 1.000 Ce
Ce7 1.0000 5.56074 1.93685 12.47712 Biso 1.000 Ce
O8 1.0000 7.93510 1.93685 12.47712 Biso 1.000 O
O9 1.0000 3.18639 1.93685 12.47712 Biso 1.000 O
O10 1.0000 4.76929 3.87550 11.35784 Biso 1.000 O
O11 1.0000 0.02059 3.87550 11.35784 Biso 1.000 O
Ce8 1.0000 2.39150 1.93720 14.69069 Biso 1.000 Ce
O12 1.0000 1.58918 3.87571 13.55008 Biso 1.000 O
O13 1.0000 6.32020 3.87571 13.57662 Biso 1.000 O
O14 1.0000 4.83158 1.93728 14.81467 Biso 1.000 O
Ce9 1.0000 5.56725 3.87597 15.89087 Biso 1.000 Ce
Ce10 1.0000 2.31939 3.87602 18.01275 Biso 1.000 Ce
O15 1.0000 1.58918 1.93736 16.88753 Biso 1.000 O
O16 1.0000 6.48038 1.93743 17.24759 Biso 1.000 O
O17 1.0000 7.89375 3.87586 15.79456 Biso 1.000 O
O18 1.0000 3.12398 3.87596 15.76136 Biso 1.000 O
Ce11 1.0000 5.27318 1.93751 19.29582 Biso 1.000 Ce
O19 1.0000 4.58671 3.87621 17.92332 Biso 1.000 O
O20 1.0000 0.00149 3.87592 18.02046 Biso 1.000 O
O21 1.0000 3.01792 1.93751 19.19221 Biso 1.000 O
O22 1.0000 5.80330 3.87621 20.22717 Biso 1.000 O
Ce12 1.0000 -0.77087 5.81416 10.23856 Biso 1.000 Ce
Ce13 1.0000 -0.73046 5.81462 16.90647 Biso 1.000 Ce
O23 1.0000 0.02059 5.81416 8.00000 Biso 1.000 O
O24 1.0000 1.60349 5.81416 10.23856 Biso 1.000 O
O25 1.0000 0.00997 5.81435 14.71206 Biso 1.000 O
Ce14 1.0000 5.56074 7.75281 9.11928 Biso 1.000 Ce
Ce15 1.0000 2.39494 5.81416 8.00000 Biso 1.000 Ce
O26 1.0000 4.76929 5.81416 8.00000 Biso 1.000 O
O27 1.0000 7.93510 7.75281 9.11928 Biso 1.000 O

O28	1.0000	3.18639	7.75281	9.11928	Biso	1.000	O
O29	1.0000	6.35219	5.81416	10.23856	Biso	1.000	O
Ce16	1.0000	2.39494	7.75281	11.35784	Biso	1.000	Ce
Ce17	1.0000	-0.78892	7.75291	13.56336	Biso	1.000	Ce
Ce18	1.0000	5.56074	5.81416	12.47712	Biso	1.000	Ce
O30	1.0000	7.93510	5.81416	12.47712	Biso	1.000	O
O31	1.0000	3.18639	5.81416	12.47712	Biso	1.000	O
O32	1.0000	4.76929	7.75281	11.35784	Biso	1.000	O
O33	1.0000	0.02059	7.75281	11.35784	Biso	1.000	O
Ce19	1.0000	2.39150	5.81450	14.69069	Biso	1.000	Ce
O34	1.0000	1.58918	7.75301	13.55008	Biso	1.000	O
O35	1.0000	6.32020	7.75301	13.57662	Biso	1.000	O
O36	1.0000	4.83158	5.81458	14.81467	Biso	1.000	O
Ce20	1.0000	5.56725	7.75327	15.89087	Biso	1.000	Ce
Ce21	1.0000	2.31939	7.75332	18.01275	Biso	1.000	Ce
O37	1.0000	1.58919	5.81467	16.88753	Biso	1.000	O
O38	1.0000	6.48038	5.81473	17.24759	Biso	1.000	O
O39	1.0000	7.89375	7.75316	15.79456	Biso	1.000	O
O40	1.0000	3.12398	7.75327	15.76136	Biso	1.000	O
Ce22	1.0000	5.27319	5.81481	19.29582	Biso	1.000	Ce
O41	1.0000	4.58671	7.75351	17.92332	Biso	1.000	O
O42	1.0000	0.00149	7.75322	18.02046	Biso	1.000	O
O43	1.0000	3.01792	5.81481	19.19221	Biso	1.000	O
O44	1.0000	5.80330	7.75351	20.22717	Biso	1.000	O
Ce23	1.0000	-0.77087	9.69146	10.23856	Biso	1.000	Ce
Ce24	1.0000	-0.73046	9.69192	16.90647	Biso	1.000	Ce
O45	1.0000	0.02059	9.69146	8.00000	Biso	1.000	O
O46	1.0000	1.60349	9.69146	10.23856	Biso	1.000	O
O47	1.0000	0.00997	9.69165	14.71206	Biso	1.000	O
Ce25	1.0000	5.56074	11.63011	9.11928	Biso	1.000	Ce
Ce26	1.0000	2.39494	9.69146	8.00000	Biso	1.000	Ce
O48	1.0000	4.76929	9.69146	8.00000	Biso	1.000	O
O49	1.0000	7.93510	11.63011	9.11928	Biso	1.000	O
O50	1.0000	3.18639	11.63011	9.11928	Biso	1.000	O
O51	1.0000	6.35219	9.69146	10.23856	Biso	1.000	O
Ce27	1.0000	2.39494	11.63011	11.35784	Biso	1.000	Ce
Ce28	1.0000	-0.78892	11.63021	13.56336	Biso	1.000	Ce
Ce29	1.0000	5.56074	9.69146	12.47712	Biso	1.000	Ce
O52	1.0000	7.93510	9.69146	12.47712	Biso	1.000	O
O53	1.0000	3.18639	9.69146	12.47712	Biso	1.000	O
O54	1.0000	4.76929	11.63011	11.35784	Biso	1.000	O
O55	1.0000	0.02059	11.63011	11.35784	Biso	1.000	O
Ce30	1.0000	2.39150	9.69180	14.69069	Biso	1.000	Ce
O56	1.0000	1.58918	11.63031	13.55008	Biso	1.000	O
O57	1.0000	6.32020	11.63032	13.57662	Biso	1.000	O
O58	1.0000	4.83158	9.69188	14.81467	Biso	1.000	O
Ce31	1.0000	5.56725	11.63058	15.89087	Biso	1.000	Ce
Ce32	1.0000	2.31939	11.63062	18.01275	Biso	1.000	Ce
O59	1.0000	1.58919	9.69197	16.88753	Biso	1.000	O
O60	1.0000	6.48038	9.69203	17.24759	Biso	1.000	O
O61	1.0000	7.89375	11.63046	15.79456	Biso	1.000	O
O62	1.0000	3.12398	11.63057	15.76136	Biso	1.000	O
Ce33	1.0000	5.27319	9.69211	19.29582	Biso	1.000	Ce
O63	1.0000	4.58671	11.63081	17.92332	Biso	1.000	O
O64	1.0000	0.00149	11.63052	18.02046	Biso	1.000	O
O65	1.0000	3.01791	9.69211	19.19221	Biso	1.000	O
O66	1.0000	5.80330	11.63081	20.22717	Biso	1.000	O

RhH_1x3-CeO2-211

```
data_image0
loop_
  _atom_site_label
  _atom_site_occupancy
  _atom_site_Cartn_x
  _atom_site_Cartn_y
  _atom_site_Cartn_z
  _atom_site_thermal_displace_type
  _atom_site_B_iso_or_equiv
  _atom_site_type_symbol
  Ce1      1.0000 -0.77087  1.93685  10.23856  Biso   1.000  Ce
  Ce2      1.0000 -0.75758  1.91330  16.91457  Biso   1.000  Ce
  O1       1.0000 0.02059  1.93685  8.00000  Biso   1.000  O
  O2       1.0000 1.60349  1.93685  10.23856  Biso   1.000  O
  O3       1.0000 0.01082  1.92605  14.71966  Biso   1.000  O
  Ce3      1.0000 5.56074  3.87550  9.11928  Biso   1.000  Ce
  Ce4      1.0000 2.39494  1.93685  8.00000  Biso   1.000  Ce
  O4       1.0000 4.76929  1.93685  8.00000  Biso   1.000  O
  O5       1.0000 7.93510  3.87550  9.11928  Biso   1.000  O
  O6       1.0000 3.18639  3.87550  9.11928  Biso   1.000  O
  O7       1.0000 6.35219  1.93685  10.23856  Biso   1.000  O
  Ce5      1.0000 2.39494  3.87550  11.35784  Biso   1.000  Ce
  Ce6      1.0000 -0.78819  3.87174  13.56183  Biso   1.000  Ce
  Ce7      1.0000 5.56074  1.93685  12.47712  Biso   1.000  Ce
  O8       1.0000 7.93510  1.93685  12.47712  Biso   1.000  O
  O9       1.0000 3.18639  1.93685  12.47712  Biso   1.000  O
  O10      1.0000 4.76929  3.87550  11.35784  Biso   1.000  O
  O11      1.0000 0.02059  3.87550  11.35784  Biso   1.000  O
  Ce8      1.0000 2.38372  1.94348  14.69367  Biso   1.000  Ce
  O12      1.0000 1.59768  3.87642  13.57306  Biso   1.000  O
  O13      1.0000 6.32020  3.87571  13.57662  Biso   1.000  O
  O14      1.0000 4.81370  1.94253  14.78829  Biso   1.000  O
  Ce9      1.0000 5.57853  3.83058  15.90873  Biso   1.000  Ce
  Ce10     1.0000 2.36892  3.86911  18.04501  Biso   1.000  Ce
  O15      1.0000 1.57292  1.96665  16.92114  Biso   1.000  O
  O16      1.0000 6.38352  1.86719  17.11237  Biso   1.000  O
  O17      1.0000 7.90841  3.87099  15.78289  Biso   1.000  O
  O18      1.0000 3.15416  3.86746  15.78512  Biso   1.000  O
  Ce11     1.0000 5.33430  1.86245  19.23785  Biso   1.000  Ce
  O19      1.0000 4.59459  3.87330  17.98488  Biso   1.000  O
  O20      1.0000 -0.01166  3.91523  18.11930  Biso   1.000  O
  O21      1.0000 3.06706  1.91923  19.20295  Biso   1.000  O
  O22      1.0000 6.07015  3.89284  20.20490  Biso   1.000  O
  Ce12     1.0000 -0.77087  5.81416  10.23856  Biso   1.000  Ce
  Ce13     1.0000 -0.68658  5.85440  16.89815  Biso   1.000  Ce
  O23      1.0000 0.02059  5.81416  8.00000  Biso   1.000  O
  O24      1.0000 1.60349  5.81416  10.23856  Biso   1.000  O
  O25      1.0000 0.04015  5.82759  14.69974  Biso   1.000  O
  Ce14     1.0000 5.56074  7.75281  9.11928  Biso   1.000  Ce
  Ce15     1.0000 2.39494  5.81416  8.00000  Biso   1.000  Ce
  O26      1.0000 4.76929  5.81416  8.00000  Biso   1.000  O
  O27      1.0000 7.93510  7.75281  9.11928  Biso   1.000  O
  O28      1.0000 3.18639  7.75281  9.11928  Biso   1.000  O
  O29      1.0000 6.35219  5.81416  10.23856  Biso   1.000  O
  Ce16     1.0000 2.39494  7.75281  11.35784  Biso   1.000  Ce
  Ce17     1.0000 -0.78201  7.74558  13.54813  Biso   1.000  Ce
  Ce18     1.0000 5.56074  5.81416  12.47712  Biso   1.000  Ce
  O30      1.0000 7.93510  5.81416  12.47712  Biso   1.000  O
  O31      1.0000 3.18639  5.81416  12.47712  Biso   1.000  O
  O32      1.0000 4.76929  7.75281  11.35784  Biso   1.000  O
```

O33 1.0000 0.02059 7.75281 11.35784 Biso 1.000 O
Ce19 1.0000 2.40559 5.80800 14.69686 Biso 1.000 Ce
O34 1.0000 1.59842 7.75491 13.56034 Biso 1.000 O
O35 1.0000 6.32020 7.75301 13.57662 Biso 1.000 O
O36 1.0000 4.88138 5.81874 14.89390 Biso 1.000 O
Ce20 1.0000 5.54979 7.81175 15.85030 Biso 1.000 Ce
Ce21 1.0000 2.33631 7.75788 18.00884 Biso 1.000 Ce
O37 1.0000 1.63293 5.79888 16.90231 Biso 1.000 O
O38 1.0000 6.68079 5.60813 17.60153 Biso 1.000 O
O39 1.0000 7.85208 7.71742 15.81365 Biso 1.000 O
O40 1.0000 3.13434 7.76711 15.76798 Biso 1.000 O
Ce22 1.0000 5.26471 5.90319 19.36220 Biso 1.000 Ce
O41 1.0000 4.64504 7.68721 17.91789 Biso 1.000 O
O42 1.0000 0.04057 7.78612 18.00718 Biso 1.000 O
O43 1.0000 3.01761 5.83380 19.23512 Biso 1.000 O
O44 1.0000 5.77683 7.83104 20.29450 Biso 1.000 O
Ce23 1.0000 -0.77087 9.69146 10.23856 Biso 1.000 Ce
Ce24 1.0000 -0.72631 9.69626 16.90114 Biso 1.000 Ce
O45 1.0000 0.02059 9.69146 8.00000 Biso 1.000 O
O46 1.0000 1.60349 9.69146 10.23856 Biso 1.000 O
O47 1.0000 -0.00033 9.66995 14.71683 Biso 1.000 O
Ce25 1.0000 5.56074 11.63011 9.11928 Biso 1.000 Ce
Ce26 1.0000 2.39494 9.69146 8.00000 Biso 1.000 Ce
O48 1.0000 4.76929 9.69146 8.00000 Biso 1.000 O
O49 1.0000 7.93510 11.63011 9.11928 Biso 1.000 O
O50 1.0000 3.18639 11.63011 9.11928 Biso 1.000 O
O51 1.0000 6.35219 9.69146 10.23856 Biso 1.000 O
Ce27 1.0000 2.39494 11.63011 11.35784 Biso 1.000 Ce
Ce28 1.0000 -0.78894 11.63161 13.55206 Biso 1.000 Ce
Ce29 1.0000 5.56074 9.69146 12.47712 Biso 1.000 Ce
O52 1.0000 7.93510 9.69146 12.47712 Biso 1.000 O
O53 1.0000 3.18639 9.69146 12.47712 Biso 1.000 O
O54 1.0000 4.76929 11.63011 11.35784 Biso 1.000 O
O55 1.0000 0.02059 11.63011 11.35784 Biso 1.000 O
Ce30 1.0000 2.37479 9.69948 14.67959 Biso 1.000 Ce
O56 1.0000 1.58444 11.62521 13.56250 Biso 1.000 O
O57 1.0000 6.32020 11.63032 13.57662 Biso 1.000 O
O58 1.0000 4.81751 9.69375 14.80125 Biso 1.000 O
Ce31 1.0000 5.56211 11.63857 15.86532 Biso 1.000 Ce
Ce32 1.0000 2.31454 11.63657 18.00864 Biso 1.000 Ce
O59 1.0000 1.59940 9.70503 16.87974 Biso 1.000 O
O60 1.0000 6.46980 9.68907 17.22642 Biso 1.000 O
O61 1.0000 7.89967 11.61329 15.79286 Biso 1.000 O
O62 1.0000 3.11873 11.64736 15.77429 Biso 1.000 O
Ce33 1.0000 5.28596 9.70152 19.28015 Biso 1.000 Ce
O63 1.0000 4.58799 11.60481 17.93575 Biso 1.000 O
O64 1.0000 0.00140 11.61306 18.01952 Biso 1.000 O
O65 1.0000 3.03367 9.66874 19.17405 Biso 1.000 O
O66 1.0000 5.86834 11.62355 20.21061 Biso 1.000 O
Rh1 1.0000 7.72629 3.94608 19.10868 Biso 1.000 Rh
H1 1.0000 8.37860 2.95054 20.11258 Biso 1.000 H

RhH2allyl_1x3-CeO2-211
data_image0
loop_
_atom_site_label
_atom_site_occupancy
_atom_site_Cartn_x
_atom_site_Cartn_y

_atom_site_Cartn_z
_atom_site_thermal_displace_type
_atom_site_B_iso_or_equiv
_atom_site_type_symbol
Ce1 1.0000 -0.77087 1.93685 10.23856 Biso 1.000 Ce
Ce2 1.0000 -0.75864 1.90972 16.95176 Biso 1.000 Ce
O1 1.0000 0.02059 1.93685 8.00000 Biso 1.000 O
O2 1.0000 1.60349 1.93685 10.23856 Biso 1.000 O
O3 1.0000 0.00391 1.93474 14.72303 Biso 1.000 O
Ce3 1.0000 5.56074 3.87550 9.11928 Biso 1.000 Ce
Ce4 1.0000 2.39494 1.93685 8.00000 Biso 1.000 Ce
O4 1.0000 4.76929 1.93685 8.00000 Biso 1.000 O
O5 1.0000 7.93510 3.87550 9.11928 Biso 1.000 O
O6 1.0000 3.18639 3.87550 9.11928 Biso 1.000 O
O7 1.0000 6.35219 1.93685 10.23856 Biso 1.000 O
Ce5 1.0000 2.39494 3.87550 11.35784 Biso 1.000 Ce
Ce6 1.0000 -0.78306 3.87382 13.57599 Biso 1.000 Ce
Ce7 1.0000 5.56074 1.93685 12.47712 Biso 1.000 Ce
O8 1.0000 7.93510 1.93685 12.47712 Biso 1.000 O
O9 1.0000 3.18639 1.93685 12.47712 Biso 1.000 O
O10 1.0000 4.76929 3.87550 11.35784 Biso 1.000 O
O11 1.0000 0.02059 3.87550 11.35784 Biso 1.000 O
Ce8 1.0000 2.38298 1.93744 14.69878 Biso 1.000 Ce
O12 1.0000 1.59411 3.87596 13.57021 Biso 1.000 O
O13 1.0000 6.32020 3.87571 13.57662 Biso 1.000 O
O14 1.0000 4.79704 1.94151 14.76206 Biso 1.000 O
Ce9 1.0000 5.56720 3.87169 15.86269 Biso 1.000 Ce
Ce10 1.0000 2.40528 3.86937 18.03550 Biso 1.000 Ce
O15 1.0000 1.57230 1.96069 16.91381 Biso 1.000 O
O16 1.0000 6.37730 1.85771 17.06960 Biso 1.000 O
O17 1.0000 7.92227 3.87703 15.83253 Biso 1.000 O
O18 1.0000 3.16070 3.87208 15.80463 Biso 1.000 O
Ce11 1.0000 5.37886 1.87262 19.23631 Biso 1.000 Ce
O19 1.0000 4.67930 3.88217 17.98023 Biso 1.000 O
O20 1.0000 0.07568 3.89554 18.18256 Biso 1.000 O
O21 1.0000 3.09177 1.94069 19.18629 Biso 1.000 O
O22 1.0000 6.05010 3.85420 20.31531 Biso 1.000 O
Ce12 1.0000 -0.77087 5.81416 10.23856 Biso 1.000 Ce
Ce13 1.0000 -0.74748 5.83440 16.93173 Biso 1.000 Ce
O23 1.0000 0.02059 5.81416 8.00000 Biso 1.000 O
O24 1.0000 1.60349 5.81416 10.23856 Biso 1.000 O
O25 1.0000 0.00640 5.81461 14.72534 Biso 1.000 O
Ce14 1.0000 5.56074 7.75281 9.11928 Biso 1.000 Ce
Ce15 1.0000 2.39494 5.81416 8.00000 Biso 1.000 Ce
O26 1.0000 4.76929 5.81416 8.00000 Biso 1.000 O
O27 1.0000 7.93510 7.75281 9.11928 Biso 1.000 O
O28 1.0000 3.18639 7.75281 9.11928 Biso 1.000 O
O29 1.0000 6.35219 5.81416 10.23856 Biso 1.000 O
Ce16 1.0000 2.39494 7.75281 11.35784 Biso 1.000 Ce
Ce17 1.0000 -0.78327 7.75136 13.56349 Biso 1.000 Ce
Ce18 1.0000 5.56074 5.81416 12.47712 Biso 1.000 Ce
O30 1.0000 7.93510 5.81416 12.47712 Biso 1.000 O
O31 1.0000 3.18639 5.81416 12.47712 Biso 1.000 O
O32 1.0000 4.76929 7.75281 11.35784 Biso 1.000 O
O33 1.0000 0.02059 7.75281 11.35784 Biso 1.000 O
Ce19 1.0000 2.38988 5.81330 14.69847 Biso 1.000 Ce
O34 1.0000 1.59284 7.74917 13.55473 Biso 1.000 O
O35 1.0000 6.32020 7.75301 13.57662 Biso 1.000 O
O36 1.0000 4.79848 5.80745 14.76470 Biso 1.000 O
Ce20 1.0000 5.56515 7.75538 15.86207 Biso 1.000 Ce

Ce21 1.0000 2.32816 7.74842 18.01385 Biso 1.000 Ce
O37 1.0000 1.58404 5.79466 16.90947 Biso 1.000 O
O38 1.0000 6.38406 5.92375 17.07355 Biso 1.000 O
O39 1.0000 7.92124 7.77041 15.79077 Biso 1.000 O
O40 1.0000 3.11956 7.74439 15.77416 Biso 1.000 O
Ce22 1.0000 5.36578 5.85674 19.22754 Biso 1.000 Ce
O41 1.0000 4.58770 7.77828 17.93410 Biso 1.000 O
O42 1.0000 0.00891 7.75739 18.03200 Biso 1.000 O
O43 1.0000 3.08899 5.80076 19.19970 Biso 1.000 O
O44 1.0000 5.84118 7.74548 20.20992 Biso 1.000 O
Ce23 1.0000 -0.77087 9.69146 10.23856 Biso 1.000 Ce
Ce24 1.0000 -0.72537 9.69187 16.89774 Biso 1.000 Ce
O45 1.0000 0.02059 9.69146 8.00000 Biso 1.000 O
O46 1.0000 1.60349 9.69146 10.23856 Biso 1.000 O
O47 1.0000 0.02353 9.69500 14.70564 Biso 1.000 O
Ce25 1.0000 5.56074 11.63011 9.11928 Biso 1.000 Ce
Ce26 1.0000 2.39494 9.69146 8.00000 Biso 1.000 Ce
O48 1.0000 4.76929 9.69146 8.00000 Biso 1.000 O
O49 1.0000 7.93510 11.63011 9.11928 Biso 1.000 O
O50 1.0000 3.18639 11.63011 9.11928 Biso 1.000 O
O51 1.0000 6.35219 9.69146 10.23856 Biso 1.000 O
Ce27 1.0000 2.39494 11.63011 11.35784 Biso 1.000 Ce
Ce28 1.0000 -0.78389 11.63412 13.56576 Biso 1.000 Ce
Ce29 1.0000 5.56074 9.69146 12.47712 Biso 1.000 Ce
O52 1.0000 7.93510 9.69146 12.47712 Biso 1.000 O
O53 1.0000 3.18639 9.69146 12.47712 Biso 1.000 O
O54 1.0000 4.76929 11.63011 11.35784 Biso 1.000 O
O55 1.0000 0.02059 11.63011 11.35784 Biso 1.000 O
Ce30 1.0000 2.38875 9.69237 14.69060 Biso 1.000 Ce
O56 1.0000 1.59373 11.63419 13.55425 Biso 1.000 O
O57 1.0000 6.32020 11.63032 13.57662 Biso 1.000 O
O58 1.0000 4.80983 9.69438 14.79132 Biso 1.000 O
Ce31 1.0000 5.56215 11.63322 15.86230 Biso 1.000 Ce
Ce32 1.0000 2.32538 11.63572 18.01819 Biso 1.000 Ce
O59 1.0000 1.60070 9.68850 16.89375 Biso 1.000 O
O60 1.0000 6.48227 9.68518 17.23545 Biso 1.000 O
O61 1.0000 7.91054 11.61567 15.79774 Biso 1.000 O
O62 1.0000 3.11514 11.64243 15.77900 Biso 1.000 O
Ce33 1.0000 5.27311 9.68660 19.26576 Biso 1.000 Ce
O63 1.0000 4.59473 11.59503 17.93270 Biso 1.000 O
O64 1.0000 0.01140 11.61861 18.02246 Biso 1.000 O
O65 1.0000 3.01543 9.68970 19.20441 Biso 1.000 O
O66 1.0000 5.85134 11.61374 20.21275 Biso 1.000 O
Rh1 1.0000 7.96031 3.85365 19.49991 Biso 1.000 Rh
H1 1.0000 7.84657 2.21618 19.24980 Biso 1.000 H
C1 1.0000 9.26826 4.69036 21.04693 Biso 1.000 C
C2 1.0000 8.40863 5.74473 20.59787 Biso 1.000 C
H2 1.0000 10.32336 4.73326 20.74978 Biso 1.000 H
H3 1.0000 8.86194 6.61426 20.11411 Biso 1.000 H
H4 1.0000 7.52426 5.98505 21.19680 Biso 1.000 H
C3 1.0000 8.78364 3.51388 21.62243 Biso 1.000 C
H5 1.0000 9.46750 2.68727 21.81806 Biso 1.000 H
H6 1.0000 7.81337 3.48842 22.12237 Biso 1.000 H
H7 1.0000 7.15603 4.13231 18.17257 Biso 1.000 H

RhH2SiMe3_1x3-CeO2-211
data_image0
loop_
_atom_site_label

_atom_site_occupancy
_atom_site_Cartn_x
_atom_site_Cartn_y
_atom_site_Cartn_z
_atom_site_thermal_displace_type
_atom_site_B_iso_or_equiv
_atom_site_type_symbol
Ce1 1.0000 -0.77087 1.93685 10.23856 Biso 1.000 Ce
Ce2 1.0000 -0.75574 1.89164 16.92640 Biso 1.000 Ce
O1 1.0000 0.02059 1.93685 8.00000 Biso 1.000 O
O2 1.0000 1.60349 1.93685 10.23856 Biso 1.000 O
O3 1.0000 0.01453 1.92632 14.71981 Biso 1.000 O
Ce3 1.0000 5.56074 3.87550 9.11928 Biso 1.000 Ce
Ce4 1.0000 2.39494 1.93685 8.00000 Biso 1.000 Ce
O4 1.0000 4.76929 1.93685 8.00000 Biso 1.000 O
O5 1.0000 7.93510 3.87550 9.11928 Biso 1.000 O
O6 1.0000 3.18639 3.87550 9.11928 Biso 1.000 O
O7 1.0000 6.35219 1.93685 10.23856 Biso 1.000 O
Ce5 1.0000 2.39494 3.87550 11.35784 Biso 1.000 Ce
Ce6 1.0000 -0.78868 3.87246 13.57194 Biso 1.000 Ce
Ce7 1.0000 5.56074 1.93685 12.47712 Biso 1.000 Ce
O8 1.0000 7.93510 1.93685 12.47712 Biso 1.000 O
O9 1.0000 3.18639 1.93685 12.47712 Biso 1.000 O
O10 1.0000 4.76929 3.87550 11.35784 Biso 1.000 O
O11 1.0000 0.02059 3.87550 11.35784 Biso 1.000 O
Ce8 1.0000 2.38307 1.94832 14.70242 Biso 1.000 Ce
O12 1.0000 1.59340 3.87324 13.56914 Biso 1.000 O
O13 1.0000 6.32020 3.87571 13.57662 Biso 1.000 O
O14 1.0000 4.80724 1.93973 14.78039 Biso 1.000 O
Ce9 1.0000 5.56526 3.87016 15.88059 Biso 1.000 Ce
Ce10 1.0000 2.34909 3.89910 18.06469 Biso 1.000 Ce
O15 1.0000 1.54581 2.00084 16.95276 Biso 1.000 O
O16 1.0000 6.39633 1.90899 17.12195 Biso 1.000 O
O17 1.0000 7.90730 3.86239 15.82301 Biso 1.000 O
O18 1.0000 3.14426 3.87145 15.79977 Biso 1.000 O
Ce11 1.0000 5.36751 1.87470 19.20838 Biso 1.000 Ce
O19 1.0000 4.61061 3.87776 17.98015 Biso 1.000 O
O20 1.0000 -0.08320 4.09895 18.18769 Biso 1.000 O
O21 1.0000 3.09598 1.93154 19.20334 Biso 1.000 O
O22 1.0000 6.40178 3.95776 19.91432 Biso 1.000 O
Ce12 1.0000 -0.77087 5.81416 10.23856 Biso 1.000 Ce
Ce13 1.0000 -0.73003 5.85229 16.90221 Biso 1.000 Ce
O23 1.0000 0.02059 5.81416 8.00000 Biso 1.000 O
O24 1.0000 1.60349 5.81416 10.23856 Biso 1.000 O
O25 1.0000 0.01815 5.81439 14.71146 Biso 1.000 O
Ce14 1.0000 5.56074 7.75281 9.11928 Biso 1.000 Ce
Ce15 1.0000 2.39494 5.81416 8.00000 Biso 1.000 Ce
O26 1.0000 4.76929 5.81416 8.00000 Biso 1.000 O
O27 1.0000 7.93510 7.75281 9.11928 Biso 1.000 O
O28 1.0000 3.18639 7.75281 9.11928 Biso 1.000 O
O29 1.0000 6.35219 5.81416 10.23856 Biso 1.000 O
Ce16 1.0000 2.39494 7.75281 11.35784 Biso 1.000 Ce
Ce17 1.0000 -0.78105 7.75121 13.55177 Biso 1.000 Ce
Ce18 1.0000 5.56074 5.81416 12.47712 Biso 1.000 Ce
O30 1.0000 7.93510 5.81416 12.47712 Biso 1.000 O
O31 1.0000 3.18639 5.81416 12.47712 Biso 1.000 O
O32 1.0000 4.76929 7.75281 11.35784 Biso 1.000 O
O33 1.0000 0.02059 7.75281 11.35784 Biso 1.000 O
Ce19 1.0000 2.39777 5.81038 14.69533 Biso 1.000 Ce
O34 1.0000 1.59416 7.75252 13.55741 Biso 1.000 O

O35	1.0000	6.32020	7.75301	13.57662	Biso	1.000	O
O36	1.0000	4.82335	5.80910	14.79532	Biso	1.000	O
Ce20	1.0000	5.56573	7.75548	15.86486	Biso	1.000	Ce
Ce21	1.0000	2.33197	7.75160	18.01563	Biso	1.000	Ce
O37	1.0000	1.61134	5.80327	16.90306	Biso	1.000	O
O38	1.0000	6.44461	5.84783	17.17191	Biso	1.000	O
O39	1.0000	7.90079	7.76352	15.77912	Biso	1.000	O
O40	1.0000	3.13889	7.74407	15.76386	Biso	1.000	O
Ce22	1.0000	5.36807	5.86135	19.22431	Biso	1.000	Ce
O41	1.0000	4.60132	7.76032	17.92814	Biso	1.000	O
O42	1.0000	0.01631	7.76618	18.02229	Biso	1.000	O
O43	1.0000	3.07707	5.80978	19.21181	Biso	1.000	O
O44	1.0000	5.91802	7.74652	20.17906	Biso	1.000	O
Ce23	1.0000	-0.77087	9.69146	10.23856	Biso	1.000	Ce
Ce24	1.0000	-0.71689	9.68239	16.89248	Biso	1.000	Ce
O45	1.0000	0.02059	9.69146	8.00000	Biso	1.000	O
O46	1.0000	1.60349	9.69146	10.23856	Biso	1.000	O
O47	1.0000	0.02238	9.69430	14.70645	Biso	1.000	O
Ce25	1.0000	5.56074	11.63011	9.11928	Biso	1.000	Ce
Ce26	1.0000	2.39494	9.69146	8.00000	Biso	1.000	Ce
O48	1.0000	4.76929	9.69146	8.00000	Biso	1.000	O
O49	1.0000	7.93510	11.63011	9.11928	Biso	1.000	O
O50	1.0000	3.18639	11.63011	9.11928	Biso	1.000	O
O51	1.0000	6.35219	9.69146	10.23856	Biso	1.000	O
Ce27	1.0000	2.39494	11.63011	11.35784	Biso	1.000	Ce
Ce28	1.0000	-0.78189	11.63203	13.55804	Biso	1.000	Ce
Ce29	1.0000	5.56074	9.69146	12.47712	Biso	1.000	Ce
O52	1.0000	7.93510	9.69146	12.47712	Biso	1.000	O
O53	1.0000	3.18639	9.69146	12.47712	Biso	1.000	O
O54	1.0000	4.76929	11.63011	11.35784	Biso	1.000	O
O55	1.0000	0.02059	11.63011	11.35784	Biso	1.000	O
Ce30	1.0000	2.39062	9.69244	14.68789	Biso	1.000	Ce
O56	1.0000	1.59608	11.63290	13.55743	Biso	1.000	O
O57	1.0000	6.32020	11.63032	13.57662	Biso	1.000	O
O58	1.0000	4.81502	9.69847	14.80175	Biso	1.000	O
Ce31	1.0000	5.56241	11.63382	15.85739	Biso	1.000	Ce
Ce32	1.0000	2.32878	11.63204	18.01949	Biso	1.000	Ce
O59	1.0000	1.62689	9.67300	16.88498	Biso	1.000	O
O60	1.0000	6.48051	9.68380	17.23238	Biso	1.000	O
O61	1.0000	7.88876	11.62456	15.79117	Biso	1.000	O
O62	1.0000	3.10328	11.65862	15.78927	Biso	1.000	O
Ce33	1.0000	5.29563	9.68605	19.26403	Biso	1.000	Ce
O63	1.0000	4.61073	11.61558	17.92326	Biso	1.000	O
O64	1.0000	0.03350	11.55380	17.96951	Biso	1.000	O
O65	1.0000	3.03789	9.69385	19.19508	Biso	1.000	O
O66	1.0000	5.83320	11.60158	20.22439	Biso	1.000	O
Rh1	1.0000	8.27083	2.95140	19.64699	Biso	1.000	Rh
Si1	1.0000	9.20840	2.04563	21.58382	Biso	1.000	Si
C1	1.0000	9.88560	0.28704	21.51821	Biso	1.000	C
H1	1.0000	10.02241	-0.11738	22.53340	Biso	1.000	H
H2	1.0000	10.87179	0.26992	21.02673	Biso	1.000	H
H3	1.0000	9.20802	-0.38115	20.96799	Biso	1.000	H
C2	1.0000	10.63746	3.23359	21.96173	Biso	1.000	C
H4	1.0000	10.35991	4.29017	21.83528	Biso	1.000	H
H5	1.0000	11.48926	3.00653	21.30129	Biso	1.000	H
H6	1.0000	10.96876	3.09414	23.00495	Biso	1.000	H
C3	1.0000	7.91341	2.14458	22.95851	Biso	1.000	C
H7	1.0000	7.42140	3.12819	22.97061	Biso	1.000	H
H8	1.0000	8.38389	1.97480	23.94137	Biso	1.000	H
H9	1.0000	7.14139	1.37543	22.80263	Biso	1.000	H

```
H10      1.0000 9.53110  1.99885  19.48141  Biso   1.000  H
H11      1.0000 7.61997  1.65909  20.28214  Biso   1.000  H
```

RhH2SiMe3propen_1x3-CeO2-211

```
data_image0
loop_
  _atom_site_label
  _atom_site_occupancy
  _atom_site_Cartn_x
  _atom_site_Cartn_y
  _atom_site_Cartn_z
  _atom_site_thermal_displace_type
  _atom_site_B_iso_or_equiv
  _atom_site_type_symbol
  Ce1      1.0000 -0.77087  1.93685  10.23856  Biso   1.000  Ce
  Ce2      1.0000 -0.73967  1.92536  16.89761  Biso   1.000  Ce
  O1       1.0000 0.02059  1.93685  8.00000  Biso   1.000  O
  O2       1.0000 1.60349  1.93685  10.23856  Biso   1.000  O
  O3       1.0000 0.01934  1.93428  14.72713  Biso   1.000  O
  Ce3      1.0000 5.56074  3.87550  9.11928  Biso   1.000  Ce
  Ce4      1.0000 2.39494  1.93685  8.00000  Biso   1.000  Ce
  O4       1.0000 4.76929  1.93685  8.00000  Biso   1.000  O
  O5       1.0000 7.93510  3.87550  9.11928  Biso   1.000  O
  O6       1.0000 3.18639  3.87550  9.11928  Biso   1.000  O
  O7       1.0000 6.35219  1.93685  10.23856  Biso   1.000  O
  Ce5      1.0000 2.39494  3.87550  11.35784  Biso   1.000  Ce
  Ce6      1.0000 -0.78806  3.87493  13.54953  Biso   1.000  Ce
  Ce7      1.0000 5.56074  1.93685  12.47712  Biso   1.000  Ce
  O8       1.0000 7.93510  1.93685  12.47712  Biso   1.000  O
  O9       1.0000 3.18639  1.93685  12.47712  Biso   1.000  O
  O10      1.0000 4.76929  3.87550  11.35784  Biso   1.000  O
  O11      1.0000 0.02059  3.87550  11.35784  Biso   1.000  O
  Ce8      1.0000 2.39058  1.94732  14.69380  Biso   1.000  Ce
  O12      1.0000 1.59392  3.87460  13.57937  Biso   1.000  O
  O13      1.0000 6.32020  3.87571  13.57662  Biso   1.000  O
  O14      1.0000 4.82507  1.93851  14.80637  Biso   1.000  O
  Ce9      1.0000 5.56447  3.87376  15.87334  Biso   1.000  Ce
  Ce10     1.0000 2.34457  3.87843  18.07691  Biso   1.000  Ce
  O15      1.0000 1.57256  1.99140  16.94580  Biso   1.000  O
  O16      1.0000 6.44830  1.91485  17.18341  Biso   1.000  O
  O17      1.0000 7.89912  3.86716  15.79823  Biso   1.000  O
  O18      1.0000 3.15374  3.87110  15.80155  Biso   1.000  O
  Ce11     1.0000 5.34602  1.89996  19.21452  Biso   1.000  Ce
  O19      1.0000 4.60498  3.86624  17.99014  Biso   1.000  O
  O20      1.0000 -0.15055  3.89671  18.14483  Biso   1.000  O
  O21      1.0000 3.07257  1.93489  19.22513  Biso   1.000  O
  O22      1.0000 6.37553  3.84117  19.94328  Biso   1.000  O
  Ce12     1.0000 -0.77087  5.81416  10.23856  Biso   1.000  Ce
  Ce13     1.0000 -0.73608  5.82941  16.89504  Biso   1.000  Ce
  O23      1.0000 0.02059  5.81416  8.00000  Biso   1.000  O
  O24      1.0000 1.60349  5.81416  10.23856  Biso   1.000  O
  O25      1.0000 0.02000  5.81496  14.72391  Biso   1.000  O
  Ce14     1.0000 5.56074  7.75281  9.11928  Biso   1.000  Ce
  Ce15     1.0000 2.39494  5.81416  8.00000  Biso   1.000  Ce
  O26      1.0000 4.76929  5.81416  8.00000  Biso   1.000  O
  O27      1.0000 7.93510  7.75281  9.11928  Biso   1.000  O
  O28      1.0000 3.18639  7.75281  9.11928  Biso   1.000  O
  O29      1.0000 6.35219  5.81416  10.23856  Biso   1.000  O
  Ce16     1.0000 2.39494  7.75281  11.35784  Biso   1.000  Ce
```

Ce17	1.0000	-0.78103	7.75066	13.54147	Biso	1.000	Ce
Ce18	1.0000	5.56074	5.81416	12.47712	Biso	1.000	Ce
O30	1.0000	7.93510	5.81416	12.47712	Biso	1.000	O
O31	1.0000	3.18639	5.81416	12.47712	Biso	1.000	O
O32	1.0000	4.76929	7.75281	11.35784	Biso	1.000	O
O33	1.0000	0.02059	7.75281	11.35784	Biso	1.000	O
Ce19	1.0000	2.39299	5.80157	14.69317	Biso	1.000	Ce
O34	1.0000	1.59427	7.74993	13.57009	Biso	1.000	O
O35	1.0000	6.32020	7.75301	13.57662	Biso	1.000	O
O36	1.0000	4.82439	5.80843	14.80555	Biso	1.000	O
Ce20	1.0000	5.57342	7.75146	15.85746	Biso	1.000	Ce
Ce21	1.0000	2.35146	7.74600	17.99818	Biso	1.000	Ce
O37	1.0000	1.59226	5.75875	16.94022	Biso	1.000	O
O38	1.0000	6.44519	5.82756	17.18358	Biso	1.000	O
O39	1.0000	7.89063	7.75796	15.78219	Biso	1.000	O
O40	1.0000	3.12585	7.72879	15.78001	Biso	1.000	O
Ce22	1.0000	5.37890	5.83830	19.22443	Biso	1.000	Ce
O41	1.0000	4.62199	7.76111	17.92447	Biso	1.000	O
O42	1.0000	0.03949	7.77114	17.96284	Biso	1.000	O
O43	1.0000	3.10723	5.81293	19.21024	Biso	1.000	O
O44	1.0000	5.87041	7.74392	20.20287	Biso	1.000	O
Ce23	1.0000	-0.77087	9.69146	10.23856	Biso	1.000	Ce
Ce24	1.0000	-0.71057	9.69052	16.87652	Biso	1.000	Ce
O45	1.0000	0.02059	9.69146	8.00000	Biso	1.000	O
O46	1.0000	1.60349	9.69146	10.23856	Biso	1.000	O
O47	1.0000	0.02212	9.69243	14.70851	Biso	1.000	O
Ce25	1.0000	5.56074	11.63011	9.11928	Biso	1.000	Ce
Ce26	1.0000	2.39494	9.69146	8.00000	Biso	1.000	Ce
O48	1.0000	4.76929	9.69146	8.00000	Biso	1.000	O
O49	1.0000	7.93510	11.63011	9.11928	Biso	1.000	O
O50	1.0000	3.18639	11.63011	9.11928	Biso	1.000	O
O51	1.0000	6.35219	9.69146	10.23856	Biso	1.000	O
Ce27	1.0000	2.39494	11.63011	11.35784	Biso	1.000	Ce
Ce28	1.0000	-0.78110	11.63231	13.54166	Biso	1.000	Ce
Ce29	1.0000	5.56074	9.69146	12.47712	Biso	1.000	Ce
O52	1.0000	7.93510	9.69146	12.47712	Biso	1.000	O
O53	1.0000	3.18639	9.69146	12.47712	Biso	1.000	O
O54	1.0000	4.76929	11.63011	11.35784	Biso	1.000	O
O55	1.0000	0.02059	11.63011	11.35784	Biso	1.000	O
Ce30	1.0000	2.39133	9.69137	14.67783	Biso	1.000	Ce
O56	1.0000	1.59358	11.63300	13.56879	Biso	1.000	O
O57	1.0000	6.32020	11.63032	13.57662	Biso	1.000	O
O58	1.0000	4.81699	9.69287	14.81664	Biso	1.000	O
Ce31	1.0000	5.57352	11.62957	15.85792	Biso	1.000	Ce
Ce32	1.0000	2.33401	11.62750	17.99958	Biso	1.000	Ce
O59	1.0000	1.63325	9.68817	16.88485	Biso	1.000	O
O60	1.0000	6.49053	9.69509	17.23999	Biso	1.000	O
O61	1.0000	7.89444	11.63098	15.78512	Biso	1.000	O
O62	1.0000	3.11875	11.65463	15.78106	Biso	1.000	O
Ce33	1.0000	5.30202	9.69156	19.25420	Biso	1.000	Ce
O63	1.0000	4.60550	11.62389	17.92581	Biso	1.000	O
O64	1.0000	0.03044	11.59522	17.97683	Biso	1.000	O
O65	1.0000	3.05208	9.68939	19.19377	Biso	1.000	O
O66	1.0000	5.91383	11.63857	20.17636	Biso	1.000	O
Rh1	1.0000	8.63114	3.90858	20.23216	Biso	1.000	Rh
Si1	1.0000	9.28184	5.75687	21.89298	Biso	1.000	Si
C1	1.0000	11.10599	5.51909	22.31938	Biso	1.000	C
H1	1.0000	11.39237	6.29738	23.04719	Biso	1.000	H
H2	1.0000	11.73180	5.63271	21.42136	Biso	1.000	H
H3	1.0000	11.31250	4.53935	22.77102	Biso	1.000	H

```
 C2       1.0000 9.03129  7.44431  21.11814  Biso   1.000  C
 H4       1.0000 7.95836  7.68738  21.06345  Biso   1.000  H
 H5       1.0000 9.43012  7.53597  20.10068  Biso   1.000  H
 H6       1.0000 9.53297  8.18272  21.76838  Biso   1.000  H
 C3       1.0000 8.34803  5.85288  23.55118  Biso   1.000  C
 H7       1.0000 7.28684  6.09522  23.38813  Biso   1.000  H
 H8       1.0000 8.79158  6.66474  24.15188  Biso   1.000  H
 H9       1.0000 8.39976  4.92847  24.14414  Biso   1.000  H
 H10      1.0000 10.16483  3.97301  20.50790  Biso   1.000  H
 C4       1.0000 8.53578  2.23874  21.68405  Biso   1.000  C
 C5       1.0000 8.90498  1.70273  20.43929  Biso   1.000  C
 H11      1.0000 7.48007  2.15933  21.96695  Biso   1.000  H
 H12      1.0000 8.18493  1.11313  19.86439  Biso   1.000  H
 H13      1.0000 9.95278  1.45142  20.25912  Biso   1.000  H
 C6       1.0000 9.50526  2.25575  22.83529  Biso   1.000  C
 H14      1.0000 9.40420  1.31098  23.39745  Biso   1.000  H
 H15      1.0000 9.31888  3.07445  23.54297  Biso   1.000  H
 H16      1.0000 10.54328  2.32599  22.48042  Biso   1.000  H
 H17      1.0000 8.05823  4.53580  21.58968  Biso   1.000  H


RhHpropen_1x3-CeO2-211
data_image0
loop_
 _atom_site_label
 _atom_site_occupancy
 _atom_site_Cartn_x
 _atom_site_Cartn_y
 _atom_site_Cartn_z
 _atom_site_thermal_displace_type
 _atom_site_B_iso_or_equiv
 _atom_site_type_symbol
 Ce1      1.0000 -0.77087  1.93685  10.23856  Biso   1.000  Ce
 Ce2      1.0000 -0.75840  1.90745  16.92774  Biso   1.000  Ce
 O1       1.0000 0.02059  1.93685  8.00000  Biso   1.000  O
 O2       1.0000 1.60349  1.93685  10.23856  Biso   1.000  O
 O3       1.0000 0.00403  1.93680  14.71260  Biso   1.000  O
 Ce3      1.0000 5.56074  3.87550  9.11928  Biso   1.000  Ce
 Ce4      1.0000 2.39494  1.93685  8.00000  Biso   1.000  Ce
 O4       1.0000 4.76929  1.93685  8.00000  Biso   1.000  O
 O5       1.0000 7.93510  3.87550  9.11928  Biso   1.000  O
 O6       1.0000 3.18639  3.87550  9.11928  Biso   1.000  O
 O7       1.0000 6.35219  1.93685  10.23856  Biso   1.000  O
 Ce5      1.0000 2.39494  3.87550  11.35784  Biso   1.000  Ce
 Ce6      1.0000 -0.78539  3.87389  13.56628  Biso   1.000  Ce
 Ce7      1.0000 5.56074  1.93685  12.47712  Biso   1.000  Ce
 O8       1.0000 7.93510  1.93685  12.47712  Biso   1.000  O
 O9       1.0000 3.18639  1.93685  12.47712  Biso   1.000  O
 O10      1.0000 4.76929  3.87550  11.35784  Biso   1.000  O
 O11      1.0000 0.02059  3.87550  11.35784  Biso   1.000  O
 Ce8      1.0000 2.38364  1.94136  14.68934  Biso   1.000  Ce
 O12      1.0000 1.59309  3.87913  13.57461  Biso   1.000  O
 O13      1.0000 6.32020  3.87571  13.57662  Biso   1.000  O
 O14      1.0000 4.79691  1.94245  14.76390  Biso   1.000  O
 Ce9      1.0000 5.56738  3.86931  15.87199  Biso   1.000  Ce
 Ce10     1.0000 2.39649  3.87522  18.02762  Biso   1.000  Ce
 O15      1.0000 1.56793  1.97208  16.90741  Biso   1.000  O
 O16      1.0000 6.36575  1.88142  17.06983  Biso   1.000  O
 O17      1.0000 7.91740  3.87390  15.85078  Biso   1.000  O
 O18      1.0000 3.16231  3.87618  15.80124  Biso   1.000  O
```

Ce11	1.0000	5.35514	1.86187	19.22203	Biso	1.000	Ce
O19	1.0000	4.66968	3.88487	17.99160	Biso	1.000	O
O20	1.0000	0.04088	3.91003	18.19540	Biso	1.000	O
O21	1.0000	3.07550	1.93789	19.18170	Biso	1.000	O
O22	1.0000	6.09811	3.87778	20.24973	Biso	1.000	O
Ce12	1.0000	-0.77087	5.81416	10.23856	Biso	1.000	Ce
Ce13	1.0000	-0.74517	5.84285	16.91727	Biso	1.000	Ce
O23	1.0000	0.02059	5.81416	8.00000	Biso	1.000	O
O24	1.0000	1.60349	5.81416	10.23856	Biso	1.000	O
O25	1.0000	0.00506	5.81402	14.72159	Biso	1.000	O
Ce14	1.0000	5.56074	7.75281	9.11928	Biso	1.000	Ce
Ce15	1.0000	2.39494	5.81416	8.00000	Biso	1.000	Ce
O26	1.0000	4.76929	5.81416	8.00000	Biso	1.000	O
O27	1.0000	7.93510	7.75281	9.11928	Biso	1.000	O
O28	1.0000	3.18639	7.75281	9.11928	Biso	1.000	O
O29	1.0000	6.35219	5.81416	10.23856	Biso	1.000	O
Ce16	1.0000	2.39494	7.75281	11.35784	Biso	1.000	Ce
Ce17	1.0000	-0.78427	7.75152	13.55218	Biso	1.000	Ce
Ce18	1.0000	5.56074	5.81416	12.47712	Biso	1.000	Ce
O30	1.0000	7.93510	5.81416	12.47712	Biso	1.000	O
O31	1.0000	3.18639	5.81416	12.47712	Biso	1.000	O
O32	1.0000	4.76929	7.75281	11.35784	Biso	1.000	O
O33	1.0000	0.02059	7.75281	11.35784	Biso	1.000	O
Ce19	1.0000	2.38976	5.81449	14.69377	Biso	1.000	Ce
O34	1.0000	1.59163	7.74884	13.56045	Biso	1.000	O
O35	1.0000	6.32020	7.75301	13.57662	Biso	1.000	O
O36	1.0000	4.81607	5.81356	14.79158	Biso	1.000	O
Ce20	1.0000	5.57071	7.75333	15.86237	Biso	1.000	Ce
Ce21	1.0000	2.32510	7.76058	17.99882	Biso	1.000	Ce
O37	1.0000	1.58605	5.79635	16.90593	Biso	1.000	O
O38	1.0000	6.42551	5.87027	17.15876	Biso	1.000	O
O39	1.0000	7.90329	7.76252	15.78907	Biso	1.000	O
O40	1.0000	3.12312	7.74716	15.77264	Biso	1.000	O
Ce22	1.0000	5.33571	5.89668	19.23831	Biso	1.000	Ce
O41	1.0000	4.59904	7.77181	17.92769	Biso	1.000	O
O42	1.0000	0.01883	7.78429	18.00988	Biso	1.000	O
O43	1.0000	3.07372	5.81787	19.19768	Biso	1.000	O
O44	1.0000	5.78284	7.79081	20.24571	Biso	1.000	O
Ce23	1.0000	-0.77087	9.69146	10.23856	Biso	1.000	Ce
Ce24	1.0000	-0.72898	9.69976	16.88678	Biso	1.000	Ce
O45	1.0000	0.02059	9.69146	8.00000	Biso	1.000	O
O46	1.0000	1.60349	9.69146	10.23856	Biso	1.000	O
O47	1.0000	0.01734	9.69239	14.70237	Biso	1.000	O
Ce25	1.0000	5.56074	11.63011	9.11928	Biso	1.000	Ce
Ce26	1.0000	2.39494	9.69146	8.00000	Biso	1.000	Ce
O48	1.0000	4.76929	9.69146	8.00000	Biso	1.000	O
O49	1.0000	7.93510	11.63011	9.11928	Biso	1.000	O
O50	1.0000	3.18639	11.63011	9.11928	Biso	1.000	O
O51	1.0000	6.35219	9.69146	10.23856	Biso	1.000	O
Ce27	1.0000	2.39494	11.63011	11.35784	Biso	1.000	Ce
Ce28	1.0000	-0.78386	11.63382	13.55279	Biso	1.000	Ce
Ce29	1.0000	5.56074	9.69146	12.47712	Biso	1.000	Ce
O52	1.0000	7.93510	9.69146	12.47712	Biso	1.000	O
O53	1.0000	3.18639	9.69146	12.47712	Biso	1.000	O
O54	1.0000	4.76929	11.63011	11.35784	Biso	1.000	O
O55	1.0000	0.02059	11.63011	11.35784	Biso	1.000	O
Ce30	1.0000	2.38755	9.69418	14.68162	Biso	1.000	Ce
O56	1.0000	1.59173	11.63320	13.55832	Biso	1.000	O
O57	1.0000	6.32020	11.63032	13.57662	Biso	1.000	O
O58	1.0000	4.81496	9.69012	14.80027	Biso	1.000	O

Ce31 1.0000 5.55804 11.63629 15.85111 Biso 1.000 Ce
Ce32 1.0000 2.31619 11.64112 18.00474 Biso 1.000 Ce
O59 1.0000 1.60152 9.69984 16.88000 Biso 1.000 O
O60 1.0000 6.48230 9.68922 17.23749 Biso 1.000 O
O61 1.0000 7.90315 11.62273 15.79098 Biso 1.000 O
O62 1.0000 3.11782 11.64735 15.77340 Biso 1.000 O
Ce33 1.0000 5.26442 9.70331 19.26378 Biso 1.000 Ce
O63 1.0000 4.59300 11.60553 17.92915 Biso 1.000 O
O64 1.0000 0.00794 11.62111 18.01306 Biso 1.000 O
O65 1.0000 3.00877 9.69579 19.18687 Biso 1.000 O
O66 1.0000 5.84180 11.62609 20.21897 Biso 1.000 O
Rh1 1.0000 7.93933 3.93461 19.46204 Biso 1.000 Rh
H1 1.0000 7.72973 2.31908 19.06720 Biso 1.000 H
C1 1.0000 9.12161 4.82588 20.96924 Biso 1.000 C
C2 1.0000 8.34979 5.88208 20.43533 Biso 1.000 C
H2 1.0000 10.16907 4.76250 20.64402 Biso 1.000 H
H3 1.0000 8.82515 6.64561 19.81096 Biso 1.000 H
H4 1.0000 7.50426 6.25879 21.02241 Biso 1.000 H
C3 1.0000 8.83102 4.15906 22.28305 Biso 1.000 C
H5 1.0000 9.43192 4.63759 23.07816 Biso 1.000 H
H6 1.0000 9.10728 3.09379 22.26371 Biso 1.000 H
H7 1.0000 7.76852 4.24131 22.54809 Biso 1.000 H

RhHSiMe3isopropyl_1x3-CeO2-211
data_image0
loop_
_atom_site_label
_atom_site_occupancy
_atom_site_Cartn_x
_atom_site_Cartn_y
_atom_site_Cartn_z
_atom_site_thermal_displace_type
_atom_site_B_iso_or_equiv
_atom_site_type_symbol
Ce1 1.0000 -0.77087 1.93685 10.23856 Biso 1.000 Ce
Ce2 1.0000 -0.73656 1.91528 16.86248 Biso 1.000 Ce
O1 1.0000 0.02059 1.93685 8.00000 Biso 1.000 O
O2 1.0000 1.60349 1.93685 10.23856 Biso 1.000 O
O3 1.0000 0.02217 1.93278 14.71226 Biso 1.000 O
Ce3 1.0000 5.56074 3.87550 9.11928 Biso 1.000 Ce
Ce4 1.0000 2.39494 1.93685 8.00000 Biso 1.000 Ce
O4 1.0000 4.76929 1.93685 8.00000 Biso 1.000 O
O5 1.0000 7.93510 3.87550 9.11928 Biso 1.000 O
O6 1.0000 3.18639 3.87550 9.11928 Biso 1.000 O
O7 1.0000 6.35219 1.93685 10.23856 Biso 1.000 O
Ce5 1.0000 2.39494 3.87550 11.35784 Biso 1.000 Ce
Ce6 1.0000 -0.78968 3.87615 13.54381 Biso 1.000 Ce
Ce7 1.0000 5.56074 1.93685 12.47712 Biso 1.000 Ce
O8 1.0000 7.93510 1.93685 12.47712 Biso 1.000 O
O9 1.0000 3.18639 1.93685 12.47712 Biso 1.000 O
O10 1.0000 4.76929 3.87550 11.35784 Biso 1.000 O
O11 1.0000 0.02059 3.87550 11.35784 Biso 1.000 O
Ce8 1.0000 2.39824 1.95159 14.69824 Biso 1.000 Ce
O12 1.0000 1.59829 3.87495 13.58411 Biso 1.000 O
O13 1.0000 6.32020 3.87571 13.57662 Biso 1.000 O
O14 1.0000 4.83086 1.94034 14.81515 Biso 1.000 O
Ce9 1.0000 5.56058 3.87518 15.89139 Biso 1.000 Ce
Ce10 1.0000 2.32731 3.88373 18.11072 Biso 1.000 Ce
O15 1.0000 1.56678 1.99743 16.94134 Biso 1.000 O

O16	1.0000	6.46133	1.91587	17.20819	Biso	1.000	O
O17	1.0000	7.90551	3.86465	15.80081	Biso	1.000	O
O18	1.0000	3.16436	3.87306	15.81474	Biso	1.000	O
Ce11	1.0000	5.34135	1.88698	19.23262	Biso	1.000	Ce
O19	1.0000	4.60740	3.86323	18.02298	Biso	1.000	O
O20	1.0000	-0.13919	3.91214	18.15713	Biso	1.000	O
O21	1.0000	3.07740	1.92031	19.22178	Biso	1.000	O
O22	1.0000	6.43933	3.86287	19.94979	Biso	1.000	O
Ce12	1.0000	-0.77087	5.81416	10.23856	Biso	1.000	Ce
Ce13	1.0000	-0.73418	5.84030	16.87305	Biso	1.000	Ce
O23	1.0000	0.02059	5.81416	8.00000	Biso	1.000	O
O24	1.0000	1.60349	5.81416	10.23856	Biso	1.000	O
O25	1.0000	0.02089	5.81586	14.71288	Biso	1.000	O
Ce14	1.0000	5.56074	7.75281	9.11928	Biso	1.000	Ce
Ce15	1.0000	2.39494	5.81416	8.00000	Biso	1.000	Ce
O26	1.0000	4.76929	5.81416	8.00000	Biso	1.000	O
O27	1.0000	7.93510	7.75281	9.11928	Biso	1.000	O
O28	1.0000	3.18639	7.75281	9.11928	Biso	1.000	O
O29	1.0000	6.35219	5.81416	10.23856	Biso	1.000	O
Ce16	1.0000	2.39494	7.75281	11.35784	Biso	1.000	Ce
Ce17	1.0000	-0.78096	7.75210	13.53786	Biso	1.000	Ce
Ce18	1.0000	5.56074	5.81416	12.47712	Biso	1.000	Ce
O30	1.0000	7.93510	5.81416	12.47712	Biso	1.000	O
O31	1.0000	3.18639	5.81416	12.47712	Biso	1.000	O
O32	1.0000	4.76929	7.75281	11.35784	Biso	1.000	O
O33	1.0000	0.02059	7.75281	11.35784	Biso	1.000	O
Ce19	1.0000	2.39816	5.80030	14.69690	Biso	1.000	Ce
O34	1.0000	1.59549	7.75072	13.57064	Biso	1.000	O
O35	1.0000	6.32020	7.75301	13.57662	Biso	1.000	O
O36	1.0000	4.82745	5.80819	14.81179	Biso	1.000	O
Ce20	1.0000	5.56775	7.75254	15.86079	Biso	1.000	Ce
Ce21	1.0000	2.33943	7.74422	18.00152	Biso	1.000	Ce
O37	1.0000	1.58982	5.76142	16.93544	Biso	1.000	O
O38	1.0000	6.44935	5.82194	17.19459	Biso	1.000	O
O39	1.0000	7.88309	7.75600	15.77542	Biso	1.000	O
O40	1.0000	3.12427	7.73131	15.78120	Biso	1.000	O
Ce22	1.0000	5.37386	5.83976	19.23631	Biso	1.000	Ce
O41	1.0000	4.61480	7.75609	17.92751	Biso	1.000	O
O42	1.0000	0.02778	7.77146	17.93623	Biso	1.000	O
O43	1.0000	3.09814	5.81919	19.22086	Biso	1.000	O
O44	1.0000	5.90039	7.73417	20.18522	Biso	1.000	O
Ce23	1.0000	-0.77087	9.69146	10.23856	Biso	1.000	Ce
Ce24	1.0000	-0.71313	9.68978	16.87489	Biso	1.000	Ce
O45	1.0000	0.02059	9.69146	8.00000	Biso	1.000	O
O46	1.0000	1.60349	9.69146	10.23856	Biso	1.000	O
O47	1.0000	0.01963	9.69198	14.70614	Biso	1.000	O
Ce25	1.0000	5.56074	11.63011	9.11928	Biso	1.000	Ce
Ce26	1.0000	2.39494	9.69146	8.00000	Biso	1.000	Ce
O48	1.0000	4.76929	9.69146	8.00000	Biso	1.000	O
O49	1.0000	7.93510	11.63011	9.11928	Biso	1.000	O
O50	1.0000	3.18639	11.63011	9.11928	Biso	1.000	O
O51	1.0000	6.35219	9.69146	10.23856	Biso	1.000	O
Ce27	1.0000	2.39494	11.63011	11.35784	Biso	1.000	Ce
Ce28	1.0000	-0.78063	11.62923	13.53554	Biso	1.000	Ce
Ce29	1.0000	5.56074	9.69146	12.47712	Biso	1.000	Ce
O52	1.0000	7.93510	9.69146	12.47712	Biso	1.000	O
O53	1.0000	3.18639	9.69146	12.47712	Biso	1.000	O
O54	1.0000	4.76929	11.63011	11.35784	Biso	1.000	O
O55	1.0000	0.02059	11.63011	11.35784	Biso	1.000	O
Ce30	1.0000	2.38945	9.69277	14.67934	Biso	1.000	Ce

O56 1.0000 1.59545 11.63281 13.56940 Biso 1.000 O
O57 1.0000 6.32020 11.63032 13.57662 Biso 1.000 O
O58 1.0000 4.81597 9.69369 14.81776 Biso 1.000 O
Ce31 1.0000 5.56918 11.62783 15.86152 Biso 1.000 Ce
Ce32 1.0000 2.32898 11.63461 18.00657 Biso 1.000 Ce
O59 1.0000 1.62701 9.69389 16.88546 Biso 1.000 O
O60 1.0000 6.48777 9.69467 17.24080 Biso 1.000 O
O61 1.0000 7.89197 11.63345 15.77879 Biso 1.000 O
O62 1.0000 3.11803 11.65796 15.78428 Biso 1.000 O
Ce33 1.0000 5.29041 9.68363 19.25677 Biso 1.000 Ce
O63 1.0000 4.60855 11.62454 17.92429 Biso 1.000 O
O64 1.0000 0.02327 11.60857 17.96742 Biso 1.000 O
O65 1.0000 3.04538 9.68673 19.18844 Biso 1.000 O
O66 1.0000 5.84393 11.62460 20.20772 Biso 1.000 O
Rh1 1.0000 8.62441 3.88667 20.18743 Biso 1.000 Rh
Si1 1.0000 9.14379 5.53859 21.75068 Biso 1.000 Si
C1 1.0000 10.80576 5.43822 22.65863 Biso 1.000 C
H1 1.0000 10.87094 6.26964 23.38035 Biso 1.000 H
H2 1.0000 11.61191 5.57269 21.91921 Biso 1.000 H
H3 1.0000 10.98875 4.50225 23.19928 Biso 1.000 H
C2 1.0000 9.22528 7.27257 20.99471 Biso 1.000 C
H4 1.0000 8.40318 7.52260 20.31427 Biso 1.000 H
H5 1.0000 10.18215 7.43116 20.47916 Biso 1.000 H
H6 1.0000 9.18767 7.98058 21.84119 Biso 1.000 H
C3 1.0000 7.69083 5.61801 22.97878 Biso 1.000 C
H7 1.0000 6.84012 6.11389 22.48523 Biso 1.000 H
H8 1.0000 7.97716 6.23301 23.84888 Biso 1.000 H
H9 1.0000 7.35535 4.63740 23.34022 Biso 1.000 H
H10 1.0000 8.29431 1.16533 19.83387 Biso 1.000 H
C4 1.0000 8.48118 2.41915 21.65637 Biso 1.000 C
C5 1.0000 8.78378 1.18730 20.82230 Biso 1.000 C
H11 1.0000 7.40484 2.48854 21.89643 Biso 1.000 H
H12 1.0000 8.41337 0.27110 21.31866 Biso 1.000 H
H13 1.0000 9.86689 1.08503 20.65452 Biso 1.000 H
C6 1.0000 9.30078 2.39853 22.93190 Biso 1.000 C
H14 1.0000 9.05798 1.48397 23.50489 Biso 1.000 H
H15 1.0000 9.10228 3.25420 23.58927 Biso 1.000 H
H16 1.0000 10.37905 2.36935 22.71328 Biso 1.000 H
H17 1.0000 10.16620 3.88133 20.55317 Biso 1.000 H

RhHSiMe3propyl_1x3-CeO2-211
data_image0
loop_
_atom_site_label
_atom_site_occupancy
_atom_site_Cartn_x
_atom_site_Cartn_y
_atom_site_Cartn_z
_atom_site_thermal_displace_type
_atom_site_B_iso_or_equiv
_atom_site_type_symbol
Ce1 1.0000 -0.77087 1.93685 10.23856 Biso 1.000 Ce
Ce2 1.0000 -0.73731 1.89212 16.90810 Biso 1.000 Ce
O1 1.0000 0.02059 1.93685 8.00000 Biso 1.000 O
O2 1.0000 1.60349 1.93685 10.23856 Biso 1.000 O
O3 1.0000 0.01270 1.93206 14.72074 Biso 1.000 O
Ce3 1.0000 5.56074 3.87550 9.11928 Biso 1.000 Ce
Ce4 1.0000 2.39494 1.93685 8.00000 Biso 1.000 Ce
O4 1.0000 4.76929 1.93685 8.00000 Biso 1.000 O

O5	1.0000	7.93510	3.87550	9.11928	Biso	1.000	O
O6	1.0000	3.18639	3.87550	9.11928	Biso	1.000	O
O7	1.0000	6.35219	1.93685	10.23856	Biso	1.000	O
Ce5	1.0000	2.39494	3.87550	11.35784	Biso	1.000	Ce
Ce6	1.0000	-0.78708	3.87854	13.57076	Biso	1.000	Ce
Ce7	1.0000	5.56074	1.93685	12.47712	Biso	1.000	Ce
O8	1.0000	7.93510	1.93685	12.47712	Biso	1.000	O
O9	1.0000	3.18639	1.93685	12.47712	Biso	1.000	O
O10	1.0000	4.76929	3.87550	11.35784	Biso	1.000	O
O11	1.0000	0.02059	3.87550	11.35784	Biso	1.000	O
Ce8	1.0000	2.39213	1.94105	14.69799	Biso	1.000	Ce
O12	1.0000	1.59383	3.87143	13.57627	Biso	1.000	O
O13	1.0000	6.32020	3.87571	13.57662	Biso	1.000	O
O14	1.0000	4.82513	1.93722	14.80198	Biso	1.000	O
Ce9	1.0000	5.56767	3.88439	15.88365	Biso	1.000	Ce
Ce10	1.0000	2.38268	3.88456	18.04332	Biso	1.000	Ce
O15	1.0000	1.57026	1.97042	16.91902	Biso	1.000	O
O16	1.0000	6.45140	1.91191	17.20590	Biso	1.000	O
O17	1.0000	7.92030	3.86027	15.85031	Biso	1.000	O
O18	1.0000	3.15925	3.87227	15.80639	Biso	1.000	O
Ce11	1.0000	5.31456	1.85822	19.26511	Biso	1.000	Ce
O19	1.0000	4.64944	3.86052	17.99998	Biso	1.000	O
O20	1.0000	0.01560	3.90838	18.18949	Biso	1.000	O
O21	1.0000	3.06393	1.92269	19.20334	Biso	1.000	O
O22	1.0000	6.07438	3.89149	20.24731	Biso	1.000	O
Ce12	1.0000	-0.77087	5.81416	10.23856	Biso	1.000	Ce
Ce13	1.0000	-0.76153	5.84638	16.94200	Biso	1.000	Ce
O23	1.0000	0.02059	5.81416	8.00000	Biso	1.000	O
O24	1.0000	1.60349	5.81416	10.23856	Biso	1.000	O
O25	1.0000	0.00136	5.81257	14.71678	Biso	1.000	O
Ce14	1.0000	5.56074	7.75281	9.11928	Biso	1.000	Ce
Ce15	1.0000	2.39494	5.81416	8.00000	Biso	1.000	Ce
O26	1.0000	4.76929	5.81416	8.00000	Biso	1.000	O
O27	1.0000	7.93510	7.75281	9.11928	Biso	1.000	O
O28	1.0000	3.18639	7.75281	9.11928	Biso	1.000	O
O29	1.0000	6.35219	5.81416	10.23856	Biso	1.000	O
Ce16	1.0000	2.39494	7.75281	11.35784	Biso	1.000	Ce
Ce17	1.0000	-0.78285	7.74680	13.55444	Biso	1.000	Ce
Ce18	1.0000	5.56074	5.81416	12.47712	Biso	1.000	Ce
O30	1.0000	7.93510	5.81416	12.47712	Biso	1.000	O
O31	1.0000	3.18639	5.81416	12.47712	Biso	1.000	O
O32	1.0000	4.76929	7.75281	11.35784	Biso	1.000	O
O33	1.0000	0.02059	7.75281	11.35784	Biso	1.000	O
Ce19	1.0000	2.38391	5.80728	14.69315	Biso	1.000	Ce
O34	1.0000	1.59148	7.75009	13.56110	Biso	1.000	O
O35	1.0000	6.32020	7.75301	13.57662	Biso	1.000	O
O36	1.0000	4.79914	5.80569	14.76521	Biso	1.000	O
Ce20	1.0000	5.56090	7.74656	15.84938	Biso	1.000	Ce
Ce21	1.0000	2.33090	7.74864	17.99246	Biso	1.000	Ce
O37	1.0000	1.57718	5.78531	16.90617	Biso	1.000	O
O38	1.0000	6.36721	5.85312	17.07063	Biso	1.000	O
O39	1.0000	7.89632	7.76092	15.78884	Biso	1.000	O
O40	1.0000	3.13180	7.73428	15.76848	Biso	1.000	O
Ce22	1.0000	5.36059	5.89669	19.22241	Biso	1.000	Ce
O41	1.0000	4.61111	7.78532	17.92808	Biso	1.000	O
O42	1.0000	0.01873	7.77636	17.99207	Biso	1.000	O
O43	1.0000	3.07944	5.83693	19.17539	Biso	1.000	O
O44	1.0000	5.83569	7.78232	20.23966	Biso	1.000	O
Ce23	1.0000	-0.77087	9.69146	10.23856	Biso	1.000	Ce
Ce24	1.0000	-0.72339	9.67816	16.87953	Biso	1.000	Ce

O45 1.0000 0.02059 9.69146 8.00000 Biso 1.000 O
O46 1.0000 1.60349 9.69146 10.23856 Biso 1.000 O
O47 1.0000 0.01613 9.69458 14.69995 Biso 1.000 O
Ce25 1.0000 5.56074 11.63011 9.11928 Biso 1.000 Ce
Ce26 1.0000 2.39494 9.69146 8.00000 Biso 1.000 Ce
O48 1.0000 4.76929 9.69146 8.00000 Biso 1.000 O
O49 1.0000 7.93510 11.63011 9.11928 Biso 1.000 O
O50 1.0000 3.18639 11.63011 9.11928 Biso 1.000 O
O51 1.0000 6.35219 9.69146 10.23856 Biso 1.000 O
Ce27 1.0000 2.39494 11.63011 11.35784 Biso 1.000 Ce
Ce28 1.0000 -0.78422 11.63175 13.54904 Biso 1.000 Ce
Ce29 1.0000 5.56074 9.69146 12.47712 Biso 1.000 Ce
O52 1.0000 7.93510 9.69146 12.47712 Biso 1.000 O
O53 1.0000 3.18639 9.69146 12.47712 Biso 1.000 O
O54 1.0000 4.76929 11.63011 11.35784 Biso 1.000 O
O55 1.0000 0.02059 11.63011 11.35784 Biso 1.000 O
Ce30 1.0000 2.39139 9.69107 14.68006 Biso 1.000 Ce
O56 1.0000 1.59514 11.63469 13.56070 Biso 1.000 O
O57 1.0000 6.32020 11.63032 13.57662 Biso 1.000 O
O58 1.0000 4.81683 9.69974 14.80104 Biso 1.000 O
Ce31 1.0000 5.57112 11.63103 15.86889 Biso 1.000 Ce
Ce32 1.0000 2.32693 11.61916 18.00172 Biso 1.000 Ce
O59 1.0000 1.62282 9.67701 16.86782 Biso 1.000 O
O60 1.0000 6.48713 9.70638 17.23842 Biso 1.000 O
O61 1.0000 7.89405 11.63924 15.78558 Biso 1.000 O
O62 1.0000 3.12277 11.64638 15.77851 Biso 1.000 O
Ce33 1.0000 5.26645 9.68355 19.26975 Biso 1.000 Ce
O63 1.0000 4.60634 11.62428 17.92995 Biso 1.000 O
O64 1.0000 0.03586 11.56758 17.95799 Biso 1.000 O
O65 1.0000 3.01180 9.69211 19.18384 Biso 1.000 O
O66 1.0000 5.74994 11.59996 20.26597 Biso 1.000 O
Rh1 1.0000 7.93254 4.00023 19.50106 Biso 1.000 Rh
C1 1.0000 8.41228 1.97521 20.02816 Biso 1.000 C
C2 1.0000 8.30589 1.50109 21.47797 Biso 1.000 C
H1 1.0000 9.47797 1.91420 19.73491 Biso 1.000 H
H2 1.0000 7.92502 1.17545 19.41900 Biso 1.000 H
H3 1.0000 8.85782 2.18978 22.13511 Biso 1.000 H
H4 1.0000 7.25809 1.52086 21.81930 Biso 1.000 H
H5 1.0000 7.77665 5.65563 19.19216 Biso 1.000 H
Si1 1.0000 8.92031 5.05341 21.36077 Biso 1.000 Si
C3 1.0000 10.64007 4.29026 21.58965 Biso 1.000 C
H6 1.0000 10.93935 4.39012 22.64691 Biso 1.000 H
H7 1.0000 11.36845 4.86398 20.99280 Biso 1.000 H
H8 1.0000 10.71061 3.22745 21.32177 Biso 1.000 H
C4 1.0000 9.14364 6.91535 21.22993 Biso 1.000 C
H9 1.0000 8.16753 7.42358 21.20451 Biso 1.000 H
H10 1.0000 9.68095 7.20642 20.31811 Biso 1.000 H
H11 1.0000 9.71288 7.27067 22.10574 Biso 1.000 H
C5 1.0000 7.78927 4.76631 22.84337 Biso 1.000 C
H12 1.0000 6.88105 5.37521 22.72633 Biso 1.000 H
H13 1.0000 8.31466 5.10488 23.75281 Biso 1.000 H
H14 1.0000 7.48459 3.72210 22.97576 Biso 1.000 H
C6 1.0000 8.86108 0.08414 21.65373 Biso 1.000 C
H15 1.0000 8.79725 -0.24722 22.70171 Biso 1.000 H
H16 1.0000 9.91833 0.03279 21.34589 Biso 1.000 H
H17 1.0000 8.29377 -0.62992 21.03860 Biso 1.000 H

TS_RhH2SiMe3propen_RhHSiMe3propyl_1x3-CeO2-211
data_image0

```
loop_
  _atom_site_label
  _atom_site_occupancy
  _atom_site_Cartn_x
  _atom_site_Cartn_y
  _atom_site_Cartn_z
  _atom_site_thermal_displace_type
  _atom_site_B_iso_or_equiv
  _atom_site_type_symbol
  Ce1      1.0000 -0.77087  1.93685  10.23856  Biso   1.000  Ce
  Ce2      1.0000 -0.73323  1.93842  16.89274  Biso   1.000  Ce
  O1       1.0000 0.02059  1.93685  8.00000  Biso   1.000  O
  O2       1.0000 1.60349  1.93685  10.23856  Biso   1.000  O
  O3       1.0000 0.01682  1.94192  14.72140  Biso   1.000  O
  Ce3      1.0000 5.56074  3.87550  9.11928  Biso   1.000  Ce
  Ce4      1.0000 2.39494  1.93685  8.00000  Biso   1.000  Ce
  O4       1.0000 4.76929  1.93685  8.00000  Biso   1.000  O
  O5       1.0000 7.93510  3.87550  9.11928  Biso   1.000  O
  O6       1.0000 3.18639  3.87550  9.11928  Biso   1.000  O
  O7       1.0000 6.35219  1.93685  10.23856  Biso   1.000  O
  Ce5      1.0000 2.39494  3.87550  11.35784  Biso   1.000  Ce
  Ce6      1.0000 -0.78795  3.87705  13.54320  Biso   1.000  Ce
  Ce7      1.0000 5.56074  1.93685  12.47712  Biso   1.000  Ce
  O8       1.0000 7.93510  1.93685  12.47712  Biso   1.000  O
  O9       1.0000 3.18639  1.93685  12.47712  Biso   1.000  O
  O10      1.0000 4.76929  3.87550  11.35784  Biso   1.000  O
  O11      1.0000 0.02059  3.87550  11.35784  Biso   1.000  O
  Ce8      1.0000 2.39206  1.94362  14.68553  Biso   1.000  Ce
  O12      1.0000 1.59473  3.87649  13.58293  Biso   1.000  O
  O13      1.0000 6.32020  3.87571  13.57662  Biso   1.000  O
  O14      1.0000 4.82449  1.94170  14.81310  Biso   1.000  O
  Ce9      1.0000 5.56377  3.87779  15.87932  Biso   1.000  Ce
  Ce10     1.0000 2.35209  3.87900  18.04110  Biso   1.000  Ce
  O15      1.0000 1.58921  1.97786  16.92695  Biso   1.000  O
  O16      1.0000 6.44947  1.91584  17.19031  Biso   1.000  O
  O17      1.0000 7.90119  3.87987  15.81775  Biso   1.000  O
  O18      1.0000 3.15461  3.87662  15.80430  Biso   1.000  O
  Ce11     1.0000 5.35536  1.88577  19.21468  Biso   1.000  Ce
  O19      1.0000 4.61421  3.88058  17.99595  Biso   1.000  O
  O20      1.0000 -0.10344  3.85183  18.18712  Biso   1.000  O
  O21      1.0000 3.09361  1.94483  19.21617  Biso   1.000  O
  O22      1.0000 6.42583  3.96423  19.89912  Biso   1.000  O
  Ce12     1.0000 -0.77087  5.81416  10.23856  Biso   1.000  Ce
  Ce13     1.0000 -0.73704  5.81343  16.90475  Biso   1.000  Ce
  O23      1.0000 0.02059  5.81416  8.00000  Biso   1.000  O
  O24      1.0000 1.60349  5.81416  10.23856  Biso   1.000  O
  O25      1.0000 0.01228  5.81249  14.72703  Biso   1.000  O
  Ce14     1.0000 5.56074  7.75281  9.11928  Biso   1.000  Ce
  Ce15     1.0000 2.39494  5.81416  8.00000  Biso   1.000  Ce
  O26      1.0000 4.76929  5.81416  8.00000  Biso   1.000  O
  O27      1.0000 7.93510  7.75281  9.11928  Biso   1.000  O
  O28      1.0000 3.18639  7.75281  9.11928  Biso   1.000  O
  O29      1.0000 6.35219  5.81416  10.23856  Biso   1.000  O
  Ce16     1.0000 2.39494  7.75281  11.35784  Biso   1.000  Ce
  Ce17     1.0000 -0.78315  7.75080  13.53916  Biso   1.000  Ce
  Ce18     1.0000 5.56074  5.81416  12.47712  Biso   1.000  Ce
  O30      1.0000 7.93510  5.81416  12.47712  Biso   1.000  O
  O31      1.0000 3.18639  5.81416  12.47712  Biso   1.000  O
  O32      1.0000 4.76929  7.75281  11.35784  Biso   1.000  O
  O33      1.0000 0.02059  7.75281  11.35784  Biso   1.000  O
```

Ce19	1.0000	2.38873	5.80689	14.68528	Biso	1.000	Ce
O34	1.0000	1.59130	7.74802	13.57194	Biso	1.000	O
O35	1.0000	6.32020	7.75301	13.57662	Biso	1.000	O
O36	1.0000	4.82444	5.81111	14.81261	Biso	1.000	O
Ce20	1.0000	5.57590	7.75473	15.85393	Biso	1.000	Ce
Ce21	1.0000	2.32696	7.75017	17.99329	Biso	1.000	Ce
O37	1.0000	1.57502	5.76219	16.92569	Biso	1.000	O
O38	1.0000	6.45291	5.84743	17.18926	Biso	1.000	O
O39	1.0000	7.89509	7.75591	15.79004	Biso	1.000	O
O40	1.0000	3.11846	7.73188	15.78074	Biso	1.000	O
Ce22	1.0000	5.34116	5.89198	19.21465	Biso	1.000	Ce
O41	1.0000	4.60600	7.76724	17.92300	Biso	1.000	O
O42	1.0000	0.02326	7.75759	17.98571	Biso	1.000	O
O43	1.0000	3.06986	5.82075	19.20147	Biso	1.000	O
O44	1.0000	5.81327	7.77113	20.23036	Biso	1.000	O
Ce23	1.0000	-0.77087	9.69146	10.23856	Biso	1.000	Ce
Ce24	1.0000	-0.72202	9.69069	16.87487	Biso	1.000	Ce
O45	1.0000	0.02059	9.69146	8.00000	Biso	1.000	O
O46	1.0000	1.60349	9.69146	10.23856	Biso	1.000	O
O47	1.0000	0.01683	9.69112	14.70562	Biso	1.000	O
Ce25	1.0000	5.56074	11.63011	9.11928	Biso	1.000	Ce
Ce26	1.0000	2.39494	9.69146	8.00000	Biso	1.000	Ce
O48	1.0000	4.76929	9.69146	8.00000	Biso	1.000	O
O49	1.0000	7.93510	11.63011	9.11928	Biso	1.000	O
O50	1.0000	3.18639	11.63011	9.11928	Biso	1.000	O
O51	1.0000	6.35219	9.69146	10.23856	Biso	1.000	O
Ce27	1.0000	2.39494	11.63011	11.35784	Biso	1.000	Ce
Ce28	1.0000	-0.78196	11.63193	13.53753	Biso	1.000	Ce
Ce29	1.0000	5.56074	9.69146	12.47712	Biso	1.000	Ce
O52	1.0000	7.93510	9.69146	12.47712	Biso	1.000	O
O53	1.0000	3.18639	9.69146	12.47712	Biso	1.000	O
O54	1.0000	4.76929	11.63011	11.35784	Biso	1.000	O
O55	1.0000	0.02059	11.63011	11.35784	Biso	1.000	O
Ce30	1.0000	2.38801	9.68891	14.67250	Biso	1.000	Ce
O56	1.0000	1.59184	11.63388	13.57281	Biso	1.000	O
O57	1.0000	6.32020	11.63032	13.57662	Biso	1.000	O
O58	1.0000	4.81986	9.69062	14.81839	Biso	1.000	O
Ce31	1.0000	5.57767	11.63057	15.85468	Biso	1.000	Ce
Ce32	1.0000	2.33352	11.62441	17.98278	Biso	1.000	Ce
O59	1.0000	1.61050	9.68800	16.87812	Biso	1.000	O
O60	1.0000	6.48991	9.69689	17.24511	Biso	1.000	O
O61	1.0000	7.89849	11.62811	15.78962	Biso	1.000	O
O62	1.0000	3.12374	11.64323	15.77663	Biso	1.000	O
Ce33	1.0000	5.27317	9.70428	19.26235	Biso	1.000	Ce
O63	1.0000	4.61201	11.63539	17.92359	Biso	1.000	O
O64	1.0000	0.03024	11.60819	18.00010	Biso	1.000	O
O65	1.0000	3.02195	9.69873	19.18070	Biso	1.000	O
O66	1.0000	5.81218	11.61135	20.23315	Biso	1.000	O
Rh1	1.0000	8.39561	3.58964	20.36976	Biso	1.000	Rh
Si1	1.0000	9.15583	5.36370	21.88842	Biso	1.000	Si
C1	1.0000	11.00362	5.32645	22.31419	Biso	1.000	C
H1	1.0000	11.22082	6.21125	22.93778	Biso	1.000	H
H2	1.0000	11.62477	5.40051	21.40757	Biso	1.000	H
H3	1.0000	11.30578	4.43877	22.88589	Biso	1.000	H
C2	1.0000	8.85403	7.06587	21.14450	Biso	1.000	C
H4	1.0000	7.78607	7.31924	21.06531	Biso	1.000	H
H5	1.0000	9.29590	7.19389	20.14717	Biso	1.000	H
H6	1.0000	9.33126	7.79583	21.82401	Biso	1.000	H
C3	1.0000	8.21844	5.30039	23.53562	Biso	1.000	C
H7	1.0000	7.13392	5.39886	23.37821	Biso	1.000	H

H8 1.0000 8.54782 6.13811 24.17361 Biso 1.000 H
H9 1.0000 8.40022 4.36429 24.08507 Biso 1.000 H
H10 1.0000 9.94807 3.48235 20.81932 Biso 1.000 H
C4 1.0000 8.50477 1.41558 20.64770 Biso 1.000 C
C5 1.0000 9.80117 1.93738 20.96023 Biso 1.000 C
H11 1.0000 7.84870 1.07915 21.45267 Biso 1.000 H
H12 1.0000 10.57814 1.77696 20.20145 Biso 1.000 H
H13 1.0000 7.82123 3.51503 21.78777 Biso 1.000 H
C6 1.0000 10.34465 1.80196 22.36774 Biso 1.000 C
H14 1.0000 10.57454 0.73881 22.54180 Biso 1.000 H
H15 1.0000 9.61103 2.12096 23.12112 Biso 1.000 H
H16 1.0000 11.27340 2.36929 22.50972 Biso 1.000 H
H17 1.0000 8.40945 0.83625 19.72508 Biso 1.000 H

TS_RhHpropen_Rhpropyl_1x3-CeO2-211
data_image0
loop_
_atom_site_label
_atom_site_occupancy
_atom_site_Cartn_x
_atom_site_Cartn_y
_atom_site_Cartn_z
_atom_site_thermal_displace_type
_atom_site_B_iso_or_equiv
_atom_site_type_symbol
Ce1 1.0000 -0.77087 1.93685 10.23856 Biso 1.000 Ce
Ce2 1.0000 -0.74166 1.89686 16.88727 Biso 1.000 Ce
O1 1.0000 0.02059 1.93685 8.00000 Biso 1.000 O
O2 1.0000 1.60349 1.93685 10.23856 Biso 1.000 O
O3 1.0000 -0.00704 1.94392 14.72166 Biso 1.000 O
Ce3 1.0000 5.56074 3.87550 9.11928 Biso 1.000 Ce
Ce4 1.0000 2.39494 1.93685 8.00000 Biso 1.000 Ce
O4 1.0000 4.76929 1.93685 8.00000 Biso 1.000 O
O5 1.0000 7.93510 3.87550 9.11928 Biso 1.000 O
O6 1.0000 3.18639 3.87550 9.11928 Biso 1.000 O
O7 1.0000 6.35219 1.93685 10.23856 Biso 1.000 O
Ce5 1.0000 2.39494 3.87550 11.35784 Biso 1.000 Ce
Ce6 1.0000 -0.78747 3.88095 13.54847 Biso 1.000 Ce
Ce7 1.0000 5.56074 1.93685 12.47712 Biso 1.000 Ce
O8 1.0000 7.93510 1.93685 12.47712 Biso 1.000 O
O9 1.0000 3.18639 1.93685 12.47712 Biso 1.000 O
O10 1.0000 4.76929 3.87550 11.35784 Biso 1.000 O
O11 1.0000 0.02059 3.87550 11.35784 Biso 1.000 O
Ce8 1.0000 2.37291 1.93326 14.67985 Biso 1.000 Ce
O12 1.0000 1.59639 3.87376 13.58739 Biso 1.000 O
O13 1.0000 6.32020 3.87571 13.57662 Biso 1.000 O
O14 1.0000 4.81884 1.92705 14.80268 Biso 1.000 O
Ce9 1.0000 5.55171 3.79014 15.86278 Biso 1.000 Ce
Ce10 1.0000 2.40917 3.87047 18.02318 Biso 1.000 Ce
O15 1.0000 1.58344 1.95763 16.91739 Biso 1.000 O
O16 1.0000 6.40915 1.89639 17.19012 Biso 1.000 O
O17 1.0000 7.87728 3.93239 15.84453 Biso 1.000 O
O18 1.0000 3.16018 3.84892 15.78870 Biso 1.000 O
Ce11 1.0000 5.29372 1.84644 19.26496 Biso 1.000 Ce
O19 1.0000 4.65115 3.95637 18.00177 Biso 1.000 O
O20 1.0000 0.02471 3.89377 18.17017 Biso 1.000 O
O21 1.0000 3.05081 1.94572 19.22191 Biso 1.000 O
O22 1.0000 6.02956 3.84111 20.34684 Biso 1.000 O
Ce12 1.0000 -0.77087 5.81416 10.23856 Biso 1.000 Ce

Ce13 1.0000 -0.63638 5.85288 16.91728 Biso 1.000 Ce
O23 1.0000 0.02059 5.81416 8.00000 Biso 1.000 O
O24 1.0000 1.60349 5.81416 10.23856 Biso 1.000 O
O25 1.0000 0.05523 5.82410 14.70171 Biso 1.000 O
Ce14 1.0000 5.56074 7.75281 9.11928 Biso 1.000 Ce
Ce15 1.0000 2.39494 5.81416 8.00000 Biso 1.000 Ce
O26 1.0000 4.76929 5.81416 8.00000 Biso 1.000 O
O27 1.0000 7.93510 7.75281 9.11928 Biso 1.000 O
O28 1.0000 3.18639 7.75281 9.11928 Biso 1.000 O
O29 1.0000 6.35219 5.81416 10.23856 Biso 1.000 O
Ce16 1.0000 2.39494 7.75281 11.35784 Biso 1.000 Ce
Ce17 1.0000 -0.78179 7.73797 13.53617 Biso 1.000 Ce
Ce18 1.0000 5.56074 5.81416 12.47712 Biso 1.000 Ce
O30 1.0000 7.93510 5.81416 12.47712 Biso 1.000 O
O31 1.0000 3.18639 5.81416 12.47712 Biso 1.000 O
O32 1.0000 4.76929 7.75281 11.35784 Biso 1.000 O
O33 1.0000 0.02059 7.75281 11.35784 Biso 1.000 O
Ce19 1.0000 2.40986 5.80856 14.68056 Biso 1.000 Ce
O34 1.0000 1.59786 7.75457 13.57603 Biso 1.000 O
O35 1.0000 6.32020 7.75301 13.57662 Biso 1.000 O
O36 1.0000 4.88912 5.81532 14.92425 Biso 1.000 O
Ce20 1.0000 5.52274 7.84716 15.81884 Biso 1.000 Ce
Ce21 1.0000 2.36661 7.74293 18.00685 Biso 1.000 Ce
O37 1.0000 1.65733 5.78834 16.90777 Biso 1.000 O
O38 1.0000 7.04007 5.48984 18.21541 Biso 1.000 O
O39 1.0000 7.82084 7.64952 15.87591 Biso 1.000 O
O40 1.0000 3.13549 7.78042 15.78499 Biso 1.000 O
Ce22 1.0000 5.21920 5.88730 19.50497 Biso 1.000 Ce
O41 1.0000 4.70780 7.55269 17.93318 Biso 1.000 O
O42 1.0000 0.08043 7.77601 18.02239 Biso 1.000 O
O43 1.0000 2.97297 5.80488 19.29932 Biso 1.000 O
O44 1.0000 5.69280 7.83775 20.39601 Biso 1.000 O
Ce23 1.0000 -0.77087 9.69146 10.23856 Biso 1.000 Ce
Ce24 1.0000 -0.72735 9.68138 16.91048 Biso 1.000 Ce
O45 1.0000 0.02059 9.69146 8.00000 Biso 1.000 O
O46 1.0000 1.60349 9.69146 10.23856 Biso 1.000 O
O47 1.0000 -0.02531 9.64458 14.73576 Biso 1.000 O
Ce25 1.0000 5.56074 11.63011 9.11928 Biso 1.000 Ce
Ce26 1.0000 2.39494 9.69146 8.00000 Biso 1.000 Ce
O48 1.0000 4.76929 9.69146 8.00000 Biso 1.000 O
O49 1.0000 7.93510 11.63011 9.11928 Biso 1.000 O
O50 1.0000 3.18639 11.63011 9.11928 Biso 1.000 O
O51 1.0000 6.35219 9.69146 10.23856 Biso 1.000 O
Ce27 1.0000 2.39494 11.63011 11.35784 Biso 1.000 Ce
Ce28 1.0000 -0.80214 11.62643 13.53117 Biso 1.000 Ce
Ce29 1.0000 5.56074 9.69146 12.47712 Biso 1.000 Ce
O52 1.0000 7.93510 9.69146 12.47712 Biso 1.000 O
O53 1.0000 3.18639 9.69146 12.47712 Biso 1.000 O
O54 1.0000 4.76929 11.63011 11.35784 Biso 1.000 O
O55 1.0000 0.02059 11.63011 11.35784 Biso 1.000 O
Ce30 1.0000 2.36078 9.70148 14.67149 Biso 1.000 Ce
O56 1.0000 1.56690 11.62241 13.58337 Biso 1.000 O
O57 1.0000 6.32020 11.63032 13.57662 Biso 1.000 O
O58 1.0000 4.80581 9.71647 14.80059 Biso 1.000 O
Ce31 1.0000 5.56998 11.63457 15.88124 Biso 1.000 Ce
Ce32 1.0000 2.30743 11.62354 17.98702 Biso 1.000 Ce
O59 1.0000 1.59607 9.70529 16.88760 Biso 1.000 O
O60 1.0000 6.45668 9.69187 17.24565 Biso 1.000 O
O61 1.0000 7.90067 11.61029 15.80412 Biso 1.000 O
O62 1.0000 3.11669 11.64378 15.78482 Biso 1.000 O

Ce33 1.0000 5.29620 9.69243 19.29740 Biso 1.000 Ce
O63 1.0000 4.57981 11.61364 17.96404 Biso 1.000 O
O64 1.0000 0.00509 11.60973 18.04888 Biso 1.000 O
O65 1.0000 3.06420 9.63376 19.16249 Biso 1.000 O
O66 1.0000 5.87323 11.60044 20.24746 Biso 1.000 O
Rh1 1.0000 7.80533 3.91819 19.32527 Biso 1.000 Rh
H1 1.0000 8.12201 2.39284 19.71086 Biso 1.000 H
C1 1.0000 8.94825 4.41198 21.00721 Biso 1.000 C
C2 1.0000 8.92802 2.97526 20.97299 Biso 1.000 C
H2 1.0000 8.31789 4.92807 21.73748 Biso 1.000 H
H3 1.0000 9.83747 4.95896 20.67975 Biso 1.000 H
H4 1.0000 9.84121 2.49634 20.59281 Biso 1.000 H
C3 1.0000 8.19466 2.18534 22.03256 Biso 1.000 C
H5 1.0000 8.84269 2.09791 22.91925 Biso 1.000 H
H6 1.0000 7.93505 1.17174 21.69323 Biso 1.000 H
H7 1.0000 7.26878 2.69970 22.32346 Biso 1.000 H

TS_RhHSiMe3propyl_RhH_1x3-CeO2-211
data_image0
loop_
_atom_site_label
_atom_site_occupancy
_atom_site_Cartn_x
_atom_site_Cartn_y
_atom_site_Cartn_z
_atom_site_thermal_displace_type
_atom_site_B_iso_or_equiv
_atom_site_type_symbol
Ce1 1.0000 -0.77087 1.93685 10.23856 Biso 1.000 Ce
Ce2 1.0000 -0.73142 1.89580 16.88671 Biso 1.000 Ce
O1 1.0000 0.02059 1.93685 8.00000 Biso 1.000 O
O2 1.0000 1.60349 1.93685 10.23856 Biso 1.000 O
O3 1.0000 0.01115 1.93488 14.71365 Biso 1.000 O
Ce3 1.0000 5.56074 3.87550 9.11928 Biso 1.000 Ce
Ce4 1.0000 2.39494 1.93685 8.00000 Biso 1.000 Ce
O4 1.0000 4.76929 1.93685 8.00000 Biso 1.000 O
O5 1.0000 7.93510 3.87550 9.11928 Biso 1.000 O
O6 1.0000 3.18639 3.87550 9.11928 Biso 1.000 O
O7 1.0000 6.35219 1.93685 10.23856 Biso 1.000 O
Ce5 1.0000 2.39494 3.87550 11.35784 Biso 1.000 Ce
Ce6 1.0000 -0.78552 3.87974 13.56106 Biso 1.000 Ce
Ce7 1.0000 5.56074 1.93685 12.47712 Biso 1.000 Ce
O8 1.0000 7.93510 1.93685 12.47712 Biso 1.000 O
O9 1.0000 3.18639 1.93685 12.47712 Biso 1.000 O
O10 1.0000 4.76929 3.87550 11.35784 Biso 1.000 O
O11 1.0000 0.02059 3.87550 11.35784 Biso 1.000 O
Ce8 1.0000 2.39643 1.93863 14.69461 Biso 1.000 Ce
O12 1.0000 1.59463 3.87303 13.57955 Biso 1.000 O
O13 1.0000 6.32020 3.87571 13.57662 Biso 1.000 O
O14 1.0000 4.82383 1.93866 14.80457 Biso 1.000 O
Ce9 1.0000 5.56924 3.88753 15.87847 Biso 1.000 Ce
Ce10 1.0000 2.38804 3.88391 18.03391 Biso 1.000 Ce
O15 1.0000 1.58511 1.96088 16.90541 Biso 1.000 O
O16 1.0000 6.45279 1.88958 17.19790 Biso 1.000 O
O17 1.0000 7.92054 3.87167 15.85131 Biso 1.000 O
O18 1.0000 3.16533 3.87759 15.80803 Biso 1.000 O
Ce11 1.0000 5.31575 1.86404 19.25159 Biso 1.000 Ce
O19 1.0000 4.66689 3.86298 18.00378 Biso 1.000 O
O20 1.0000 0.04684 3.86455 18.20008 Biso 1.000 O

O21	1.0000	3.06656	1.92679	19.20288	Biso	1.000	O
O22	1.0000	6.12364	3.87323	20.26201	Biso	1.000	O
Ce12	1.0000	-0.77087	5.81416	10.23856	Biso	1.000	Ce
Ce13	1.0000	-0.75760	5.84484	16.92459	Biso	1.000	Ce
O23	1.0000	0.02059	5.81416	8.00000	Biso	1.000	O
O24	1.0000	1.60349	5.81416	10.23856	Biso	1.000	O
O25	1.0000	0.00273	5.81452	14.71478	Biso	1.000	O
Ce14	1.0000	5.56074	7.75281	9.11928	Biso	1.000	Ce
Ce15	1.0000	2.39494	5.81416	8.00000	Biso	1.000	Ce
O26	1.0000	4.76929	5.81416	8.00000	Biso	1.000	O
O27	1.0000	7.93510	7.75281	9.11928	Biso	1.000	O
O28	1.0000	3.18639	7.75281	9.11928	Biso	1.000	O
O29	1.0000	6.35219	5.81416	10.23856	Biso	1.000	O
Ce16	1.0000	2.39494	7.75281	11.35784	Biso	1.000	Ce
Ce17	1.0000	-0.78362	7.74971	13.55216	Biso	1.000	Ce
Ce18	1.0000	5.56074	5.81416	12.47712	Biso	1.000	Ce
O30	1.0000	7.93510	5.81416	12.47712	Biso	1.000	O
O31	1.0000	3.18639	5.81416	12.47712	Biso	1.000	O
O32	1.0000	4.76929	7.75281	11.35784	Biso	1.000	O
O33	1.0000	0.02059	7.75281	11.35784	Biso	1.000	O
Ce19	1.0000	2.38526	5.81212	14.69185	Biso	1.000	Ce
O34	1.0000	1.59194	7.75091	13.56322	Biso	1.000	O
O35	1.0000	6.32020	7.75301	13.57662	Biso	1.000	O
O36	1.0000	4.79539	5.80845	14.76509	Biso	1.000	O
Ce20	1.0000	5.55532	7.74899	15.85268	Biso	1.000	Ce
Ce21	1.0000	2.31917	7.74954	18.01185	Biso	1.000	Ce
O37	1.0000	1.57098	5.78774	16.91032	Biso	1.000	O
O38	1.0000	6.35883	5.87416	17.06015	Biso	1.000	O
O39	1.0000	7.90175	7.76438	15.79295	Biso	1.000	O
O40	1.0000	3.11984	7.74013	15.77904	Biso	1.000	O
Ce22	1.0000	5.36132	5.88183	19.22058	Biso	1.000	Ce
O41	1.0000	4.59399	7.78499	17.93641	Biso	1.000	O
O42	1.0000	0.00703	7.76855	18.01022	Biso	1.000	O
O43	1.0000	3.08321	5.81682	19.18435	Biso	1.000	O
O44	1.0000	5.88446	7.77369	20.20174	Biso	1.000	O
Ce23	1.0000	-0.77087	9.69146	10.23856	Biso	1.000	Ce
Ce24	1.0000	-0.72750	9.68209	16.88611	Biso	1.000	Ce
O45	1.0000	0.02059	9.69146	8.00000	Biso	1.000	O
O46	1.0000	1.60349	9.69146	10.23856	Biso	1.000	O
O47	1.0000	0.01845	9.69254	14.70558	Biso	1.000	O
Ce25	1.0000	5.56074	11.63011	9.11928	Biso	1.000	Ce
Ce26	1.0000	2.39494	9.69146	8.00000	Biso	1.000	Ce
O48	1.0000	4.76929	9.69146	8.00000	Biso	1.000	O
O49	1.0000	7.93510	11.63011	9.11928	Biso	1.000	O
O50	1.0000	3.18639	11.63011	9.11928	Biso	1.000	O
O51	1.0000	6.35219	9.69146	10.23856	Biso	1.000	O
Ce27	1.0000	2.39494	11.63011	11.35784	Biso	1.000	Ce
Ce28	1.0000	-0.78463	11.62885	13.54498	Biso	1.000	Ce
Ce29	1.0000	5.56074	9.69146	12.47712	Biso	1.000	Ce
O52	1.0000	7.93510	9.69146	12.47712	Biso	1.000	O
O53	1.0000	3.18639	9.69146	12.47712	Biso	1.000	O
O54	1.0000	4.76929	11.63011	11.35784	Biso	1.000	O
O55	1.0000	0.02059	11.63011	11.35784	Biso	1.000	O
Ce30	1.0000	2.38905	9.69124	14.68374	Biso	1.000	Ce
O56	1.0000	1.59469	11.63396	13.56396	Biso	1.000	O
O57	1.0000	6.32020	11.63032	13.57662	Biso	1.000	O
O58	1.0000	4.81486	9.69823	14.80303	Biso	1.000	O
Ce31	1.0000	5.56964	11.63053	15.87043	Biso	1.000	Ce
Ce32	1.0000	2.31960	11.62379	18.00826	Biso	1.000	Ce
O59	1.0000	1.60471	9.68676	16.88843	Biso	1.000	O

```
  O60      1.0000 6.48087  9.70288  17.23794  Biso   1.000  O
  O61      1.0000 7.89832  11.62688  15.78197  Biso   1.000  O
  O62      1.0000 3.12378  11.63939  15.78076  Biso   1.000  O
  Ce33     1.0000 5.27966  9.68380  19.26391  Biso   1.000  Ce
  O63      1.0000 4.59501  11.61566  17.93683  Biso   1.000  O
  O64      1.0000 0.00920  11.61004  17.98518  Biso   1.000  O
  O65      1.0000 3.01963  9.69563  19.19898  Biso   1.000  O
  O66      1.0000 5.84585  11.62883  20.22343  Biso   1.000  O
  Rh1      1.0000 7.99880  3.90490  19.54417  Biso   1.000  Rh
  C1       1.0000 8.92612  2.11041  20.54552  Biso   1.000  C
  C2       1.0000 8.58329  1.16286  21.69864  Biso   1.000  C
  H1       1.0000 9.97950  2.00409  20.24118  Biso   1.000  H
  H2       1.0000 8.34779  1.70803  19.67383  Biso   1.000  H
  H3       1.0000 9.03725  1.50485  22.64208  Biso   1.000  H
  H4       1.0000 7.49437  1.14948  21.85820  Biso   1.000  H
  H5       1.0000 7.68330  5.42277  19.02807  Biso   1.000  H
  Si1      1.0000 9.20447  4.13172  21.61157  Biso   1.000  Si
  C3       1.0000 10.92548  3.53902  22.15356  Biso   1.000  C
  H6       1.0000 11.26337  4.18352  22.98104  Biso   1.000  H
  H7       1.0000 11.65667  3.66054  21.33809  Biso   1.000  H
  H8       1.0000 10.97755  2.49423  22.48644  Biso   1.000  H
  C4       1.0000 9.57940  5.97657  21.26090  Biso   1.000  C
  H9       1.0000 8.70188  6.57702  20.98848  Biso   1.000  H
  H10      1.0000 10.34080  6.07616  20.47336  Biso   1.000  H
  H11      1.0000 10.01387  6.38683  22.19103  Biso   1.000  H
  C5       1.0000 7.97884  4.10798  23.04459  Biso   1.000  C
  H12      1.0000 7.07302  4.65633  22.74971  Biso   1.000  H
  H13      1.0000 8.43527  4.62283  23.90573  Biso   1.000  H
  H14      1.0000 7.67546  3.10343  23.36395  Biso   1.000  H
  C6       1.0000 9.06210  -0.26286  21.40833  Biso   1.000  C
  H15      1.0000 8.79772  -0.94630  22.22945  Biso   1.000  H
  H16      1.0000 10.15694  -0.29762  21.27916  Biso   1.000  H
  H17      1.0000 8.60420  -0.64268  20.48420  Biso   1.000  H


TS_RhH2SiMe3propen_RhHSiMe3isopropyl_1x3-CeO2-211
data_image0
loop_
  _atom_site_label
  _atom_site_occupancy
  _atom_site_Cartn_x
  _atom_site_Cartn_y
  _atom_site_Cartn_z
  _atom_site_thermal_displace_type
  _atom_site_B_iso_or_equiv
  _atom_site_type_symbol
  Ce1      1.0000 -0.77087  1.93685  10.23856  Biso   1.000  Ce
  Ce2      1.0000 -0.73256  1.93135  16.89752  Biso   1.000  Ce
  O1       1.0000 0.02059  1.93685  8.00000  Biso   1.000  O
  O2       1.0000 1.60349  1.93685  10.23856  Biso   1.000  O
  O3       1.0000 0.01754  1.93771  14.72492  Biso   1.000  O
  Ce3      1.0000 5.56074  3.87550  9.11928  Biso   1.000  Ce
  Ce4      1.0000 2.39494  1.93685  8.00000  Biso   1.000  Ce
  O4       1.0000 4.76929  1.93685  8.00000  Biso   1.000  O
  O5       1.0000 7.93510  3.87550  9.11928  Biso   1.000  O
  O6       1.0000 3.18639  3.87550  9.11928  Biso   1.000  O
  O7       1.0000 6.35219  1.93685  10.23856  Biso   1.000  O
  Ce5      1.0000 2.39494  3.87550  11.35784  Biso   1.000  Ce
  Ce6      1.0000 -0.78668  3.87615  13.54256  Biso   1.000  Ce
  Ce7      1.0000 5.56074  1.93685  12.47712  Biso   1.000  Ce
```

O8	1.0000	7.93510	1.93685	12.47712	Biso	1.000	O
O9	1.0000	3.18639	1.93685	12.47712	Biso	1.000	O
O10	1.0000	4.76929	3.87550	11.35784	Biso	1.000	O
O11	1.0000	0.02059	3.87550	11.35784	Biso	1.000	O
Ce8	1.0000	2.39073	1.94189	14.68341	Biso	1.000	Ce
O12	1.0000	1.59433	3.87674	13.58492	Biso	1.000	O
O13	1.0000	6.32020	3.87571	13.57662	Biso	1.000	O
O14	1.0000	4.82238	1.93994	14.80877	Biso	1.000	O
Ce9	1.0000	5.56894	3.87805	15.87075	Biso	1.000	Ce
Ce10	1.0000	2.36078	3.88170	18.02961	Biso	1.000	Ce
O15	1.0000	1.58932	1.97978	16.93050	Biso	1.000	O
O16	1.0000	6.44493	1.91367	17.17781	Biso	1.000	O
O17	1.0000	7.90306	3.87819	15.81518	Biso	1.000	O
O18	1.0000	3.15666	3.87699	15.80261	Biso	1.000	O
Ce11	1.0000	5.35827	1.87950	19.19474	Biso	1.000	Ce
O19	1.0000	4.61906	3.88168	17.99641	Biso	1.000	O
O20	1.0000	-0.12104	3.84130	18.20290	Biso	1.000	O
O21	1.0000	3.09800	1.93944	19.21126	Biso	1.000	O
O22	1.0000	6.44880	3.92267	19.89585	Biso	1.000	O
Ce12	1.0000	-0.77087	5.81416	10.23856	Biso	1.000	Ce
Ce13	1.0000	-0.73926	5.81568	16.90536	Biso	1.000	Ce
O23	1.0000	0.02059	5.81416	8.00000	Biso	1.000	O
O24	1.0000	1.60349	5.81416	10.23856	Biso	1.000	O
O25	1.0000	0.01432	5.81455	14.73052	Biso	1.000	O
Ce14	1.0000	5.56074	7.75281	9.11928	Biso	1.000	Ce
Ce15	1.0000	2.39494	5.81416	8.00000	Biso	1.000	Ce
O26	1.0000	4.76929	5.81416	8.00000	Biso	1.000	O
O27	1.0000	7.93510	7.75281	9.11928	Biso	1.000	O
O28	1.0000	3.18639	7.75281	9.11928	Biso	1.000	O
O29	1.0000	6.35219	5.81416	10.23856	Biso	1.000	O
Ce16	1.0000	2.39494	7.75281	11.35784	Biso	1.000	Ce
Ce17	1.0000	-0.78281	7.75110	13.53888	Biso	1.000	Ce
Ce18	1.0000	5.56074	5.81416	12.47712	Biso	1.000	Ce
O30	1.0000	7.93510	5.81416	12.47712	Biso	1.000	O
O31	1.0000	3.18639	5.81416	12.47712	Biso	1.000	O
O32	1.0000	4.76929	7.75281	11.35784	Biso	1.000	O
O33	1.0000	0.02059	7.75281	11.35784	Biso	1.000	O
Ce19	1.0000	2.38807	5.80907	14.68378	Biso	1.000	Ce
O34	1.0000	1.59157	7.74859	13.57367	Biso	1.000	O
O35	1.0000	6.32020	7.75301	13.57662	Biso	1.000	O
O36	1.0000	4.82263	5.81303	14.80974	Biso	1.000	O
Ce20	1.0000	5.57587	7.75344	15.85161	Biso	1.000	Ce
Ce21	1.0000	2.32642	7.75155	17.99888	Biso	1.000	Ce
O37	1.0000	1.57683	5.75989	16.93476	Biso	1.000	O
O38	1.0000	6.44645	5.84538	17.18430	Biso	1.000	O
O39	1.0000	7.89549	7.75946	15.79317	Biso	1.000	O
O40	1.0000	3.11330	7.73479	15.78578	Biso	1.000	O
Ce22	1.0000	5.34647	5.88509	19.20922	Biso	1.000	Ce
O41	1.0000	4.60562	7.76248	17.92638	Biso	1.000	O
O42	1.0000	0.02519	7.76173	18.00066	Biso	1.000	O
O43	1.0000	3.07525	5.81505	19.21199	Biso	1.000	O
O44	1.0000	5.83378	7.76231	20.22264	Biso	1.000	O
Ce23	1.0000	-0.77087	9.69146	10.23856	Biso	1.000	Ce
Ce24	1.0000	-0.72263	9.69284	16.88267	Biso	1.000	Ce
O45	1.0000	0.02059	9.69146	8.00000	Biso	1.000	O
O46	1.0000	1.60349	9.69146	10.23856	Biso	1.000	O
O47	1.0000	0.01828	9.69042	14.71282	Biso	1.000	O
Ce25	1.0000	5.56074	11.63011	9.11928	Biso	1.000	Ce
Ce26	1.0000	2.39494	9.69146	8.00000	Biso	1.000	Ce
O48	1.0000	4.76929	9.69146	8.00000	Biso	1.000	O

O49 1.0000 7.93510 11.63011 9.11928 Biso 1.000 O
O50 1.0000 3.18639 11.63011 9.11928 Biso 1.000 O
O51 1.0000 6.35219 9.69146 10.23856 Biso 1.000 O
Ce27 1.0000 2.39494 11.63011 11.35784 Biso 1.000 Ce
Ce28 1.0000 -0.78115 11.63115 13.53692 Biso 1.000 Ce
Ce29 1.0000 5.56074 9.69146 12.47712 Biso 1.000 Ce
O52 1.0000 7.93510 9.69146 12.47712 Biso 1.000 O
O53 1.0000 3.18639 9.69146 12.47712 Biso 1.000 O
O54 1.0000 4.76929 11.63011 11.35784 Biso 1.000 O
O55 1.0000 0.02059 11.63011 11.35784 Biso 1.000 O
Ce30 1.0000 2.38594 9.68966 14.67314 Biso 1.000 Ce
O56 1.0000 1.59123 11.63253 13.57397 Biso 1.000 O
O57 1.0000 6.32020 11.63032 13.57662 Biso 1.000 O
O58 1.0000 4.81744 9.68988 14.81765 Biso 1.000 O
Ce31 1.0000 5.57492 11.62961 15.84876 Biso 1.000 Ce
Ce32 1.0000 2.32800 11.62584 17.98926 Biso 1.000 Ce
O59 1.0000 1.60512 9.69531 16.89011 Biso 1.000 O
O60 1.0000 6.48616 9.69171 17.24380 Biso 1.000 O
O61 1.0000 7.90037 11.62171 15.79386 Biso 1.000 O
O62 1.0000 3.12056 11.64318 15.77893 Biso 1.000 O
Ce33 1.0000 5.28275 9.69749 19.25123 Biso 1.000 Ce
O63 1.0000 4.60806 11.62867 17.92374 Biso 1.000 O
O64 1.0000 0.01989 11.62478 18.03474 Biso 1.000 O
O65 1.0000 3.03101 9.69546 19.18953 Biso 1.000 O
O66 1.0000 5.88023 11.61333 20.19537 Biso 1.000 O
Rh1 1.0000 8.43996 3.70517 20.37570 Biso 1.000 Rh
Si1 1.0000 9.14515 5.45759 21.95371 Biso 1.000 Si
C1 1.0000 10.99013 5.33406 22.38107 Biso 1.000 C
H1 1.0000 11.22862 6.14010 23.09572 Biso 1.000 H
H2 1.0000 11.61610 5.47921 21.48560 Biso 1.000 H
H3 1.0000 11.27052 4.38272 22.85693 Biso 1.000 H
C2 1.0000 8.85837 7.16372 21.22632 Biso 1.000 C
H4 1.0000 7.78843 7.40648 21.13213 Biso 1.000 H
H5 1.0000 9.31073 7.29852 20.23405 Biso 1.000 H
H6 1.0000 9.32075 7.88904 21.92071 Biso 1.000 H
C3 1.0000 8.22388 5.37831 23.61012 Biso 1.000 C
H7 1.0000 7.13923 5.50981 23.47985 Biso 1.000 H
H8 1.0000 8.59143 6.18981 24.26114 Biso 1.000 H
H9 1.0000 8.39221 4.42471 24.13392 Biso 1.000 H
H10 1.0000 10.00528 3.79228 20.72797 Biso 1.000 H
C4 1.0000 8.72457 1.58862 20.99745 Biso 1.000 C
C5 1.0000 9.98179 2.26871 21.04227 Biso 1.000 C
H11 1.0000 8.54183 0.95871 20.11771 Biso 1.000 H
H12 1.0000 10.75169 2.02979 20.29951 Biso 1.000 H
H13 1.0000 10.40689 2.44672 22.03630 Biso 1.000 H
C6 1.0000 8.03721 1.14380 22.25670 Biso 1.000 C
H14 1.0000 8.42453 0.14963 22.54718 Biso 1.000 H
H15 1.0000 6.95750 1.01240 22.10977 Biso 1.000 H
H16 1.0000 8.22331 1.83752 23.09005 Biso 1.000 H
H17 1.0000 7.86881 3.77286 21.80224 Biso 1.000 H

TS_RhHpropen_RhH2allyl_1x3-CeO2-211
data_image0
loop_
_atom_site_label
_atom_site_occupancy
_atom_site_Cartn_x
_atom_site_Cartn_y
_atom_site_Cartn_z

_atom_site_thermal_displace_type
_atom_site_B_iso_or_equiv
_atom_site_type_symbol
Ce1 1.0000 -0.77087 1.93685 10.23856 Biso 1.000 Ce
Ce2 1.0000 -0.75406 1.91765 16.94176 Biso 1.000 Ce
O1 1.0000 0.02059 1.93685 8.00000 Biso 1.000 O
O2 1.0000 1.60349 1.93685 10.23856 Biso 1.000 O
O3 1.0000 0.00125 1.93611 14.72844 Biso 1.000 O
Ce3 1.0000 5.56074 3.87550 9.11928 Biso 1.000 Ce
Ce4 1.0000 2.39494 1.93685 8.00000 Biso 1.000 Ce
O4 1.0000 4.76929 1.93685 8.00000 Biso 1.000 O
O5 1.0000 7.93510 3.87550 9.11928 Biso 1.000 O
O6 1.0000 3.18639 3.87550 9.11928 Biso 1.000 O
O7 1.0000 6.35219 1.93685 10.23856 Biso 1.000 O
Ce5 1.0000 2.39494 3.87550 11.35784 Biso 1.000 Ce
Ce6 1.0000 -0.78374 3.87506 13.57087 Biso 1.000 Ce
Ce7 1.0000 5.56074 1.93685 12.47712 Biso 1.000 Ce
O8 1.0000 7.93510 1.93685 12.47712 Biso 1.000 O
O9 1.0000 3.18639 1.93685 12.47712 Biso 1.000 O
O10 1.0000 4.76929 3.87550 11.35784 Biso 1.000 O
O11 1.0000 0.02059 3.87550 11.35784 Biso 1.000 O
Ce8 1.0000 2.38504 1.93558 14.69621 Biso 1.000 Ce
O12 1.0000 1.59252 3.87526 13.57280 Biso 1.000 O
O13 1.0000 6.32020 3.87571 13.57662 Biso 1.000 O
O14 1.0000 4.80288 1.94132 14.77314 Biso 1.000 O
Ce9 1.0000 5.56793 3.87808 15.85853 Biso 1.000 Ce
Ce10 1.0000 2.40478 3.86683 18.02233 Biso 1.000 Ce
O15 1.0000 1.58792 1.95576 16.91015 Biso 1.000 O
O16 1.0000 6.39365 1.86012 17.09852 Biso 1.000 O
O17 1.0000 7.91500 3.88605 15.83176 Biso 1.000 O
O18 1.0000 3.16059 3.87499 15.80029 Biso 1.000 O
Ce11 1.0000 5.37329 1.87387 19.23119 Biso 1.000 Ce
O19 1.0000 4.67667 3.88849 17.97526 Biso 1.000 O
O20 1.0000 0.07121 3.86437 18.16579 Biso 1.000 O
O21 1.0000 3.08924 1.94177 19.19455 Biso 1.000 O
O22 1.0000 6.04716 3.81390 20.31109 Biso 1.000 O
Ce12 1.0000 -0.77087 5.81416 10.23856 Biso 1.000 Ce
Ce13 1.0000 -0.74601 5.84142 16.92230 Biso 1.000 Ce
O23 1.0000 0.02059 5.81416 8.00000 Biso 1.000 O
O24 1.0000 1.60349 5.81416 10.23856 Biso 1.000 O
O25 1.0000 0.00650 5.81721 14.72461 Biso 1.000 O
Ce14 1.0000 5.56074 7.75281 9.11928 Biso 1.000 Ce
Ce15 1.0000 2.39494 5.81416 8.00000 Biso 1.000 Ce
O26 1.0000 4.76929 5.81416 8.00000 Biso 1.000 O
O27 1.0000 7.93510 7.75281 9.11928 Biso 1.000 O
O28 1.0000 3.18639 7.75281 9.11928 Biso 1.000 O
O29 1.0000 6.35219 5.81416 10.23856 Biso 1.000 O
Ce16 1.0000 2.39494 7.75281 11.35784 Biso 1.000 Ce
Ce17 1.0000 -0.78323 7.75346 13.55909 Biso 1.000 Ce
Ce18 1.0000 5.56074 5.81416 12.47712 Biso 1.000 Ce
O30 1.0000 7.93510 5.81416 12.47712 Biso 1.000 O
O31 1.0000 3.18639 5.81416 12.47712 Biso 1.000 O
O32 1.0000 4.76929 7.75281 11.35784 Biso 1.000 O
O33 1.0000 0.02059 7.75281 11.35784 Biso 1.000 O
Ce19 1.0000 2.38955 5.81529 14.69419 Biso 1.000 Ce
O34 1.0000 1.59267 7.74998 13.55791 Biso 1.000 O
O35 1.0000 6.32020 7.75301 13.57662 Biso 1.000 O
O36 1.0000 4.79466 5.80948 14.76125 Biso 1.000 O
Ce20 1.0000 5.56065 7.75932 15.85855 Biso 1.000 Ce
Ce21 1.0000 2.32204 7.74915 18.01708 Biso 1.000 Ce

O37 1.0000 1.58178 5.79398 16.91074 Biso 1.000 O
O38 1.0000 6.37001 5.93390 17.05751 Biso 1.000 O
O39 1.0000 7.92240 7.77527 15.79221 Biso 1.000 O
O40 1.0000 3.11915 7.74625 15.77834 Biso 1.000 O
Ce22 1.0000 5.35265 5.85949 19.22161 Biso 1.000 Ce
O41 1.0000 4.58208 7.78109 17.94320 Biso 1.000 O
O42 1.0000 0.00266 7.74617 18.04213 Biso 1.000 O
O43 1.0000 3.08065 5.79900 19.20549 Biso 1.000 O
O44 1.0000 5.87334 7.71220 20.20326 Biso 1.000 O
Ce23 1.0000 -0.77087 9.69146 10.23856 Biso 1.000 Ce
Ce24 1.0000 -0.72766 9.69412 16.89963 Biso 1.000 Ce
O45 1.0000 0.02059 9.69146 8.00000 Biso 1.000 O
O46 1.0000 1.60349 9.69146 10.23856 Biso 1.000 O
O47 1.0000 0.02471 9.69564 14.70798 Biso 1.000 O
Ce25 1.0000 5.56074 11.63011 9.11928 Biso 1.000 Ce
Ce26 1.0000 2.39494 9.69146 8.00000 Biso 1.000 Ce
O48 1.0000 4.76929 9.69146 8.00000 Biso 1.000 O
O49 1.0000 7.93510 11.63011 9.11928 Biso 1.000 O
O50 1.0000 3.18639 11.63011 9.11928 Biso 1.000 O
O51 1.0000 6.35219 9.69146 10.23856 Biso 1.000 O
Ce27 1.0000 2.39494 11.63011 11.35784 Biso 1.000 Ce
Ce28 1.0000 -0.78499 11.63299 13.56245 Biso 1.000 Ce
Ce29 1.0000 5.56074 9.69146 12.47712 Biso 1.000 Ce
O52 1.0000 7.93510 9.69146 12.47712 Biso 1.000 O
O53 1.0000 3.18639 9.69146 12.47712 Biso 1.000 O
O54 1.0000 4.76929 11.63011 11.35784 Biso 1.000 O
O55 1.0000 0.02059 11.63011 11.35784 Biso 1.000 O
Ce30 1.0000 2.38747 9.69312 14.68916 Biso 1.000 Ce
O56 1.0000 1.59195 11.63533 13.55767 Biso 1.000 O
O57 1.0000 6.32020 11.63032 13.57662 Biso 1.000 O
O58 1.0000 4.81117 9.69710 14.79419 Biso 1.000 O
Ce31 1.0000 5.56539 11.63411 15.86539 Biso 1.000 Ce
Ce32 1.0000 2.32462 11.63310 18.01602 Biso 1.000 Ce
O59 1.0000 1.59490 9.68646 16.90013 Biso 1.000 O
O60 1.0000 6.48003 9.68393 17.23982 Biso 1.000 O
O61 1.0000 7.90790 11.60871 15.79770 Biso 1.000 O
O62 1.0000 3.11618 11.63741 15.77871 Biso 1.000 O
Ce33 1.0000 5.27919 9.68523 19.26463 Biso 1.000 Ce
O63 1.0000 4.59194 11.59274 17.93585 Biso 1.000 O
O64 1.0000 0.01102 11.62437 18.03494 Biso 1.000 O
O65 1.0000 3.01874 9.68901 19.21283 Biso 1.000 O
O66 1.0000 5.88825 11.59267 20.20081 Biso 1.000 O
Rh1 1.0000 7.95323 3.84676 19.48826 Biso 1.000 Rh
H1 1.0000 8.26325 2.32151 20.07624 Biso 1.000 H
C1 1.0000 9.31477 4.44099 21.10079 Biso 1.000 C
C2 1.0000 8.47398 5.55874 21.00461 Biso 1.000 C
H2 1.0000 10.35748 4.56047 20.78427 Biso 1.000 H
H3 1.0000 8.88673 6.52126 20.69689 Biso 1.000 H
H4 1.0000 7.52445 5.59014 21.54330 Biso 1.000 H
C3 1.0000 8.85330 3.10654 21.40250 Biso 1.000 C
H5 1.0000 9.62192 2.33679 21.50852 Biso 1.000 H
H6 1.0000 7.98650 2.98511 22.05838 Biso 1.000 H
H7 1.0000 7.16698 4.35419 18.21095 Biso 1.000 H

Ru_1x3-CeO2-211
data_image0
loop_
_atom_site_label
_atom_site_occupancy

_atom_site_Cartn_x
_atom_site_Cartn_y
_atom_site_Cartn_z
_atom_site_thermal_displace_type
_atom_site_B_iso_or_equiv
_atom_site_type_symbol
Ce1 1.0000 -0.77087 1.93685 10.23856 Biso 1.000 Ce
Ce2 1.0000 -0.76198 1.87350 16.89638 Biso 1.000 Ce
O1 1.0000 0.02059 1.93685 8.00000 Biso 1.000 O
O2 1.0000 1.60349 1.93685 10.23856 Biso 1.000 O
O3 1.0000 0.00840 1.92656 14.72169 Biso 1.000 O
Ce3 1.0000 5.56074 3.87550 9.11928 Biso 1.000 Ce
Ce4 1.0000 2.39494 1.93685 8.00000 Biso 1.000 Ce
O4 1.0000 4.76929 1.93685 8.00000 Biso 1.000 O
O5 1.0000 7.93510 3.87550 9.11928 Biso 1.000 O
O6 1.0000 3.18639 3.87550 9.11928 Biso 1.000 O
O7 1.0000 6.35219 1.93685 10.23856 Biso 1.000 O
Ce5 1.0000 2.39494 3.87550 11.35784 Biso 1.000 Ce
Ce6 1.0000 -0.78504 3.87547 13.55184 Biso 1.000 Ce
Ce7 1.0000 5.56074 1.93685 12.47712 Biso 1.000 Ce
O8 1.0000 7.93510 1.93685 12.47712 Biso 1.000 O
O9 1.0000 3.18639 1.93685 12.47712 Biso 1.000 O
O10 1.0000 4.76929 3.87550 11.35784 Biso 1.000 O
O11 1.0000 0.02059 3.87550 11.35784 Biso 1.000 O
Ce8 1.0000 2.38834 1.94085 14.69353 Biso 1.000 Ce
O12 1.0000 1.59807 3.87551 13.60304 Biso 1.000 O
O13 1.0000 6.32020 3.87571 13.57662 Biso 1.000 O
O14 1.0000 4.80716 1.94167 14.79012 Biso 1.000 O
Ce9 1.0000 5.58689 3.87543 15.87859 Biso 1.000 Ce
Ce10 1.0000 2.44848 3.87528 18.03925 Biso 1.000 Ce
O15 1.0000 1.56579 2.00163 16.94029 Biso 1.000 O
O16 1.0000 6.39402 1.85344 17.10766 Biso 1.000 O
O17 1.0000 7.92638 3.87536 15.85378 Biso 1.000 O
O18 1.0000 3.19275 3.87545 15.83923 Biso 1.000 O
Ce11 1.0000 5.36894 1.79222 19.18744 Biso 1.000 Ce
O19 1.0000 4.71135 3.87532 18.04669 Biso 1.000 O
O20 1.0000 -0.05579 3.87539 18.30868 Biso 1.000 O
O21 1.0000 3.11562 1.91907 19.18908 Biso 1.000 O
O22 1.0000 6.35626 3.87484 20.18972 Biso 1.000 O
Ce12 1.0000 -0.77087 5.81416 10.23856 Biso 1.000 Ce
Ce13 1.0000 -0.76191 5.87722 16.89634 Biso 1.000 Ce
O23 1.0000 0.02059 5.81416 8.00000 Biso 1.000 O
O24 1.0000 1.60349 5.81416 10.23856 Biso 1.000 O
O25 1.0000 0.00835 5.82430 14.72174 Biso 1.000 O
Ce14 1.0000 5.56074 7.75281 9.11928 Biso 1.000 Ce
Ce15 1.0000 2.39494 5.81416 8.00000 Biso 1.000 Ce
O26 1.0000 4.76929 5.81416 8.00000 Biso 1.000 O
O27 1.0000 7.93510 7.75281 9.11928 Biso 1.000 O
O28 1.0000 3.18639 7.75281 9.11928 Biso 1.000 O
O29 1.0000 6.35219 5.81416 10.23856 Biso 1.000 O
Ce16 1.0000 2.39494 7.75281 11.35784 Biso 1.000 Ce
Ce17 1.0000 -0.77968 7.75264 13.53285 Biso 1.000 Ce
Ce18 1.0000 5.56074 5.81416 12.47712 Biso 1.000 Ce
O30 1.0000 7.93510 5.81416 12.47712 Biso 1.000 O
O31 1.0000 3.18639 5.81416 12.47712 Biso 1.000 O
O32 1.0000 4.76929 7.75281 11.35784 Biso 1.000 O
O33 1.0000 0.02059 7.75281 11.35784 Biso 1.000 O
Ce19 1.0000 2.38836 5.81008 14.69351 Biso 1.000 Ce
O34 1.0000 1.59544 7.74852 13.57419 Biso 1.000 O
O35 1.0000 6.32020 7.75301 13.57662 Biso 1.000 O

O36 1.0000 4.80711 5.80941 14.78991 Biso 1.000 O
Ce20 1.0000 5.56020 7.75587 15.83671 Biso 1.000 Ce
Ce21 1.0000 2.31025 7.75402 18.00834 Biso 1.000 Ce
O37 1.0000 1.56584 5.74911 16.94025 Biso 1.000 O
O38 1.0000 6.39405 5.89716 17.10756 Biso 1.000 O
O39 1.0000 7.90059 7.77291 15.78676 Biso 1.000 O
O40 1.0000 3.10777 7.73125 15.79383 Biso 1.000 O
Ce22 1.0000 5.36889 5.95799 19.18732 Biso 1.000 Ce
O41 1.0000 4.59927 7.79015 17.93277 Biso 1.000 O
O42 1.0000 0.00986 7.77160 18.01603 Biso 1.000 O
O43 1.0000 3.11560 5.83126 19.18926 Biso 1.000 O
O44 1.0000 5.90869 7.78330 20.19499 Biso 1.000 O
Ce23 1.0000 -0.77087 9.69146 10.23856 Biso 1.000 Ce
Ce24 1.0000 -0.72941 9.69133 16.88100 Biso 1.000 Ce
O45 1.0000 0.02059 9.69146 8.00000 Biso 1.000 O
O46 1.0000 1.60349 9.69146 10.23856 Biso 1.000 O
O47 1.0000 0.02096 9.69138 14.70468 Biso 1.000 O
Ce25 1.0000 5.56074 11.63011 9.11928 Biso 1.000 Ce
Ce26 1.0000 2.39494 9.69146 8.00000 Biso 1.000 Ce
O48 1.0000 4.76929 9.69146 8.00000 Biso 1.000 O
O49 1.0000 7.93510 11.63011 9.11928 Biso 1.000 O
O50 1.0000 3.18639 11.63011 9.11928 Biso 1.000 O
O51 1.0000 6.35219 9.69146 10.23856 Biso 1.000 O
Ce27 1.0000 2.39494 11.63011 11.35784 Biso 1.000 Ce
Ce28 1.0000 -0.77967 11.63024 13.53283 Biso 1.000 Ce
Ce29 1.0000 5.56074 9.69146 12.47712 Biso 1.000 Ce
O52 1.0000 7.93510 9.69146 12.47712 Biso 1.000 O
O53 1.0000 3.18639 9.69146 12.47712 Biso 1.000 O
O54 1.0000 4.76929 11.63011 11.35784 Biso 1.000 O
O55 1.0000 0.02059 11.63011 11.35784 Biso 1.000 O
Ce30 1.0000 2.37934 9.69145 14.67461 Biso 1.000 Ce
O56 1.0000 1.59542 11.63440 13.57419 Biso 1.000 O
O57 1.0000 6.32020 11.63032 13.57662 Biso 1.000 O
O58 1.0000 4.79882 9.69145 14.80248 Biso 1.000 O
Ce31 1.0000 5.56026 11.62689 15.83671 Biso 1.000 Ce
Ce32 1.0000 2.31025 11.62847 18.00829 Biso 1.000 Ce
O59 1.0000 1.59912 9.69130 16.88982 Biso 1.000 O
O60 1.0000 6.47624 9.69122 17.22176 Biso 1.000 O
O61 1.0000 7.90057 11.60975 15.78667 Biso 1.000 O
O62 1.0000 3.10778 11.65151 15.79388 Biso 1.000 O
Ce33 1.0000 5.28353 9.69116 19.24112 Biso 1.000 Ce
O63 1.0000 4.59935 11.59182 17.93280 Biso 1.000 O
O64 1.0000 0.00982 11.61074 18.01596 Biso 1.000 O
O65 1.0000 3.01916 9.69117 19.19340 Biso 1.000 O
O66 1.0000 5.90847 11.59867 20.19537 Biso 1.000 O
Ru1 1.0000 8.03881 3.87466 19.51564 Biso 1.000 Ru

Rudipropen_1x3-CeO2-211
data_image0
loop_
_atom_site_label
_atom_site_occupancy
_atom_site_Cartn_x
_atom_site_Cartn_y
_atom_site_Cartn_z
_atom_site_thermal_displace_type
_atom_site_B_iso_or_equiv
_atom_site_type_symbol
Ce1 1.0000 -0.77087 1.93685 10.23856 Biso 1.000 Ce

Ce2	1.0000	-0.74134	1.88776	16.85943	Biso	1.000	Ce
O1	1.0000	0.02059	1.93685	8.00000	Biso	1.000	O
O2	1.0000	1.60349	1.93685	10.23856	Biso	1.000	O
O3	1.0000	0.01661	1.92819	14.70725	Biso	1.000	O
Ce3	1.0000	5.56074	3.87550	9.11928	Biso	1.000	Ce
Ce4	1.0000	2.39494	1.93685	8.00000	Biso	1.000	Ce
O4	1.0000	4.76929	1.93685	8.00000	Biso	1.000	O
O5	1.0000	7.93510	3.87550	9.11928	Biso	1.000	O
O6	1.0000	3.18639	3.87550	9.11928	Biso	1.000	O
O7	1.0000	6.35219	1.93685	10.23856	Biso	1.000	O
Ce5	1.0000	2.39494	3.87550	11.35784	Biso	1.000	Ce
Ce6	1.0000	-0.78786	3.87581	13.53832	Biso	1.000	Ce
Ce7	1.0000	5.56074	1.93685	12.47712	Biso	1.000	Ce
O8	1.0000	7.93510	1.93685	12.47712	Biso	1.000	O
O9	1.0000	3.18639	1.93685	12.47712	Biso	1.000	O
O10	1.0000	4.76929	3.87550	11.35784	Biso	1.000	O
O11	1.0000	0.02059	3.87550	11.35784	Biso	1.000	O
Ce8	1.0000	2.39461	1.94506	14.68795	Biso	1.000	Ce
O12	1.0000	1.59956	3.87556	13.59670	Biso	1.000	O
O13	1.0000	6.32020	3.87571	13.57662	Biso	1.000	O
O14	1.0000	4.82824	1.94081	14.81921	Biso	1.000	O
Ce9	1.0000	5.57408	3.87643	15.88966	Biso	1.000	Ce
Ce10	1.0000	2.37183	3.88284	18.04959	Biso	1.000	Ce
O15	1.0000	1.56753	1.99161	16.92994	Biso	1.000	O
O16	1.0000	6.44655	1.90633	17.21408	Biso	1.000	O
O17	1.0000	7.92125	3.87386	15.83588	Biso	1.000	O
O18	1.0000	3.17781	3.87665	15.82790	Biso	1.000	O
Ce11	1.0000	5.30638	1.83081	19.21269	Biso	1.000	Ce
O19	1.0000	4.64759	3.87684	18.05617	Biso	1.000	O
O20	1.0000	-0.09049	3.89730	18.28319	Biso	1.000	O
O21	1.0000	3.07855	1.91249	19.20607	Biso	1.000	O
O22	1.0000	6.42772	3.86618	20.06838	Biso	1.000	O
Ce12	1.0000	-0.77087	5.81416	10.23856	Biso	1.000	Ce
Ce13	1.0000	-0.74150	5.86656	16.85852	Biso	1.000	Ce
O23	1.0000	0.02059	5.81416	8.00000	Biso	1.000	O
O24	1.0000	1.60349	5.81416	10.23856	Biso	1.000	O
O25	1.0000	0.01651	5.82311	14.70577	Biso	1.000	O
Ce14	1.0000	5.56074	7.75281	9.11928	Biso	1.000	Ce
Ce15	1.0000	2.39494	5.81416	8.00000	Biso	1.000	Ce
O26	1.0000	4.76929	5.81416	8.00000	Biso	1.000	O
O27	1.0000	7.93510	7.75281	9.11928	Biso	1.000	O
O28	1.0000	3.18639	7.75281	9.11928	Biso	1.000	O
O29	1.0000	6.35219	5.81416	10.23856	Biso	1.000	O
Ce16	1.0000	2.39494	7.75281	11.35784	Biso	1.000	Ce
Ce17	1.0000	-0.78102	7.75595	13.52614	Biso	1.000	Ce
Ce18	1.0000	5.56074	5.81416	12.47712	Biso	1.000	Ce
O30	1.0000	7.93510	5.81416	12.47712	Biso	1.000	O
O31	1.0000	3.18639	5.81416	12.47712	Biso	1.000	O
O32	1.0000	4.76929	7.75281	11.35784	Biso	1.000	O
O33	1.0000	0.02059	7.75281	11.35784	Biso	1.000	O
Ce19	1.0000	2.39554	5.80911	14.68701	Biso	1.000	Ce
O34	1.0000	1.59406	7.75132	13.57304	Biso	1.000	O
O35	1.0000	6.32020	7.75301	13.57662	Biso	1.000	O
O36	1.0000	4.82738	5.81098	14.81740	Biso	1.000	O
Ce20	1.0000	5.56298	7.75826	15.84757	Biso	1.000	Ce
Ce21	1.0000	2.30701	7.75921	17.99908	Biso	1.000	Ce
O37	1.0000	1.57421	5.76858	16.92652	Biso	1.000	O
O38	1.0000	6.44266	5.84660	17.20749	Biso	1.000	O
O39	1.0000	7.89239	7.76673	15.78557	Biso	1.000	O
O40	1.0000	3.11478	7.73845	15.78243	Biso	1.000	O

Ce22	1.0000	5.31300	5.92391	19.20885	Biso	1.000	Ce
O41	1.0000	4.59383	7.77068	17.92963	Biso	1.000	O
O42	1.0000	0.00553	7.77367	18.02529	Biso	1.000	O
O43	1.0000	3.07749	5.84172	19.21470	Biso	1.000	O
O44	1.0000	5.88670	7.79029	20.19719	Biso	1.000	O
Ce23	1.0000	-0.77087	9.69146	10.23856	Biso	1.000	Ce
Ce24	1.0000	-0.73141	9.69230	16.88410	Biso	1.000	Ce
O45	1.0000	0.02059	9.69146	8.00000	Biso	1.000	O
O46	1.0000	1.60349	9.69146	10.23856	Biso	1.000	O
O47	1.0000	0.01462	9.69261	14.70763	Biso	1.000	O
Ce25	1.0000	5.56074	11.63011	9.11928	Biso	1.000	Ce
Ce26	1.0000	2.39494	9.69146	8.00000	Biso	1.000	Ce
O48	1.0000	4.76929	9.69146	8.00000	Biso	1.000	O
O49	1.0000	7.93510	11.63011	9.11928	Biso	1.000	O
O50	1.0000	3.18639	11.63011	9.11928	Biso	1.000	O
O51	1.0000	6.35219	9.69146	10.23856	Biso	1.000	O
Ce27	1.0000	2.39494	11.63011	11.35784	Biso	1.000	Ce
Ce28	1.0000	-0.78046	11.62752	13.52599	Biso	1.000	Ce
Ce29	1.0000	5.56074	9.69146	12.47712	Biso	1.000	Ce
O52	1.0000	7.93510	9.69146	12.47712	Biso	1.000	O
O53	1.0000	3.18639	9.69146	12.47712	Biso	1.000	O
O54	1.0000	4.76929	11.63011	11.35784	Biso	1.000	O
O55	1.0000	0.02059	11.63011	11.35784	Biso	1.000	O
Ce30	1.0000	2.38039	9.69162	14.66856	Biso	1.000	Ce
O56	1.0000	1.59460	11.63291	13.57343	Biso	1.000	O
O57	1.0000	6.32020	11.63032	13.57662	Biso	1.000	O
O58	1.0000	4.81246	9.69315	14.81496	Biso	1.000	O
Ce31	1.0000	5.56501	11.62606	15.84734	Biso	1.000	Ce
Ce32	1.0000	2.30868	11.62222	17.99325	Biso	1.000	Ce
O59	1.0000	1.59363	9.69195	16.88450	Biso	1.000	O
O60	1.0000	6.47619	9.69457	17.23677	Biso	1.000	O
O61	1.0000	7.89276	11.62176	15.78582	Biso	1.000	O
O62	1.0000	3.11329	11.64816	15.78450	Biso	1.000	O
Ce33	1.0000	5.27618	9.69441	19.24671	Biso	1.000	Ce
O63	1.0000	4.59772	11.61328	17.92945	Biso	1.000	O
O64	1.0000	0.00535	11.61612	18.02170	Biso	1.000	O
O65	1.0000	3.01863	9.69303	19.18672	Biso	1.000	O
O66	1.0000	5.85583	11.61425	20.21978	Biso	1.000	O
Ru1	1.0000	8.39100	3.83364	20.05280	Biso	1.000	Ru
C1	1.0000	8.71014	4.90041	21.84753	Biso	1.000	C
C2	1.0000	9.68093	5.26856	20.84519	Biso	1.000	C
H1	1.0000	9.08905	4.23924	22.63434	Biso	1.000	H
H2	1.0000	10.73941	5.02287	20.97827	Biso	1.000	H
H3	1.0000	9.52326	6.19961	20.27924	Biso	1.000	H
C3	1.0000	7.62773	5.82800	22.32744	Biso	1.000	C
H4	1.0000	7.99881	6.41079	23.19050	Biso	1.000	H
H5	1.0000	6.74042	5.27416	22.67130	Biso	1.000	H
H6	1.0000	7.31506	6.56002	21.56694	Biso	1.000	H
C4	1.0000	8.38159	2.02134	21.13170	Biso	1.000	C
C5	1.0000	9.73394	2.42874	20.84683	Biso	1.000	C
H7	1.0000	7.96551	1.27234	20.43653	Biso	1.000	H
H8	1.0000	10.30171	1.89670	20.07852	Biso	1.000	H
H9	1.0000	10.36540	2.78018	21.67165	Biso	1.000	H
C6	1.0000	7.78755	1.87406	22.50815	Biso	1.000	C
H10	1.0000	7.83652	0.81326	22.80733	Biso	1.000	H
H11	1.0000	6.72376	2.15618	22.52476	Biso	1.000	H
H12	1.0000	8.31508	2.46576	23.26698	Biso	1.000	H

RuHallyl_1x3-CeO2-211

```
data_image0
loop_
  _atom_site_label
  _atom_site_occupancy
  _atom_site_Cartn_x
  _atom_site_Cartn_y
  _atom_site_Cartn_z
  _atom_site_thermal_displace_type
  _atom_site_B_iso_or_equiv
  _atom_site_type_symbol
  Ce1      1.0000 -0.77087  1.93685  10.23856  Biso   1.000  Ce
  Ce2      1.0000 -0.75828  1.90666  16.94005  Biso   1.000  Ce
  O1       1.0000 0.02059  1.93685  8.00000  Biso   1.000  O
  O2       1.0000 1.60349  1.93685  10.23856  Biso   1.000  O
  O3       1.0000 0.00317  1.93634  14.72208  Biso   1.000  O
  Ce3      1.0000 5.56074  3.87550  9.11928  Biso   1.000  Ce
  Ce4      1.0000 2.39494  1.93685  8.00000  Biso   1.000  Ce
  O4       1.0000 4.76929  1.93685  8.00000  Biso   1.000  O
  O5       1.0000 7.93510  3.87550  9.11928  Biso   1.000  O
  O6       1.0000 3.18639  3.87550  9.11928  Biso   1.000  O
  O7       1.0000 6.35219  1.93685  10.23856  Biso   1.000  O
  Ce5      1.0000 2.39494  3.87550  11.35784  Biso   1.000  Ce
  Ce6      1.0000 -0.78550  3.87242  13.56231  Biso   1.000  Ce
  Ce7      1.0000 5.56074  1.93685  12.47712  Biso   1.000  Ce
  O8       1.0000 7.93510  1.93685  12.47712  Biso   1.000  O
  O9       1.0000 3.18639  1.93685  12.47712  Biso   1.000  O
  O10      1.0000 4.76929  3.87550  11.35784  Biso   1.000  O
  O11      1.0000 0.02059  3.87550  11.35784  Biso   1.000  O
  Ce8      1.0000 2.38158  1.93654  14.68705  Biso   1.000  Ce
  O12      1.0000 1.59245  3.87798  13.58136  Biso   1.000  O
  O13      1.0000 6.32020  3.87571  13.57662  Biso   1.000  O
  O14      1.0000 4.80356  1.93966  14.77604  Biso   1.000  O
  Ce9      1.0000 5.57181  3.86955  15.87233  Biso   1.000  Ce
  Ce10     1.0000 2.41540  3.86305  18.01131  Biso   1.000  Ce
  O15      1.0000 1.58210  1.96139  16.91149  Biso   1.000  O
  O16      1.0000 6.37835  1.88440  17.09360  Biso   1.000  O
  O17      1.0000 7.90656  3.87944  15.85012  Biso   1.000  O
  O18      1.0000 3.16572  3.87370  15.79735  Biso   1.000  O
  Ce11     1.0000 5.36900  1.83140  19.20133  Biso   1.000  Ce
  O19      1.0000 4.65977  3.88988  17.99325  Biso   1.000  O
  O20      1.0000 0.02331  3.85991  18.15300  Biso   1.000  O
  O21      1.0000 3.08913  1.92660  19.18974  Biso   1.000  O
  O22      1.0000 6.18799  3.86609  20.12809  Biso   1.000  O
  Ce12     1.0000 -0.77087  5.81416  10.23856  Biso   1.000  Ce
  Ce13     1.0000 -0.73923  5.84956  16.90954  Biso   1.000  Ce
  O23      1.0000 0.02059  5.81416  8.00000  Biso   1.000  O
  O24      1.0000 1.60349  5.81416  10.23856  Biso   1.000  O
  O25      1.0000 0.00954  5.81760  14.72277  Biso   1.000  O
  Ce14     1.0000 5.56074  7.75281  9.11928  Biso   1.000  Ce
  Ce15     1.0000 2.39494  5.81416  8.00000  Biso   1.000  Ce
  O26      1.0000 4.76929  5.81416  8.00000  Biso   1.000  O
  O27      1.0000 7.93510  7.75281  9.11928  Biso   1.000  O
  O28      1.0000 3.18639  7.75281  9.11928  Biso   1.000  O
  O29      1.0000 6.35219  5.81416  10.23856  Biso   1.000  O
  Ce16     1.0000 2.39494  7.75281  11.35784  Biso   1.000  Ce
  Ce17     1.0000 -0.78364  7.75258  13.54488  Biso   1.000  Ce
  Ce18     1.0000 5.56074  5.81416  12.47712  Biso   1.000  Ce
  O30      1.0000 7.93510  5.81416  12.47712  Biso   1.000  O
  O31      1.0000 3.18639  5.81416  12.47712  Biso   1.000  O
  O32      1.0000 4.76929  7.75281  11.35784  Biso   1.000  O
```

O33	1.0000	0.02059	7.75281	11.35784	Biso	1.000	O
Ce19	1.0000	2.38939	5.81469	14.69172	Biso	1.000	Ce
O34	1.0000	1.59243	7.74956	13.56640	Biso	1.000	O
O35	1.0000	6.32020	7.75301	13.57662	Biso	1.000	O
O36	1.0000	4.82379	5.81551	14.80479	Biso	1.000	O
Ce20	1.0000	5.57242	7.75578	15.85810	Biso	1.000	Ce
Ce21	1.0000	2.31276	7.76412	17.99975	Biso	1.000	Ce
O37	1.0000	1.59052	5.79015	16.91564	Biso	1.000	O
O38	1.0000	6.43548	5.86628	17.19814	Biso	1.000	O
O39	1.0000	7.90025	7.76197	15.79266	Biso	1.000	O
O40	1.0000	3.12371	7.74597	15.77807	Biso	1.000	O
Ce22	1.0000	5.31865	5.94658	19.23365	Biso	1.000	Ce
O41	1.0000	4.59844	7.78075	17.93262	Biso	1.000	O
O42	1.0000	0.01074	7.76991	18.03734	Biso	1.000	O
O43	1.0000	3.07680	5.83942	19.20627	Biso	1.000	O
O44	1.0000	5.80455	7.79714	20.25020	Biso	1.000	O
Ce23	1.0000	-0.77087	9.69146	10.23856	Biso	1.000	Ce
Ce24	1.0000	-0.73045	9.69687	16.89134	Biso	1.000	Ce
O45	1.0000	0.02059	9.69146	8.00000	Biso	1.000	O
O46	1.0000	1.60349	9.69146	10.23856	Biso	1.000	O
O47	1.0000	0.01662	9.69119	14.70908	Biso	1.000	O
Ce25	1.0000	5.56074	11.63011	9.11928	Biso	1.000	Ce
Ce26	1.0000	2.39494	9.69146	8.00000	Biso	1.000	Ce
O48	1.0000	4.76929	9.69146	8.00000	Biso	1.000	O
O49	1.0000	7.93510	11.63011	9.11928	Biso	1.000	O
O50	1.0000	3.18639	11.63011	9.11928	Biso	1.000	O
O51	1.0000	6.35219	9.69146	10.23856	Biso	1.000	O
Ce27	1.0000	2.39494	11.63011	11.35784	Biso	1.000	Ce
Ce28	1.0000	-0.78275	11.63455	13.55027	Biso	1.000	Ce
Ce29	1.0000	5.56074	9.69146	12.47712	Biso	1.000	Ce
O52	1.0000	7.93510	9.69146	12.47712	Biso	1.000	O
O53	1.0000	3.18639	9.69146	12.47712	Biso	1.000	O
O54	1.0000	4.76929	11.63011	11.35784	Biso	1.000	O
O55	1.0000	0.02059	11.63011	11.35784	Biso	1.000	O
Ce30	1.0000	2.38541	9.69192	14.67936	Biso	1.000	Ce
O56	1.0000	1.59055	11.63208	13.56514	Biso	1.000	O
O57	1.0000	6.32020	11.63032	13.57662	Biso	1.000	O
O58	1.0000	4.81857	9.68662	14.80386	Biso	1.000	O
Ce31	1.0000	5.56217	11.63251	15.84283	Biso	1.000	Ce
Ce32	1.0000	2.31473	11.63639	18.00518	Biso	1.000	Ce
O59	1.0000	1.59665	9.69179	16.88656	Biso	1.000	O
O60	1.0000	6.48316	9.68605	17.24306	Biso	1.000	O
O61	1.0000	7.89831	11.61771	15.79613	Biso	1.000	O
O62	1.0000	3.12134	11.63855	15.77644	Biso	1.000	O
Ce33	1.0000	5.26523	9.70859	19.26044	Biso	1.000	Ce
O63	1.0000	4.60230	11.60297	17.92819	Biso	1.000	O
O64	1.0000	0.01281	11.61082	18.01810	Biso	1.000	O
O65	1.0000	3.01064	9.69978	19.19125	Biso	1.000	O
O66	1.0000	5.85151	11.61224	20.22439	Biso	1.000	O
Ru1	1.0000	8.05535	3.88947	19.58068	Biso	1.000	Ru
H1	1.0000	7.86218	2.23654	19.25142	Biso	1.000	H
C1	1.0000	9.45716	5.08406	20.67722	Biso	1.000	C
C2	1.0000	8.28373	5.86145	20.73794	Biso	1.000	C
H2	1.0000	10.32373	5.47731	20.13288	Biso	1.000	H
H3	1.0000	8.29594	6.85933	20.29314	Biso	1.000	H
H4	1.0000	7.56420	5.71719	21.55016	Biso	1.000	H
C3	1.0000	9.46431	3.71420	21.11905	Biso	1.000	C
H5	1.0000	10.36059	3.10731	20.94813	Biso	1.000	H
H6	1.0000	8.89173	3.41745	22.00568	Biso	1.000	H

```
RuHSiMe3_1x3-CeO2-211
data_image0
loop_
  _atom_site_label
  _atom_site_occupancy
  _atom_site_Cartn_x
  _atom_site_Cartn_y
  _atom_site_Cartn_z
  _atom_site_thermal_displace_type
  _atom_site_B_iso_or_equiv
  _atom_site_type_symbol
  Ce1      1.0000 -0.77087  1.93685  10.23856  Biso   1.000  Ce
  Ce2      1.0000 -0.73695  1.88659  16.91089  Biso   1.000  Ce
  O1       1.0000 0.02059  1.93685  8.00000  Biso   1.000  O
  O2       1.0000 1.60349  1.93685  10.23856  Biso   1.000  O
  O3       1.0000 0.00922  1.93040  14.71586  Biso   1.000  O
  Ce3      1.0000 5.56074  3.87550  9.11928  Biso   1.000  Ce
  Ce4      1.0000 2.39494  1.93685  8.00000  Biso   1.000  Ce
  O4       1.0000 4.76929  1.93685  8.00000  Biso   1.000  O
  O5       1.0000 7.93510  3.87550  9.11928  Biso   1.000  O
  O6       1.0000 3.18639  3.87550  9.11928  Biso   1.000  O
  O7       1.0000 6.35219  1.93685  10.23856  Biso   1.000  O
  Ce5      1.0000 2.39494  3.87550  11.35784  Biso   1.000  Ce
  Ce6      1.0000 -0.78962  3.87305  13.55340  Biso   1.000  Ce
  Ce7      1.0000 5.56074  1.93685  12.47712  Biso   1.000  Ce
  O8       1.0000 7.93510  1.93685  12.47712  Biso   1.000  O
  O9       1.0000 3.18639  1.93685  12.47712  Biso   1.000  O
  O10      1.0000 4.76929  3.87550  11.35784  Biso   1.000  O
  O11      1.0000 0.02059  3.87550  11.35784  Biso   1.000  O
  Ce8      1.0000 2.38796  1.93883  14.68724  Biso   1.000  Ce
  O12      1.0000 1.59590  3.87521  13.58736  Biso   1.000  O
  O13      1.0000 6.32020  3.87571  13.57662  Biso   1.000  O
  O14      1.0000 4.81907  1.93987  14.80090  Biso   1.000  O
  Ce9      1.0000 5.57153  3.86765  15.89600  Biso   1.000  Ce
  Ce10     1.0000 2.37862  3.86524  18.02696  Biso   1.000  Ce
  O15      1.0000 1.58272  1.95736  16.91828  Biso   1.000  O
  O16      1.0000 6.40254  1.92286  17.16530  Biso   1.000  O
  O17      1.0000 7.90092  3.88514  15.83578  Biso   1.000  O
  O18      1.0000 3.16655  3.86875  15.80876  Biso   1.000  O
  Ce11     1.0000 5.33289  1.80531  19.23138  Biso   1.000  Ce
  O19      1.0000 4.62615  3.91038  18.03237  Biso   1.000  O
  O20      1.0000 0.00685  3.91256  18.14972  Biso   1.000  O
  O21      1.0000 3.09684  1.87541  19.18016  Biso   1.000  O
  O22      1.0000 6.25578  3.89644  20.13602  Biso   1.000  O
  Ce12     1.0000 -0.77087  5.81416  10.23856  Biso   1.000  Ce
  Ce13     1.0000 -0.73464  5.85884  16.87538  Biso   1.000  Ce
  O23      1.0000 0.02059  5.81416  8.00000  Biso   1.000  O
  O24      1.0000 1.60349  5.81416  10.23856  Biso   1.000  O
  O25      1.0000 0.01545  5.82005  14.70901  Biso   1.000  O
  Ce14     1.0000 5.56074  7.75281  9.11928  Biso   1.000  Ce
  Ce15     1.0000 2.39494  5.81416  8.00000  Biso   1.000  Ce
  O26      1.0000 4.76929  5.81416  8.00000  Biso   1.000  O
  O27      1.0000 7.93510  7.75281  9.11928  Biso   1.000  O
  O28      1.0000 3.18639  7.75281  9.11928  Biso   1.000  O
  O29      1.0000 6.35219  5.81416  10.23856  Biso   1.000  O
  Ce16     1.0000 2.39494  7.75281  11.35784  Biso   1.000  Ce
  Ce17     1.0000 -0.78238  7.75407  13.53372  Biso   1.000  Ce
  Ce18     1.0000 5.56074  5.81416  12.47712  Biso   1.000  Ce
  O30      1.0000 7.93510  5.81416  12.47712  Biso   1.000  O
```

O31	1.0000	3.18639	5.81416	12.47712	Biso	1.000	O
O32	1.0000	4.76929	7.75281	11.35784	Biso	1.000	O
O33	1.0000	0.02059	7.75281	11.35784	Biso	1.000	O
Ce19	1.0000	2.39578	5.81143	14.68977	Biso	1.000	Ce
O34	1.0000	1.59316	7.75147	13.56795	Biso	1.000	O
O35	1.0000	6.32020	7.75301	13.57662	Biso	1.000	O
O36	1.0000	4.83578	5.81199	14.82681	Biso	1.000	O
Ce20	1.0000	5.56846	7.75974	15.85754	Biso	1.000	Ce
Ce21	1.0000	2.29316	7.76543	17.99873	Biso	1.000	Ce
O37	1.0000	1.59287	5.79399	16.90452	Biso	1.000	O
O38	1.0000	6.45329	5.86042	17.25738	Biso	1.000	O
O39	1.0000	7.89423	7.75727	15.78329	Biso	1.000	O
O40	1.0000	3.12920	7.74648	15.77172	Biso	1.000	O
Ce22	1.0000	5.24954	5.95664	19.22969	Biso	1.000	Ce
O41	1.0000	4.58320	7.78200	17.93549	Biso	1.000	O
O42	1.0000	-0.00147	7.76593	18.03032	Biso	1.000	O
O43	1.0000	3.02974	5.86984	19.22103	Biso	1.000	O
O44	1.0000	5.83023	7.75578	20.24357	Biso	1.000	O
Ce23	1.0000	-0.77087	9.69146	10.23856	Biso	1.000	Ce
Ce24	1.0000	-0.73386	9.69147	16.88850	Biso	1.000	Ce
O45	1.0000	0.02059	9.69146	8.00000	Biso	1.000	O
O46	1.0000	1.60349	9.69146	10.23856	Biso	1.000	O
O47	1.0000	0.01284	9.69304	14.70729	Biso	1.000	O
Ce25	1.0000	5.56074	11.63011	9.11928	Biso	1.000	Ce
Ce26	1.0000	2.39494	9.69146	8.00000	Biso	1.000	Ce
O48	1.0000	4.76929	9.69146	8.00000	Biso	1.000	O
O49	1.0000	7.93510	11.63011	9.11928	Biso	1.000	O
O50	1.0000	3.18639	11.63011	9.11928	Biso	1.000	O
O51	1.0000	6.35219	9.69146	10.23856	Biso	1.000	O
Ce27	1.0000	2.39494	11.63011	11.35784	Biso	1.000	Ce
Ce28	1.0000	-0.78228	11.63171	13.54016	Biso	1.000	Ce
Ce29	1.0000	5.56074	9.69146	12.47712	Biso	1.000	Ce
O52	1.0000	7.93510	9.69146	12.47712	Biso	1.000	O
O53	1.0000	3.18639	9.69146	12.47712	Biso	1.000	O
O54	1.0000	4.76929	11.63011	11.35784	Biso	1.000	O
O55	1.0000	0.02059	11.63011	11.35784	Biso	1.000	O
Ce30	1.0000	2.38509	9.68930	14.67285	Biso	1.000	Ce
O56	1.0000	1.59262	11.63146	13.56906	Biso	1.000	O
O57	1.0000	6.32020	11.63032	13.57662	Biso	1.000	O
O58	1.0000	4.82464	9.68988	14.81173	Biso	1.000	O
Ce31	1.0000	5.56433	11.63274	15.84801	Biso	1.000	Ce
Ce32	1.0000	2.31963	11.61834	17.99922	Biso	1.000	Ce
O59	1.0000	1.59487	9.68313	16.87912	Biso	1.000	O
O60	1.0000	6.48430	9.67287	17.24798	Biso	1.000	O
O61	1.0000	7.88617	11.61897	15.79777	Biso	1.000	O
O62	1.0000	3.12642	11.63686	15.77817	Biso	1.000	O
Ce33	1.0000	5.27148	9.69417	19.26324	Biso	1.000	Ce
O63	1.0000	4.61998	11.58876	17.92884	Biso	1.000	O
O64	1.0000	0.01754	11.58897	18.01405	Biso	1.000	O
O65	1.0000	3.01436	9.69613	19.19267	Biso	1.000	O
O66	1.0000	5.84338	11.57728	20.24494	Biso	1.000	O
Ru1	1.0000	7.98053	3.59538	19.42205	Biso	1.000	Ru
Si1	1.0000	9.11217	4.02975	21.44406	Biso	1.000	Si
C1	1.0000	8.23388	3.11006	22.83795	Biso	1.000	C
H1	1.0000	8.69390	3.35602	23.80959	Biso	1.000	H
H2	1.0000	8.29036	2.02100	22.69486	Biso	1.000	H
H3	1.0000	7.17239	3.39488	22.86782	Biso	1.000	H
C2	1.0000	10.96132	3.64507	21.50317	Biso	1.000	C
H4	1.0000	11.53508	4.42031	20.96632	Biso	1.000	H
H5	1.0000	11.22384	2.65600	21.10256	Biso	1.000	H

H6 1.0000 11.29658 3.68202 22.55381 Biso 1.000 H
C3 1.0000 8.92423 5.90630 21.64752 Biso 1.000 C
H7 1.0000 9.37977 6.44869 20.80403 Biso 1.000 H
H8 1.0000 9.44647 6.22681 22.56610 Biso 1.000 H
H9 1.0000 7.87559 6.22997 21.72240 Biso 1.000 H
H10 1.0000 8.57327 2.12079 19.56244 Biso 1.000 H

RuHSiMe3propen_1x3-CeO2-211
data_image0
loop_
_atom_site_label
_atom_site_occupancy
_atom_site_Cartn_x
_atom_site_Cartn_y
_atom_site_Cartn_z
_atom_site_thermal_displace_type
_atom_site_B_iso_or_equiv
_atom_site_type_symbol
Ce1 1.0000 -0.77087 1.93685 10.23856 Biso 1.000 Ce
Ce2 1.0000 -0.73550 1.88996 16.85399 Biso 1.000 Ce
O1 1.0000 0.02059 1.93685 8.00000 Biso 1.000 O
O2 1.0000 1.60349 1.93685 10.23856 Biso 1.000 O
O3 1.0000 0.02140 1.92774 14.70820 Biso 1.000 O
Ce3 1.0000 5.56074 3.87550 9.11928 Biso 1.000 Ce
Ce4 1.0000 2.39494 1.93685 8.00000 Biso 1.000 Ce
O4 1.0000 4.76929 1.93685 8.00000 Biso 1.000 O
O5 1.0000 7.93510 3.87550 9.11928 Biso 1.000 O
O6 1.0000 3.18639 3.87550 9.11928 Biso 1.000 O
O7 1.0000 6.35219 1.93685 10.23856 Biso 1.000 O
Ce5 1.0000 2.39494 3.87550 11.35784 Biso 1.000 Ce
Ce6 1.0000 -0.78752 3.87586 13.53753 Biso 1.000 Ce
Ce7 1.0000 5.56074 1.93685 12.47712 Biso 1.000 Ce
O8 1.0000 7.93510 1.93685 12.47712 Biso 1.000 O
O9 1.0000 3.18639 1.93685 12.47712 Biso 1.000 O
O10 1.0000 4.76929 3.87550 11.35784 Biso 1.000 O
O11 1.0000 0.02059 3.87550 11.35784 Biso 1.000 O
Ce8 1.0000 2.39824 1.94671 14.69081 Biso 1.000 Ce
O12 1.0000 1.60145 3.87533 13.59832 Biso 1.000 O
O13 1.0000 6.32020 3.87571 13.57662 Biso 1.000 O
O14 1.0000 4.82811 1.93999 14.81725 Biso 1.000 O
Ce9 1.0000 5.57172 3.87672 15.88651 Biso 1.000 Ce
Ce10 1.0000 2.36486 3.88211 18.07330 Biso 1.000 Ce
O15 1.0000 1.57193 1.98763 16.93590 Biso 1.000 O
O16 1.0000 6.45461 1.90612 17.20989 Biso 1.000 O
O17 1.0000 7.91822 3.86505 15.82406 Biso 1.000 O
O18 1.0000 3.17632 3.87626 15.83489 Biso 1.000 O
Ce11 1.0000 5.32251 1.84034 19.20428 Biso 1.000 Ce
O19 1.0000 4.64540 3.86720 18.05889 Biso 1.000 O
O20 1.0000 -0.11182 3.86301 18.24297 Biso 1.000 O
O21 1.0000 3.09145 1.91298 19.21577 Biso 1.000 O
O22 1.0000 6.51834 3.89793 19.98543 Biso 1.000 O
Ce12 1.0000 -0.77087 5.81416 10.23856 Biso 1.000 Ce
Ce13 1.0000 -0.74193 5.85777 16.86080 Biso 1.000 Ce
O23 1.0000 0.02059 5.81416 8.00000 Biso 1.000 O
O24 1.0000 1.60349 5.81416 10.23856 Biso 1.000 O
O25 1.0000 0.01919 5.82095 14.71039 Biso 1.000 O
Ce14 1.0000 5.56074 7.75281 9.11928 Biso 1.000 Ce
Ce15 1.0000 2.39494 5.81416 8.00000 Biso 1.000 Ce
O26 1.0000 4.76929 5.81416 8.00000 Biso 1.000 O

O27	1.0000	7.93510	7.75281	9.11928	Biso	1.000	O
O28	1.0000	3.18639	7.75281	9.11928	Biso	1.000	O
O29	1.0000	6.35219	5.81416	10.23856	Biso	1.000	O
Ce16	1.0000	2.39494	7.75281	11.35784	Biso	1.000	Ce
Ce17	1.0000	-0.78097	7.75411	13.52796	Biso	1.000	Ce
Ce18	1.0000	5.56074	5.81416	12.47712	Biso	1.000	Ce
O30	1.0000	7.93510	5.81416	12.47712	Biso	1.000	O
O31	1.0000	3.18639	5.81416	12.47712	Biso	1.000	O
O32	1.0000	4.76929	7.75281	11.35784	Biso	1.000	O
O33	1.0000	0.02059	7.75281	11.35784	Biso	1.000	O
Ce19	1.0000	2.39600	5.80616	14.69285	Biso	1.000	Ce
O34	1.0000	1.59628	7.75046	13.57751	Biso	1.000	O
O35	1.0000	6.32020	7.75301	13.57662	Biso	1.000	O
O36	1.0000	4.82252	5.81075	14.81134	Biso	1.000	O
Ce20	1.0000	5.56261	7.75488	15.84754	Biso	1.000	Ce
Ce21	1.0000	2.31192	7.75551	18.00736	Biso	1.000	Ce
O37	1.0000	1.57718	5.76174	16.94552	Biso	1.000	O
O38	1.0000	6.43342	5.84010	17.19044	Biso	1.000	O
O39	1.0000	7.88454	7.76370	15.78077	Biso	1.000	O
O40	1.0000	3.11482	7.73470	15.79296	Biso	1.000	O
Ce22	1.0000	5.34223	5.91068	19.20767	Biso	1.000	Ce
O41	1.0000	4.60227	7.76880	17.93792	Biso	1.000	O
O42	1.0000	0.00795	7.78477	17.98129	Biso	1.000	O
O43	1.0000	3.09103	5.83278	19.22785	Biso	1.000	O
O44	1.0000	5.92300	7.76847	20.19076	Biso	1.000	O
Ce23	1.0000	-0.77087	9.69146	10.23856	Biso	1.000	Ce
Ce24	1.0000	-0.72582	9.69188	16.87747	Biso	1.000	Ce
O45	1.0000	0.02059	9.69146	8.00000	Biso	1.000	O
O46	1.0000	1.60349	9.69146	10.23856	Biso	1.000	O
O47	1.0000	0.01729	9.69146	14.70743	Biso	1.000	O
Ce25	1.0000	5.56074	11.63011	9.11928	Biso	1.000	Ce
Ce26	1.0000	2.39494	9.69146	8.00000	Biso	1.000	Ce
O48	1.0000	4.76929	9.69146	8.00000	Biso	1.000	O
O49	1.0000	7.93510	11.63011	9.11928	Biso	1.000	O
O50	1.0000	3.18639	11.63011	9.11928	Biso	1.000	O
O51	1.0000	6.35219	9.69146	10.23856	Biso	1.000	O
Ce27	1.0000	2.39494	11.63011	11.35784	Biso	1.000	Ce
Ce28	1.0000	-0.77888	11.62724	13.52438	Biso	1.000	Ce
Ce29	1.0000	5.56074	9.69146	12.47712	Biso	1.000	Ce
O52	1.0000	7.93510	9.69146	12.47712	Biso	1.000	O
O53	1.0000	3.18639	9.69146	12.47712	Biso	1.000	O
O54	1.0000	4.76929	11.63011	11.35784	Biso	1.000	O
O55	1.0000	0.02059	11.63011	11.35784	Biso	1.000	O
Ce30	1.0000	2.38319	9.69057	14.67154	Biso	1.000	Ce
O56	1.0000	1.59593	11.63232	13.57647	Biso	1.000	O
O57	1.0000	6.32020	11.63032	13.57662	Biso	1.000	O
O58	1.0000	4.81170	9.69133	14.81379	Biso	1.000	O
Ce31	1.0000	5.56602	11.62480	15.84610	Biso	1.000	Ce
Ce32	1.0000	2.31767	11.62419	17.99599	Biso	1.000	Ce
O59	1.0000	1.60488	9.69838	16.88961	Biso	1.000	O
O60	1.0000	6.47643	9.69476	17.23682	Biso	1.000	O
O61	1.0000	7.89430	11.62416	15.78340	Biso	1.000	O
O62	1.0000	3.12044	11.64902	15.78754	Biso	1.000	O
Ce33	1.0000	5.28896	9.68703	19.24447	Biso	1.000	Ce
O63	1.0000	4.60692	11.61954	17.93162	Biso	1.000	O
O64	1.0000	0.00672	11.62414	18.01470	Biso	1.000	O
O65	1.0000	3.03767	9.69218	19.19031	Biso	1.000	O
O66	1.0000	5.87481	11.62579	20.20502	Biso	1.000	O
Ru1	1.0000	8.49605	3.79896	20.10173	Biso	1.000	Ru
Si1	1.0000	8.99517	5.34665	21.87081	Biso	1.000	Si

C1 1.0000 10.33334 4.84248 23.11600 Biso 1.000 C
H1 1.0000 10.44874 5.66508 23.84146 Biso 1.000 H
H2 1.0000 11.30372 4.70670 22.61323 Biso 1.000 H
H3 1.0000 10.11268 3.92882 23.68469 Biso 1.000 H
C2 1.0000 9.60318 6.98837 21.15950 Biso 1.000 C
H4 1.0000 8.98888 7.36471 20.33116 Biso 1.000 H
H5 1.0000 10.64240 6.86846 20.81168 Biso 1.000 H
H6 1.0000 9.59458 7.74552 21.96133 Biso 1.000 H
C3 1.0000 7.37635 5.68955 22.80008 Biso 1.000 C
H7 1.0000 6.74251 6.35994 22.19773 Biso 1.000 H
H8 1.0000 7.60825 6.20960 23.74508 Biso 1.000 H
H9 1.0000 6.81424 4.77687 23.03723 Biso 1.000 H
H10 1.0000 9.97168 4.36532 20.45560 Biso 1.000 H
C4 1.0000 8.35805 2.03099 21.26068 Biso 1.000 C
C5 1.0000 9.72660 2.25199 20.89290 Biso 1.000 C
H11 1.0000 7.81995 1.30003 20.63501 Biso 1.000 H
H12 1.0000 10.15702 1.65339 20.08453 Biso 1.000 H
H13 1.0000 10.45397 2.52235 21.66100 Biso 1.000 H
C6 1.0000 7.83779 2.03346 22.67388 Biso 1.000 C
H14 1.0000 7.89528 1.01088 23.08682 Biso 1.000 H
H15 1.0000 6.78011 2.33417 22.71411 Biso 1.000 H
H16 1.0000 8.40910 2.69967 23.33274 Biso 1.000 H

Rupropen_1x3-CeO2-211
data_image0
loop_
_atom_site_label
_atom_site_occupancy
_atom_site_Cartn_x
_atom_site_Cartn_y
_atom_site_Cartn_z
_atom_site_thermal_displace_type
_atom_site_B_iso_or_equiv
_atom_site_type_symbol
Ce1 1.0000 -0.77087 1.93685 10.23856 Biso 1.000 Ce
Ce2 1.0000 -0.72796 1.86862 16.84310 Biso 1.000 Ce
O1 1.0000 0.02059 1.93685 8.00000 Biso 1.000 O
O2 1.0000 1.60349 1.93685 10.23856 Biso 1.000 O
O3 1.0000 0.02324 1.92563 14.69807 Biso 1.000 O
Ce3 1.0000 5.56074 3.87550 9.11928 Biso 1.000 Ce
Ce4 1.0000 2.39494 1.93685 8.00000 Biso 1.000 Ce
O4 1.0000 4.76929 1.93685 8.00000 Biso 1.000 O
O5 1.0000 7.93510 3.87550 9.11928 Biso 1.000 O
O6 1.0000 3.18639 3.87550 9.11928 Biso 1.000 O
O7 1.0000 6.35219 1.93685 10.23856 Biso 1.000 O
Ce5 1.0000 2.39494 3.87550 11.35784 Biso 1.000 Ce
Ce6 1.0000 -0.78737 3.88072 13.54871 Biso 1.000 Ce
Ce7 1.0000 5.56074 1.93685 12.47712 Biso 1.000 Ce
O8 1.0000 7.93510 1.93685 12.47712 Biso 1.000 O
O9 1.0000 3.18639 1.93685 12.47712 Biso 1.000 O
O10 1.0000 4.76929 3.87550 11.35784 Biso 1.000 O
O11 1.0000 0.02059 3.87550 11.35784 Biso 1.000 O
Ce8 1.0000 2.40210 1.94256 14.68789 Biso 1.000 Ce
O12 1.0000 1.59645 3.87549 13.58934 Biso 1.000 O
O13 1.0000 6.32020 3.87571 13.57662 Biso 1.000 O
O14 1.0000 4.84047 1.93663 14.83107 Biso 1.000 O
Ce9 1.0000 5.57450 3.89238 15.88716 Biso 1.000 Ce
Ce10 1.0000 2.39967 3.88322 18.03663 Biso 1.000 Ce
O15 1.0000 1.57699 1.97484 16.90934 Biso 1.000 O

O16	1.0000	6.49805	1.90014	17.26653	Biso	1.000	O
O17	1.0000	7.91545	3.84849	15.84087	Biso	1.000	O
O18	1.0000	3.17447	3.87861	15.81107	Biso	1.000	O
Ce11	1.0000	5.29466	1.82675	19.23825	Biso	1.000	Ce
O19	1.0000	4.65324	3.84212	18.03197	Biso	1.000	O
O20	1.0000	-0.01713	3.84719	18.24658	Biso	1.000	O
O21	1.0000	3.05593	1.90795	19.22210	Biso	1.000	O
O22	1.0000	6.35650	3.91936	20.11388	Biso	1.000	O
Ce12	1.0000	-0.77087	5.81416	10.23856	Biso	1.000	Ce
Ce13	1.0000	-0.75977	5.84741	16.92138	Biso	1.000	Ce
O23	1.0000	0.02059	5.81416	8.00000	Biso	1.000	O
O24	1.0000	1.60349	5.81416	10.23856	Biso	1.000	O
O25	1.0000	0.00219	5.81783	14.71992	Biso	1.000	O
Ce14	1.0000	5.56074	7.75281	9.11928	Biso	1.000	Ce
Ce15	1.0000	2.39494	5.81416	8.00000	Biso	1.000	Ce
O26	1.0000	4.76929	5.81416	8.00000	Biso	1.000	O
O27	1.0000	7.93510	7.75281	9.11928	Biso	1.000	O
O28	1.0000	3.18639	7.75281	9.11928	Biso	1.000	O
O29	1.0000	6.35219	5.81416	10.23856	Biso	1.000	O
Ce16	1.0000	2.39494	7.75281	11.35784	Biso	1.000	Ce
Ce17	1.0000	-0.78157	7.74920	13.54310	Biso	1.000	Ce
Ce18	1.0000	5.56074	5.81416	12.47712	Biso	1.000	Ce
O30	1.0000	7.93510	5.81416	12.47712	Biso	1.000	O
O31	1.0000	3.18639	5.81416	12.47712	Biso	1.000	O
O32	1.0000	4.76929	7.75281	11.35784	Biso	1.000	O
O33	1.0000	0.02059	7.75281	11.35784	Biso	1.000	O
Ce19	1.0000	2.38313	5.81170	14.69143	Biso	1.000	Ce
O34	1.0000	1.59168	7.75018	13.57073	Biso	1.000	O
O35	1.0000	6.32020	7.75301	13.57662	Biso	1.000	O
O36	1.0000	4.80599	5.81129	14.78308	Biso	1.000	O
Ce20	1.0000	5.56057	7.75078	15.84191	Biso	1.000	Ce
Ce21	1.0000	2.31845	7.75998	18.00015	Biso	1.000	Ce
O37	1.0000	1.57649	5.78202	16.93042	Biso	1.000	O
O38	1.0000	6.36922	5.85163	17.10532	Biso	1.000	O
O39	1.0000	7.88981	7.77477	15.79815	Biso	1.000	O
O40	1.0000	3.11841	7.74170	15.78074	Biso	1.000	O
Ce22	1.0000	5.36031	5.95319	19.20847	Biso	1.000	Ce
O41	1.0000	4.61174	7.80219	17.92800	Biso	1.000	O
O42	1.0000	0.02081	7.80140	18.02854	Biso	1.000	O
O43	1.0000	3.09862	5.85034	19.19921	Biso	1.000	O
O44	1.0000	5.84540	7.85503	20.23798	Biso	1.000	O
Ce23	1.0000	-0.77087	9.69146	10.23856	Biso	1.000	Ce
Ce24	1.0000	-0.72864	9.68201	16.88386	Biso	1.000	Ce
O45	1.0000	0.02059	9.69146	8.00000	Biso	1.000	O
O46	1.0000	1.60349	9.69146	10.23856	Biso	1.000	O
O47	1.0000	0.01733	9.69136	14.70431	Biso	1.000	O
Ce25	1.0000	5.56074	11.63011	9.11928	Biso	1.000	Ce
Ce26	1.0000	2.39494	9.69146	8.00000	Biso	1.000	Ce
O48	1.0000	4.76929	9.69146	8.00000	Biso	1.000	O
O49	1.0000	7.93510	11.63011	9.11928	Biso	1.000	O
O50	1.0000	3.18639	11.63011	9.11928	Biso	1.000	O
O51	1.0000	6.35219	9.69146	10.23856	Biso	1.000	O
Ce27	1.0000	2.39494	11.63011	11.35784	Biso	1.000	Ce
Ce28	1.0000	-0.78073	11.62460	13.52460	Biso	1.000	Ce
Ce29	1.0000	5.56074	9.69146	12.47712	Biso	1.000	Ce
O52	1.0000	7.93510	9.69146	12.47712	Biso	1.000	O
O53	1.0000	3.18639	9.69146	12.47712	Biso	1.000	O
O54	1.0000	4.76929	11.63011	11.35784	Biso	1.000	O
O55	1.0000	0.02059	11.63011	11.35784	Biso	1.000	O
Ce30	1.0000	2.38410	9.69315	14.67403	Biso	1.000	Ce

O56 1.0000 1.59811 11.63162 13.56864 Biso 1.000 O
O57 1.0000 6.32020 11.63032 13.57662 Biso 1.000 O
O58 1.0000 4.81701 9.69955 14.81079 Biso 1.000 O
Ce31 1.0000 5.56902 11.62133 15.85749 Biso 1.000 Ce
Ce32 1.0000 2.30711 11.62901 18.00339 Biso 1.000 Ce
O59 1.0000 1.60019 9.70164 16.88057 Biso 1.000 O
O60 1.0000 6.48240 9.72077 17.23843 Biso 1.000 O
O61 1.0000 7.89764 11.62700 15.77247 Biso 1.000 O
O62 1.0000 3.12687 11.64525 15.78103 Biso 1.000 O
Ce33 1.0000 5.27496 9.70458 19.25884 Biso 1.000 Ce
O63 1.0000 4.59748 11.63211 17.93636 Biso 1.000 O
O64 1.0000 0.00099 11.62661 18.00621 Biso 1.000 O
O65 1.0000 3.01553 9.70973 19.18742 Biso 1.000 O
O66 1.0000 5.86010 11.66452 20.23388 Biso 1.000 O
Ru1 1.0000 8.10635 4.37867 19.55944 Biso 1.000 Ru
C1 1.0000 9.53742 5.02358 20.95327 Biso 1.000 C
C2 1.0000 8.52250 6.02911 20.77744 Biso 1.000 C
H1 1.0000 10.50915 5.23911 20.48278 Biso 1.000 H
H2 1.0000 8.74037 6.95146 20.23052 Biso 1.000 H
H3 1.0000 7.75131 6.14740 21.55008 Biso 1.000 H
C3 1.0000 9.62213 4.13414 22.16646 Biso 1.000 C
H4 1.0000 10.25317 4.60358 22.94319 Biso 1.000 H
H5 1.0000 10.07618 3.16099 21.92302 Biso 1.000 H
H6 1.0000 8.62969 3.95155 22.60394 Biso 1.000 H

RuSiMe3isopropyl_1x3-CeO2-211
data_image0
loop_
_atom_site_label
_atom_site_occupancy
_atom_site_Cartn_x
_atom_site_Cartn_y
_atom_site_Cartn_z
_atom_site_thermal_displace_type
_atom_site_B_iso_or_equiv
_atom_site_type_symbol
Ce1 1.0000 -0.77087 1.93685 10.23856 Biso 1.000 Ce
Ce2 1.0000 -0.75105 1.85652 16.85574 Biso 1.000 Ce
O1 1.0000 0.02059 1.93685 8.00000 Biso 1.000 O
O2 1.0000 1.60349 1.93685 10.23856 Biso 1.000 O
O3 1.0000 0.01546 1.92469 14.70723 Biso 1.000 O
Ce3 1.0000 5.56074 3.87550 9.11928 Biso 1.000 Ce
Ce4 1.0000 2.39494 1.93685 8.00000 Biso 1.000 Ce
O4 1.0000 4.76929 1.93685 8.00000 Biso 1.000 O
O5 1.0000 7.93510 3.87550 9.11928 Biso 1.000 O
O6 1.0000 3.18639 3.87550 9.11928 Biso 1.000 O
O7 1.0000 6.35219 1.93685 10.23856 Biso 1.000 O
Ce5 1.0000 2.39494 3.87550 11.35784 Biso 1.000 Ce
Ce6 1.0000 -0.78847 3.87527 13.54044 Biso 1.000 Ce
Ce7 1.0000 5.56074 1.93685 12.47712 Biso 1.000 Ce
O8 1.0000 7.93510 1.93685 12.47712 Biso 1.000 O
O9 1.0000 3.18639 1.93685 12.47712 Biso 1.000 O
O10 1.0000 4.76929 3.87550 11.35784 Biso 1.000 O
O11 1.0000 0.02059 3.87550 11.35784 Biso 1.000 O
Ce8 1.0000 2.39402 1.94664 14.69234 Biso 1.000 Ce
O12 1.0000 1.60019 3.87559 13.60663 Biso 1.000 O
O13 1.0000 6.32020 3.87571 13.57662 Biso 1.000 O
O14 1.0000 4.82506 1.93571 14.82265 Biso 1.000 O
Ce9 1.0000 5.58196 3.87720 15.90329 Biso 1.000 Ce

Ce10	1.0000	2.41617	3.87739	18.06234	Biso	1.000	Ce
O15	1.0000	1.54051	2.01345	16.95446	Biso	1.000	O
O16	1.0000	6.45383	1.89837	17.23916	Biso	1.000	O
O17	1.0000	7.94929	3.87392	15.87888	Biso	1.000	O
O18	1.0000	3.19043	3.87530	15.84990	Biso	1.000	O
Ce11	1.0000	5.30202	1.79938	19.21340	Biso	1.000	Ce
O19	1.0000	4.67207	3.87261	18.10649	Biso	1.000	O
O20	1.0000	-0.18402	3.82264	18.50308	Biso	1.000	O
O21	1.0000	3.07976	1.90136	19.23353	Biso	1.000	O
O22	1.0000	6.48033	3.92880	20.10367	Biso	1.000	O
Ce12	1.0000	-0.77087	5.81416	10.23856	Biso	1.000	Ce
Ce13	1.0000	-0.75074	5.88827	16.85761	Biso	1.000	Ce
O23	1.0000	0.02059	5.81416	8.00000	Biso	1.000	O
O24	1.0000	1.60349	5.81416	10.23856	Biso	1.000	O
O25	1.0000	0.01182	5.82665	14.70810	Biso	1.000	O
Ce14	1.0000	5.56074	7.75281	9.11928	Biso	1.000	Ce
Ce15	1.0000	2.39494	5.81416	8.00000	Biso	1.000	Ce
O26	1.0000	4.76929	5.81416	8.00000	Biso	1.000	O
O27	1.0000	7.93510	7.75281	9.11928	Biso	1.000	O
O28	1.0000	3.18639	7.75281	9.11928	Biso	1.000	O
O29	1.0000	6.35219	5.81416	10.23856	Biso	1.000	O
Ce16	1.0000	2.39494	7.75281	11.35784	Biso	1.000	Ce
Ce17	1.0000	-0.78094	7.75365	13.51883	Biso	1.000	Ce
Ce18	1.0000	5.56074	5.81416	12.47712	Biso	1.000	Ce
O30	1.0000	7.93510	5.81416	12.47712	Biso	1.000	O
O31	1.0000	3.18639	5.81416	12.47712	Biso	1.000	O
O32	1.0000	4.76929	7.75281	11.35784	Biso	1.000	O
O33	1.0000	0.02059	7.75281	11.35784	Biso	1.000	O
Ce19	1.0000	2.39250	5.80182	14.69189	Biso	1.000	Ce
O34	1.0000	1.59660	7.74979	13.57798	Biso	1.000	O
O35	1.0000	6.32020	7.75301	13.57662	Biso	1.000	O
O36	1.0000	4.82422	5.81596	14.82102	Biso	1.000	O
Ce20	1.0000	5.55783	7.75757	15.84030	Biso	1.000	Ce
Ce21	1.0000	2.30842	7.76298	17.99189	Biso	1.000	Ce
O37	1.0000	1.53191	5.72572	16.95234	Biso	1.000	O
O38	1.0000	6.45514	5.85617	17.23572	Biso	1.000	O
O39	1.0000	7.88246	7.75606	15.77888	Biso	1.000	O
O40	1.0000	3.10213	7.72249	15.80230	Biso	1.000	O
Ce22	1.0000	5.32223	5.97018	19.22610	Biso	1.000	Ce
O41	1.0000	4.61053	7.78520	17.93849	Biso	1.000	O
O42	1.0000	0.00287	7.77729	17.97735	Biso	1.000	O
O43	1.0000	3.09256	5.87643	19.19827	Biso	1.000	O
O44	1.0000	5.82352	7.78979	20.25809	Biso	1.000	O
Ce23	1.0000	-0.77087	9.69146	10.23856	Biso	1.000	Ce
Ce24	1.0000	-0.73867	9.69153	16.86375	Biso	1.000	Ce
O45	1.0000	0.02059	9.69146	8.00000	Biso	1.000	O
O46	1.0000	1.60349	9.69146	10.23856	Biso	1.000	O
O47	1.0000	0.01066	9.69111	14.70225	Biso	1.000	O
Ce25	1.0000	5.56074	11.63011	9.11928	Biso	1.000	Ce
Ce26	1.0000	2.39494	9.69146	8.00000	Biso	1.000	Ce
O48	1.0000	4.76929	9.69146	8.00000	Biso	1.000	O
O49	1.0000	7.93510	11.63011	9.11928	Biso	1.000	O
O50	1.0000	3.18639	11.63011	9.11928	Biso	1.000	O
O51	1.0000	6.35219	9.69146	10.23856	Biso	1.000	O
Ce27	1.0000	2.39494	11.63011	11.35784	Biso	1.000	Ce
Ce28	1.0000	-0.78124	11.62927	13.51800	Biso	1.000	Ce
Ce29	1.0000	5.56074	9.69146	12.47712	Biso	1.000	Ce
O52	1.0000	7.93510	9.69146	12.47712	Biso	1.000	O
O53	1.0000	3.18639	9.69146	12.47712	Biso	1.000	O
O54	1.0000	4.76929	11.63011	11.35784	Biso	1.000	O

O55 1.0000 0.02059 11.63011 11.35784 Biso 1.000 O
Ce30 1.0000 2.37569 9.69222 14.66741 Biso 1.000 Ce
O56 1.0000 1.59601 11.63215 13.57661 Biso 1.000 O
O57 1.0000 6.32020 11.63032 13.57662 Biso 1.000 O
O58 1.0000 4.79843 9.69126 14.81593 Biso 1.000 O
Ce31 1.0000 5.55466 11.62584 15.84147 Biso 1.000 Ce
Ce32 1.0000 2.29454 11.62321 17.99770 Biso 1.000 Ce
O59 1.0000 1.59625 9.69459 16.88306 Biso 1.000 O
O60 1.0000 6.47213 9.69950 17.24034 Biso 1.000 O
O61 1.0000 7.88198 11.62728 15.77476 Biso 1.000 O
O62 1.0000 3.10188 11.65737 15.79842 Biso 1.000 O
Ce33 1.0000 5.26311 9.69864 19.24316 Biso 1.000 Ce
O63 1.0000 4.59066 11.62278 17.94085 Biso 1.000 O
O64 1.0000 -0.01161 11.59694 17.98415 Biso 1.000 O
O65 1.0000 3.01116 9.69690 19.18693 Biso 1.000 O
O66 1.0000 5.86990 11.59229 20.21969 Biso 1.000 O
Ru1 1.0000 8.31932 3.70270 20.11157 Biso 1.000 Ru
Si1 1.0000 9.13208 5.33204 21.65643 Biso 1.000 Si
C1 1.0000 10.98387 5.03661 21.97416 Biso 1.000 C
H1 1.0000 11.27330 5.63195 22.85743 Biso 1.000 H
H2 1.0000 11.58286 5.39488 21.12294 Biso 1.000 H
H3 1.0000 11.25060 3.98941 22.17597 Biso 1.000 H
C2 1.0000 8.95323 7.13285 21.13650 Biso 1.000 C
H4 1.0000 7.90355 7.46281 21.16571 Biso 1.000 H
H5 1.0000 9.32727 7.34334 20.12571 Biso 1.000 H
H6 1.0000 9.52913 7.74375 21.85399 Biso 1.000 H
C3 1.0000 8.20313 5.19621 23.30728 Biso 1.000 C
H7 1.0000 7.11810 5.30438 23.16112 Biso 1.000 H
H8 1.0000 8.53684 6.02859 23.94947 Biso 1.000 H
H9 1.0000 8.38938 4.25660 23.84444 Biso 1.000 H
H10 1.0000 6.97298 2.72066 22.71231 Biso 1.000 H
C4 1.0000 8.85153 2.33831 21.62121 Biso 1.000 C
C5 1.0000 7.59597 1.88398 22.36889 Biso 1.000 C
H11 1.0000 9.51788 2.84509 22.33698 Biso 1.000 H
H12 1.0000 7.90513 1.31641 23.26692 Biso 1.000 H
H13 1.0000 6.98744 1.19765 21.76046 Biso 1.000 H
C6 1.0000 9.60383 1.16443 21.01144 Biso 1.000 C
H14 1.0000 9.87071 0.44482 21.80999 Biso 1.000 H
H15 1.0000 10.54826 1.48517 20.54250 Biso 1.000 H
H16 1.0000 9.00327 0.61769 20.27060 Biso 1.000 H

TS_RuHSiMe3propen_RuSiMe3isopropyl_1x3-CeO2-211
data_image0
loop_
_atom_site_label
_atom_site_occupancy
_atom_site_Cartn_x
_atom_site_Cartn_y
_atom_site_Cartn_z
_atom_site_thermal_displace_type
_atom_site_B_iso_or_equiv
_atom_site_type_symbol
Ce1 1.0000 -0.77087 1.93685 10.23856 Biso 1.000 Ce
Ce2 1.0000 -0.73857 1.88774 16.86065 Biso 1.000 Ce
O1 1.0000 0.02059 1.93685 8.00000 Biso 1.000 O
O2 1.0000 1.60349 1.93685 10.23856 Biso 1.000 O
O3 1.0000 0.01968 1.92778 14.71218 Biso 1.000 O
Ce3 1.0000 5.56074 3.87550 9.11928 Biso 1.000 Ce
Ce4 1.0000 2.39494 1.93685 8.00000 Biso 1.000 Ce

O4	1.0000	4.76929	1.93685	8.00000	Biso	1.000	O
O5	1.0000	7.93510	3.87550	9.11928	Biso	1.000	O
O6	1.0000	3.18639	3.87550	9.11928	Biso	1.000	O
O7	1.0000	6.35219	1.93685	10.23856	Biso	1.000	O
Ce5	1.0000	2.39494	3.87550	11.35784	Biso	1.000	Ce
Ce6	1.0000	-0.78741	3.87633	13.53939	Biso	1.000	Ce
Ce7	1.0000	5.56074	1.93685	12.47712	Biso	1.000	Ce
O8	1.0000	7.93510	1.93685	12.47712	Biso	1.000	O
O9	1.0000	3.18639	1.93685	12.47712	Biso	1.000	O
O10	1.0000	4.76929	3.87550	11.35784	Biso	1.000	O
O11	1.0000	0.02059	3.87550	11.35784	Biso	1.000	O
Ce8	1.0000	2.39537	1.94707	14.68982	Biso	1.000	Ce
O12	1.0000	1.59857	3.87600	13.59775	Biso	1.000	O
O13	1.0000	6.32020	3.87571	13.57662	Biso	1.000	O
O14	1.0000	4.82706	1.94006	14.82133	Biso	1.000	O
Ce9	1.0000	5.57210	3.87785	15.88820	Biso	1.000	Ce
Ce10	1.0000	2.37166	3.88783	18.06213	Biso	1.000	Ce
O15	1.0000	1.56535	1.99599	16.94143	Biso	1.000	O
O16	1.0000	6.45478	1.90475	17.22098	Biso	1.000	O
O17	1.0000	7.92525	3.86562	15.84178	Biso	1.000	O
O18	1.0000	3.17332	3.87838	15.83274	Biso	1.000	O
Ce11	1.0000	5.30996	1.83418	19.20648	Biso	1.000	Ce
O19	1.0000	4.64322	3.86537	18.05673	Biso	1.000	O
O20	1.0000	-0.11425	3.86871	18.32582	Biso	1.000	O
O21	1.0000	3.08166	1.91533	19.22557	Biso	1.000	O
O22	1.0000	6.47242	3.91779	20.01372	Biso	1.000	O
Ce12	1.0000	-0.77087	5.81416	10.23856	Biso	1.000	Ce
Ce13	1.0000	-0.74759	5.86106	16.87120	Biso	1.000	Ce
O23	1.0000	0.02059	5.81416	8.00000	Biso	1.000	O
O24	1.0000	1.60349	5.81416	10.23856	Biso	1.000	O
O25	1.0000	0.01537	5.82161	14.71586	Biso	1.000	O
Ce14	1.0000	5.56074	7.75281	9.11928	Biso	1.000	Ce
Ce15	1.0000	2.39494	5.81416	8.00000	Biso	1.000	Ce
O26	1.0000	4.76929	5.81416	8.00000	Biso	1.000	O
O27	1.0000	7.93510	7.75281	9.11928	Biso	1.000	O
O28	1.0000	3.18639	7.75281	9.11928	Biso	1.000	O
O29	1.0000	6.35219	5.81416	10.23856	Biso	1.000	O
Ce16	1.0000	2.39494	7.75281	11.35784	Biso	1.000	Ce
Ce17	1.0000	-0.78238	7.75357	13.52837	Biso	1.000	Ce
Ce18	1.0000	5.56074	5.81416	12.47712	Biso	1.000	Ce
O30	1.0000	7.93510	5.81416	12.47712	Biso	1.000	O
O31	1.0000	3.18639	5.81416	12.47712	Biso	1.000	O
O32	1.0000	4.76929	7.75281	11.35784	Biso	1.000	O
O33	1.0000	0.02059	7.75281	11.35784	Biso	1.000	O
Ce19	1.0000	2.39187	5.80749	14.69112	Biso	1.000	Ce
O34	1.0000	1.59409	7.75076	13.57639	Biso	1.000	O
O35	1.0000	6.32020	7.75301	13.57662	Biso	1.000	O
O36	1.0000	4.82112	5.81216	14.81417	Biso	1.000	O
Ce20	1.0000	5.55974	7.75527	15.84731	Biso	1.000	Ce
Ce21	1.0000	2.30556	7.75909	18.00734	Biso	1.000	Ce
O37	1.0000	1.56765	5.75689	16.94928	Biso	1.000	O
O38	1.0000	6.43060	5.84360	17.19611	Biso	1.000	O
O39	1.0000	7.88206	7.76338	15.78444	Biso	1.000	O
O40	1.0000	3.10757	7.73361	15.79481	Biso	1.000	O
Ce22	1.0000	5.33148	5.92171	19.21011	Biso	1.000	Ce
O41	1.0000	4.59506	7.77304	17.93962	Biso	1.000	O
O42	1.0000	0.00377	7.78763	17.98743	Biso	1.000	O
O43	1.0000	3.08263	5.83302	19.23113	Biso	1.000	O
O44	1.0000	5.91523	7.78144	20.19779	Biso	1.000	O
Ce23	1.0000	-0.77087	9.69146	10.23856	Biso	1.000	Ce

Ce24 1.0000 -0.73164 9.69249 16.87790 Biso 1.000 Ce
O45 1.0000 0.02059 9.69146 8.00000 Biso 1.000 O
O46 1.0000 1.60349 9.69146 10.23856 Biso 1.000 O
O47 1.0000 0.01431 9.69157 14.70998 Biso 1.000 O
Ce25 1.0000 5.56074 11.63011 9.11928 Biso 1.000 Ce
Ce26 1.0000 2.39494 9.69146 8.00000 Biso 1.000 Ce
O48 1.0000 4.76929 9.69146 8.00000 Biso 1.000 O
O49 1.0000 7.93510 11.63011 9.11928 Biso 1.000 O
O50 1.0000 3.18639 11.63011 9.11928 Biso 1.000 O
O51 1.0000 6.35219 9.69146 10.23856 Biso 1.000 O
Ce27 1.0000 2.39494 11.63011 11.35784 Biso 1.000 Ce
Ce28 1.0000 -0.77982 11.62800 13.52431 Biso 1.000 Ce
Ce29 1.0000 5.56074 9.69146 12.47712 Biso 1.000 Ce
O52 1.0000 7.93510 9.69146 12.47712 Biso 1.000 O
O53 1.0000 3.18639 9.69146 12.47712 Biso 1.000 O
O54 1.0000 4.76929 11.63011 11.35784 Biso 1.000 O
O55 1.0000 0.02059 11.63011 11.35784 Biso 1.000 O
Ce30 1.0000 2.38026 9.69080 14.67064 Biso 1.000 Ce
O56 1.0000 1.59451 11.63220 13.57586 Biso 1.000 O
O57 1.0000 6.32020 11.63032 13.57662 Biso 1.000 O
O58 1.0000 4.80822 9.69191 14.81682 Biso 1.000 O
Ce31 1.0000 5.56431 11.62530 15.84622 Biso 1.000 Ce
Ce32 1.0000 2.31106 11.62219 17.99312 Biso 1.000 Ce
O59 1.0000 1.59962 9.69972 16.89065 Biso 1.000 O
O60 1.0000 6.47263 9.69798 17.23947 Biso 1.000 O
O61 1.0000 7.89361 11.62654 15.78648 Biso 1.000 O
O62 1.0000 3.11390 11.65105 15.78869 Biso 1.000 O
Ce33 1.0000 5.28064 9.68948 19.24608 Biso 1.000 Ce
O63 1.0000 4.59966 11.62151 17.93364 Biso 1.000 O
O64 1.0000 0.00327 11.62633 18.02608 Biso 1.000 O
O65 1.0000 3.02796 9.69433 19.19549 Biso 1.000 O
O66 1.0000 5.86015 11.62778 20.21561 Biso 1.000 O
Ru1 1.0000 8.41597 3.70543 20.12709 Biso 1.000 Ru
Si1 1.0000 8.87175 5.27166 21.87962 Biso 1.000 Si
C1 1.0000 10.16712 4.76587 23.17256 Biso 1.000 C
H1 1.0000 10.26420 5.60164 23.88573 Biso 1.000 H
H2 1.0000 11.15759 4.60662 22.71842 Biso 1.000 H
H3 1.0000 9.90478 3.86918 23.75177 Biso 1.000 H
C2 1.0000 9.54539 6.86764 21.12891 Biso 1.000 C
H4 1.0000 8.92738 7.27390 20.31780 Biso 1.000 H
H5 1.0000 10.56387 6.68514 20.74878 Biso 1.000 H
H6 1.0000 9.60551 7.63056 21.92389 Biso 1.000 H
C3 1.0000 7.25374 5.65508 22.79200 Biso 1.000 C
H7 1.0000 6.63607 6.33818 22.18694 Biso 1.000 H
H8 1.0000 7.48131 6.16719 23.74212 Biso 1.000 H
H9 1.0000 6.67365 4.74960 23.01794 Biso 1.000 H
H10 1.0000 9.97550 3.79230 20.64719 Biso 1.000 H
C4 1.0000 8.42401 1.88289 21.16194 Biso 1.000 C
C5 1.0000 9.80866 2.25094 20.94780 Biso 1.000 C
H11 1.0000 8.03220 1.12027 20.46950 Biso 1.000 H
H12 1.0000 10.37409 1.74071 20.16359 Biso 1.000 H
H13 1.0000 10.42456 2.44225 21.83345 Biso 1.000 H
C6 1.0000 7.82002 1.76144 22.53912 Biso 1.000 C
H14 1.0000 8.02103 0.75358 22.94383 Biso 1.000 H
H15 1.0000 6.72673 1.87579 22.51114 Biso 1.000 H
H16 1.0000 8.22787 2.50122 23.24131 Biso 1.000 H

TS_Rupropen_RuHallyl_1x3-CeO2-211
data_image0

```
loop_
  _atom_site_label
  _atom_site_occupancy
  _atom_site_Cartn_x
  _atom_site_Cartn_y
  _atom_site_Cartn_z
  _atom_site_thermal_displace_type
  _atom_site_B_iso_or_equiv
  _atom_site_type_symbol
  Ce1      1.0000 -0.77087  1.93685  10.23856  Biso   1.000  Ce
  Ce2      1.0000 -0.73848  1.89247  16.88574  Biso   1.000  Ce
  O1       1.0000 0.02059  1.93685  8.00000  Biso   1.000  O
  O2       1.0000 1.60349  1.93685  10.23856  Biso   1.000  O
  O3       1.0000 0.01349  1.93147  14.71620  Biso   1.000  O
  Ce3      1.0000 5.56074  3.87550  9.11928  Biso   1.000  Ce
  Ce4      1.0000 2.39494  1.93685  8.00000  Biso   1.000  Ce
  O4       1.0000 4.76929  1.93685  8.00000  Biso   1.000  O
  O5       1.0000 7.93510  3.87550  9.11928  Biso   1.000  O
  O6       1.0000 3.18639  3.87550  9.11928  Biso   1.000  O
  O7       1.0000 6.35219  1.93685  10.23856  Biso   1.000  O
  Ce5      1.0000 2.39494  3.87550  11.35784  Biso   1.000  Ce
  Ce6      1.0000 -0.78540  3.87677  13.54490  Biso   1.000  Ce
  Ce7      1.0000 5.56074  1.93685  12.47712  Biso   1.000  Ce
  O8       1.0000 7.93510  1.93685  12.47712  Biso   1.000  O
  O9       1.0000 3.18639  1.93685  12.47712  Biso   1.000  O
  O10      1.0000 4.76929  3.87550  11.35784  Biso   1.000  O
  O11      1.0000 0.02059  3.87550  11.35784  Biso   1.000  O
  Ce8      1.0000 2.39147  1.93622  14.68206  Biso   1.000  Ce
  O12      1.0000 1.59460  3.87636  13.59296  Biso   1.000  O
  O13      1.0000 6.32020  3.87571  13.57662  Biso   1.000  O
  O14      1.0000 4.82181  1.93913  14.80670  Biso   1.000  O
  Ce9      1.0000 5.57757  3.87745  15.87201  Biso   1.000  Ce
  Ce10     1.0000 2.39929  3.86957  18.00556  Biso   1.000  Ce
  O15      1.0000 1.59078  1.98010  16.92401  Biso   1.000  O
  O16      1.0000 6.42762  1.89742  17.17907  Biso   1.000  O
  O17      1.0000 7.91082  3.88569  15.84390  Biso   1.000  O
  O18      1.0000 3.17692  3.87631  15.80780  Biso   1.000  O
  Ce11     1.0000 5.33975  1.81888  19.19259  Biso   1.000  Ce
  O19      1.0000 4.65438  3.89412  18.02547  Biso   1.000  O
  O20      1.0000 -0.06510  3.86934  18.23412  Biso   1.000  O
  O21      1.0000 3.08860  1.92714  19.21403  Biso   1.000  O
  O22      1.0000 6.43438  3.91795  19.98605  Biso   1.000  O
  Ce12     1.0000 -0.77087  5.81416  10.23856  Biso   1.000  Ce
  Ce13     1.0000 -0.74390  5.84161  16.90620  Biso   1.000  Ce
  O23      1.0000 0.02059  5.81416  8.00000  Biso   1.000  O
  O24      1.0000 1.60349  5.81416  10.23856  Biso   1.000  O
  O25      1.0000 0.01035  5.81881  14.72624  Biso   1.000  O
  Ce14     1.0000 5.56074  7.75281  9.11928  Biso   1.000  Ce
  Ce15     1.0000 2.39494  5.81416  8.00000  Biso   1.000  Ce
  O26      1.0000 4.76929  5.81416  8.00000  Biso   1.000  O
  O27      1.0000 7.93510  7.75281  9.11928  Biso   1.000  O
  O28      1.0000 3.18639  7.75281  9.11928  Biso   1.000  O
  O29      1.0000 6.35219  5.81416  10.23856  Biso   1.000  O
  Ce16     1.0000 2.39494  7.75281  11.35784  Biso   1.000  Ce
  Ce17     1.0000 -0.78056  7.75168  13.53427  Biso   1.000  Ce
  Ce18     1.0000 5.56074  5.81416  12.47712  Biso   1.000  Ce
  O30      1.0000 7.93510  5.81416  12.47712  Biso   1.000  O
  O31      1.0000 3.18639  5.81416  12.47712  Biso   1.000  O
  O32      1.0000 4.76929  7.75281  11.35784  Biso   1.000  O
  O33      1.0000 0.02059  7.75281  11.35784  Biso   1.000  O
```

Ce19	1.0000	2.38710	5.81230	14.68261	Biso	1.000	Ce
O34	1.0000	1.59205	7.74861	13.57419	Biso	1.000	O
O35	1.0000	6.32020	7.75301	13.57662	Biso	1.000	O
O36	1.0000	4.82468	5.81443	14.80896	Biso	1.000	O
Ce20	1.0000	5.57211	7.75720	15.84245	Biso	1.000	Ce
Ce21	1.0000	2.30870	7.76207	17.99427	Biso	1.000	Ce
O37	1.0000	1.57439	5.76644	16.92397	Biso	1.000	O
O38	1.0000	6.43521	5.86625	17.18646	Biso	1.000	O
O39	1.0000	7.90029	7.75851	15.79655	Biso	1.000	O
O40	1.0000	3.11855	7.73770	15.78346	Biso	1.000	O
Ce22	1.0000	5.31058	5.94621	19.19177	Biso	1.000	Ce
O41	1.0000	4.59763	7.78017	17.93175	Biso	1.000	O
O42	1.0000	0.01201	7.75410	18.03562	Biso	1.000	O
O43	1.0000	3.06718	5.84111	19.21176	Biso	1.000	O
O44	1.0000	5.89651	7.75114	20.20736	Biso	1.000	O
Ce23	1.0000	-0.77087	9.69146	10.23856	Biso	1.000	Ce
Ce24	1.0000	-0.72819	9.68979	16.88658	Biso	1.000	Ce
O45	1.0000	0.02059	9.69146	8.00000	Biso	1.000	O
O46	1.0000	1.60349	9.69146	10.23856	Biso	1.000	O
O47	1.0000	0.01817	9.68906	14.71171	Biso	1.000	O
Ce25	1.0000	5.56074	11.63011	9.11928	Biso	1.000	Ce
Ce26	1.0000	2.39494	9.69146	8.00000	Biso	1.000	Ce
O48	1.0000	4.76929	9.69146	8.00000	Biso	1.000	O
O49	1.0000	7.93510	11.63011	9.11928	Biso	1.000	O
O50	1.0000	3.18639	11.63011	9.11928	Biso	1.000	O
O51	1.0000	6.35219	9.69146	10.23856	Biso	1.000	O
Ce27	1.0000	2.39494	11.63011	11.35784	Biso	1.000	Ce
Ce28	1.0000	-0.78186	11.62880	13.53159	Biso	1.000	Ce
Ce29	1.0000	5.56074	9.69146	12.47712	Biso	1.000	Ce
O52	1.0000	7.93510	9.69146	12.47712	Biso	1.000	O
O53	1.0000	3.18639	9.69146	12.47712	Biso	1.000	O
O54	1.0000	4.76929	11.63011	11.35784	Biso	1.000	O
O55	1.0000	0.02059	11.63011	11.35784	Biso	1.000	O
Ce30	1.0000	2.38356	9.69120	14.67193	Biso	1.000	Ce
O56	1.0000	1.59300	11.63145	13.57392	Biso	1.000	O
O57	1.0000	6.32020	11.63032	13.57662	Biso	1.000	O
O58	1.0000	4.81653	9.69002	14.81197	Biso	1.000	O
Ce31	1.0000	5.56814	11.62768	15.84362	Biso	1.000	Ce
Ce32	1.0000	2.31205	11.62481	18.00367	Biso	1.000	Ce
O59	1.0000	1.59735	9.68636	16.89128	Biso	1.000	O
O60	1.0000	6.48104	9.68907	17.24208	Biso	1.000	O
O61	1.0000	7.89589	11.61105	15.79049	Biso	1.000	O
O62	1.0000	3.11736	11.63999	15.78358	Biso	1.000	O
Ce33	1.0000	5.28678	9.69910	19.25059	Biso	1.000	Ce
O63	1.0000	4.60047	11.60911	17.93166	Biso	1.000	O
O64	1.0000	0.02196	11.60314	18.02672	Biso	1.000	O
O65	1.0000	3.02876	9.69210	19.19550	Biso	1.000	O
O66	1.0000	5.89085	11.59064	20.20748	Biso	1.000	O
Ru1	1.0000	8.33371	3.96114	19.99567	Biso	1.000	Ru
H1	1.0000	9.41748	2.79712	20.34515	Biso	1.000	H
C1	1.0000	9.72092	5.16172	21.03310	Biso	1.000	C
C2	1.0000	8.41156	5.36604	21.58792	Biso	1.000	C
H2	1.0000	10.27318	5.92379	20.47837	Biso	1.000	H
H3	1.0000	7.90429	6.31916	21.39954	Biso	1.000	H
H4	1.0000	8.14130	4.87150	22.52884	Biso	1.000	H
C3	1.0000	10.22735	3.82551	21.16976	Biso	1.000	C
H5	1.0000	11.21776	3.58075	20.76605	Biso	1.000	H
H6	1.0000	10.03004	3.28832	22.10526	Biso	1.000	H
